# Lead Structure-Based Hybridization Strategy Reveals Major Potency Enhancement of SirReal-Type Sirt2 Inhibitors

**DOI:** 10.3390/ijms26209855

**Published:** 2025-10-10

**Authors:** Matthias Frei, Ricky Wirawan, Thomas Wein, Franz Bracher

**Affiliations:** Department of Pharmacy, Center for Drug Research, Ludwig-Maximilians University, Butenandtstr. 5–13, 81377 Munich, Germanyricky.wirawan@cup.uni-muenchen.de (R.W.);

**Keywords:** sirtuin 2 inhibitor, Sirt2 inhibitor, SirReal2, structure–activity relationship, hybridization strategy, highly potent and selective Sirt2 inhibitor, structural optimization

## Abstract

Selective and potent inhibitors of the NAD^+^-dependent deacetylase Sirt2 represent a valuable epigenetic strategy for the treatment of currently incurable diseases such as Parkinson’s disease, Huntington’s disease, Alzheimer’s disease, and multiple sclerosis. Guided by molecular docking and MM/GBSA validation studies, a lead structure-based hybridization strategy was developed, resulting in a series of very effective Sirt2 inhibitors. With **RW-93**, we present a highly potent and subtype selective Sirt2 inhibitor (IC_50_ = 16 nM), which as a next generation SirReal-type inhibitor significantly surpasses established Sirt2 inhibitors and contributes to the extension of the current SAR profile. The structural modification strategy employed in this study proved to be highly promising, resulting in the identification of the most potent low-molecular-weight Sirt2 inhibitor reported to date, providing a promising target for further medicinal chemistry-driven SAR studies.

## 1. Introduction

Histone modifications take a key position in epigenetic regulation processes, essentially controlled by acetylation and deacetylation of lysine on *N*-terminal histone tails catalyzed by histone acetyltransferases (HATs) and histone deacetylases (HDACs) [1,2,3]. HDACs are generally categorized into 4 classes (class I-IV), with class III being referred to as sirtuins. Sirtuins are divided into 7 subtypes (Sirt1-7) based on different cell function and localization and, in contrast to the other HDACs, are not zinc-dependent but require NAD^+^ as a cofactor for catalytic activity [4,5].

**Figure 1:**
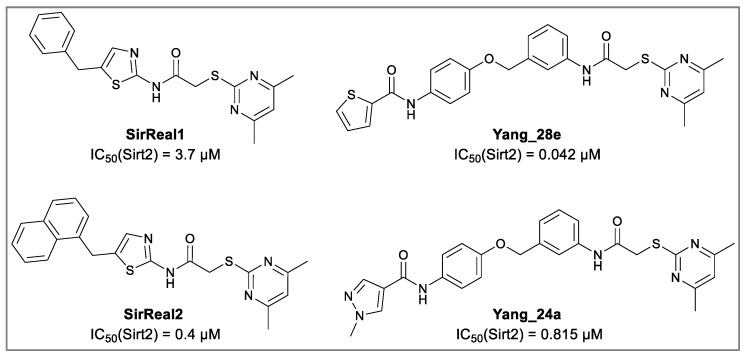
Chemical structures and inhibitory activities of selected Sirt2 inhibitors based on the SirReal feature. The originally reported IC_50_ values were determined under differing assay conditions, precluding direct comparability. Compound **Yang_28e** was reported to exhibit greater potency than **SirReal2**, while compound **Yang_24a** constitutes a further variation of **Yang_28e**.

Sirtuins play a fundamental role in pathophysiological mechanisms, making them a significant therapeutic target of ongoing intensive research. Selective inhibition of Sirt2 is associated with numerous positive effects such as anti-angiogenesis, neuroprotection and anti-inflammation, and represents a valuable tool in understanding and treating related diseases [6,7,8,9,10]. Selective Sirt2 inhibitors, which are chemically and structurally diverse, display various binding modes and mechanisms that target the C-pocket, the extended C-site including the selectivity pocket, the substrate channel, or the cofactor NAD^+^ [11]. The development of sirtuin rearranging ligands (SirReals) as potent inhibitors that selectively inhibit Sirt2 by inducing a hydrophobic selectivity pocket during binding, laid the foundation for extensive structure–activity relationship studies and inspired further development of corresponding inhibitors (Figure 1) [12,13].

SirReal-type inhibitors share a 2-((4,6-dimethylpyrimidin-2-yl)thio)acetamide moiety as characteristic structural feature, which occupies the hydrophobic selectivity pocket induced after binding of the inhibitor in the active site and thus significantly determines Sirt2 selectivity and enables corresponding potency. First generation benchmark **SirReal2** developed by Rumpf et al. shows intense van der Waals interactions within the substrate channel based on the corresponding naphthalene residue. Various aromatic (Phe131, Phe234) and aliphatic amino acids (Leu134, Ile169, Ile232, Val233) as well as the nicotinamide moiety of NAD^+^ are being addressed [14]. Subsequently Schiedel et al. developed further optimized compounds based on the initial SirReal-type inhibitors, providing valuable structure–activity relationships [15,16]. With Sirt2 inhibitors **Yang_28e** [17] and **Yang_24a** [18], Yang et al. presented advanced SirReal-derivatives that demonstrate an even stronger inhibitory effect and high selectivity, providing a comprehensive contribution and solid foundation for enabling profounded SAR studies. The recurring 2-((4,6-dimethylpyrimidin-2-yl)thio)acetamide moiety guarantees high Sirt2 selectivity by binding into the in situ induced selectivity pocket and ensures a comparable general alignment of the SirReal-type inhibitors within the binding pocket. The *N*-phenylthiophene-2-carboxamide moiety in **Yang_28e** and the 1-methyl-*N*-phenyl-1*H*-pyrazole-4-carboxamide moiety in **Yang_24a**, which are linked by a benzyl ether structure, enable further and more intense π-π interactions (e.g., with Phe235) and hydrophilic interactions (H-bond with Val233) within the substrate channel of Sirt2 [17,19].

**Figure 2:**
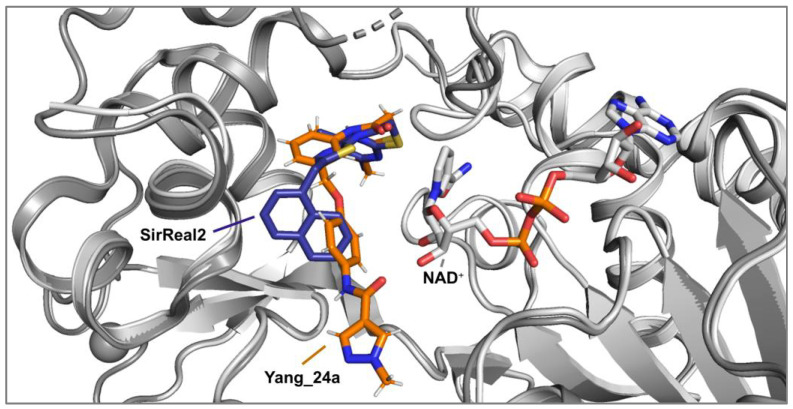
Crystal structure superimposition of **SirReal2** (purple) and cofactor NAD^+^ (grey, PDB ID: 4RMG) with compound **Yang_24a** (orange, taken from PDB ID: 5YQO).

**Scheme 1:**
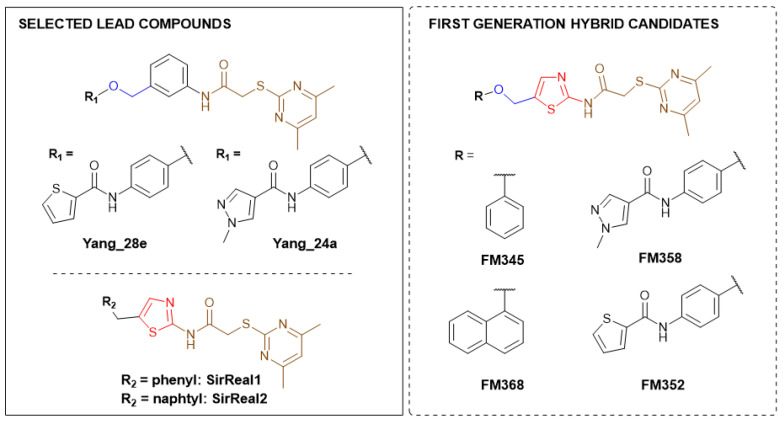
General hybridization approach based on lead compounds **SirReal2** and **Yang_28e** generating four first round (aryloxymethyl)thiazole candidates **FM345**, **FM358**, **FM368** and **FM352**.

Initial analysis of the superposition (Figure 2) of the crystal structures of the lead compounds **SirReal2** and **Yang_24a** revealed structural similarities and several characteristic elements, forming the fundament for the development of the envisaged hybridization strategy. In continuation of our work in the synthesis and biological evaluation of Sirt2 inhibitors [20], the fundamental approach was to integrate the amide-based channel binding residue of **Yang_24a** into the thiazole structure of **SirReal2** and link it via a benzyl ether bridge instead of the methylene bridge, generating hybrid candidate **FM358**. Further structural features such as the benzene (**SirReal1**) and naphthalene rings (**SirReal2**) and the *N*-phenylthiophene-2-carboxamide moiety (**Yang_28e**) of other lead structures were selected to generate hybrid candidates **FM345**, **FM368** and **FM352** (Scheme 1).

The novel (aryloxymethyl)thiazolamide structure thus considered should be able to orient the 1-methyl-*N*-phenyl-1*H*-pyrazole-4-carboxamide residue of the pharmacophore more favourably by changing the spatial angle and increasing the structural flexibility, thereby blocking the function of cofactor NAD^+^ more effectively and therefore increasing the inhibitory activity. The most promising hybrid candidate **FM358** derived from this hybridization approach of **SirReal2** and **Yang_24a** was to be extended by the design of further variations to enable a more comprehensive structure–activity relationship study.

## 2. Results

### 2.1. Initial Docking Experiments for the Validation of the Hybridization Concept

**Figure 3:**
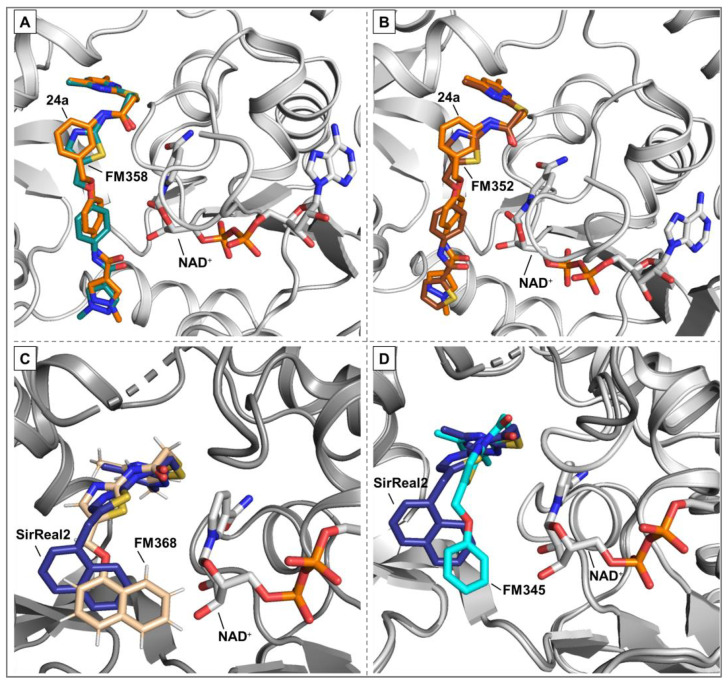
(**A**) Docking pose of hybrid **FM358** (green) based on the co-crystal structure of lead compound **Yang_24a** (orange, PDB ID: 5YQO). The position of cofactor NAD^+^ (grey) was copied from PDB ID: 4RMG for visualization, as not included in the crystal structure of **Yang_24a** (PDB ID: 5YQO). (**B**) Docking pose of hybrid **FM352** (brown) based on co-crystal structure of lead compound **Yang_24a** (orange, PDB ID: 5YQO). The position of cofactor NAD^+^ (grey) was copied from PDB ID: 4RMG for visualization, as not included in the crystal structure of **Yang_24a** (PDB ID: 5YQO). (**C**) Docking pose of hybrid **FM368** (cream) based on co-crystal structure of lead compound **SirReal2** (blue, PDB ID: 4RMG) and cofactor NAD^+^ (grey). (**D**) Docking pose of hybrid **FM345** (cyan) based on the co-crystal structure of lead compound **SirReal2** (blue, PDB ID: 4RMG) and NAD^+^ (grey).

Guided by docking experiments, the resulting library of four hybridized target compounds (Scheme 1) was evaluated for its inhibitory potential and the predicted spatial arrangement was analysed in relation to the orientation of the respective substrate channel residues. The docking study carried out with the envisaged hybrid candidates shows encouraging results (Figure 3).

All predicted poses show a principal correlation with the co-crystal structures of the lead compounds including a high overlap of the selectivity pocket binder motif, an essential prerequisite for the Sirt2 selectivity of the featured SirReal-based inhibitors. The calculated spatial orientations of **FM358** and **FM352** are very similar to each other and to the co-crystal structure of **Yang_24a**, only the amide-based substrate channel residue of both hybrid candidates is slightly shifted. **FM345** and **FM368** differ mainly in the position of benzene and naphthalene rings, which in the prediction occupy a more central position in the substrate channel, closer to cofactor NAD^+^, compared to **SirReal2**, which is mainly mediated by the higher flexibility of the benzyl ether.

Consequently, the docking experiments show promising predictions indicating high potency, therefore a strategy for the synthesis of these hybrid target compounds was developed.

### 2.2. Synthesis of First Generation Hybrid Candidates

**Scheme 2:**
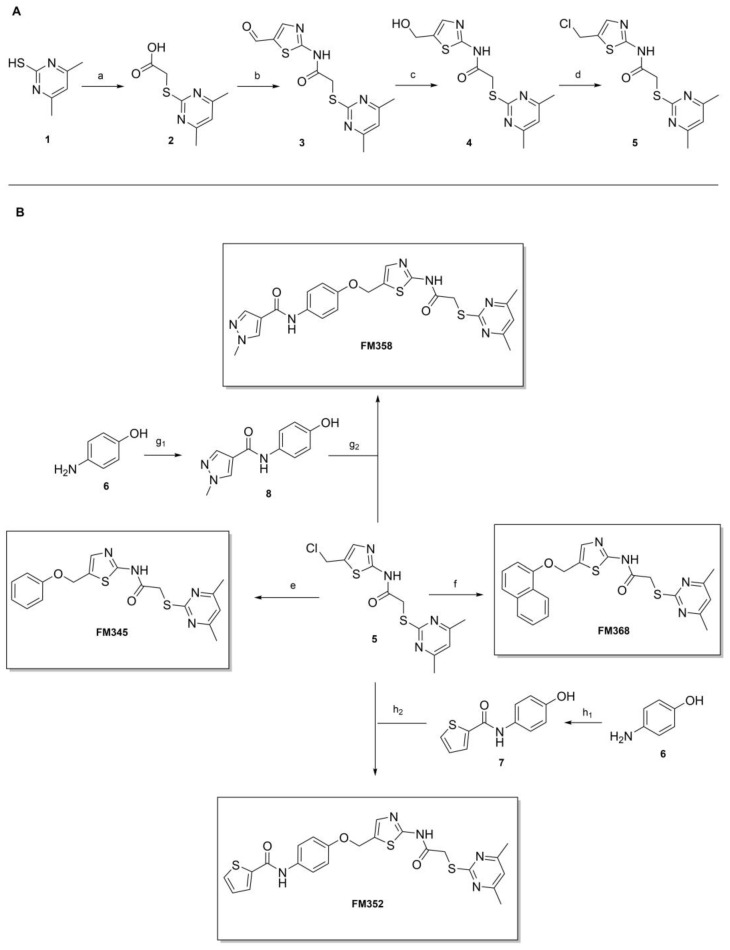
(**A**) (a) 2-chloroacetic acid, NEt_3_, acetonitrile, rt, 24 h, 81% for **2**; (b) 2-aminothiazole-5-carbaldehyde, DMAP, EDC·HCl, DMF, rt, 16 h, 46% for **3**; (c) NaBH_4_, MeOH, 3 h, 0 °C, quantitative for **4**; (d) SOCl_2_, DCM, 18 h, rt, quantitative for **5**. (**B**) (e) sodium phenoxide, rt, 24 h, DCM, 18% for **FM345**; (f) 1-naphthol, NaOMe 25% (*w*/*w*) in MeOH, rt, 2 min, DCM, crude for; then **5**, acetone, rt, 5 h, 14% for **FM368**; (g_1_) 1-methyl-1*H*-pyrazole-4-carboxylic acid, DIPEA, HATU, THF, 3 h, rt, 21% for **8**; (g_2_) NaOMe 25% (*w*/*w*) in MeOH, rt, 2 min, DCM, crude; then **5**, DCM, rt, 17% for **FM358**; (h_1_) thiophene-2-carbonyl chloride, pyridine, DMF, 1.5 h, rt, 69% for **7**; (h_2_) NaOMe 25% (*w*/*w*) in MeOH, rt, 2 min, DCM, crude; then **5**, DCM, rt, 38% for **FM352**.

The preparation of the desired hybrid target compounds **FM345**, **FM358**, **FM368** and **FM352** was achieved in five steps each, requiring the establishment of a general synthesis approach to the previously unknown structural class of 2-acylamino-5-(aryloxymethyl)thiazoles (see Scheme 2). 2-((4,6-Dimethylpyrimidin-2-yl)thio)acetic acid (**2**) was obtained by base-mediated thioether synthesis of 4,6-dimethylpyrimidine-2-thiol (**1**) and 2-chloroacetic acid, with subsequent EDC·HCl mediated amidation with 2-aminothiazole-5-carbaldehyde to amide **3**. Reduction of the aldehyde group in compound **3** using sodium borohydride gave the primary alcohol **4**, which was subsequently converted to the corresponding alkyl chloride **5** using thionyl chloride. The Williamson ether synthesis to the previously unknown 5-(aryloxymethyl)thiazoles failed under a standard setup with conventional reaction conditions due to C-alkylation tendencies at the electron-rich phenolates, which is why after extensive experimentation a protocol using preformed sodium phenolates was established, allowing the preparation of the target compounds **FM345**, **FM352**, **FM358** and **FM368**.

Phenyl ether **FM345** was synthesized using alkyl chloride **5** and commercially available sodium phenoxide in dichloromethane. Sodium 1-naphtholate was prepared from 1-naphthol and sodium methanolate, which subsequently allowed the synthesis of **FM368** with alkyl chloride **5** in acetone instead of dichloromethane to reduce unwanted C-alkylation. The phenolates required for the preparation of **FM352** and **FM358** each had to be synthesized in two steps. 4-Aminophenol (**6**) was reacted with thiophene-2-carbonyl chloride and pyridine to form amide **7**, which was then transformed with sodium methanolate to the corresponding phenolate, finally yielding **FM352** with alkyl chloride **5** in dichloromethane. In a HATU-mediated amidation of 4-aminophenol (**6**) and 1-methyl-1*H*-pyrazole-4-carboxylic acid, product **8** was obtained. Notably, undesired side reactions involving the formation of ester and the amide-ester side products were also generated due to similar reactivities of both the aromatic amine and phenol groups of 4-aminophenol (**6**) towards 1-methyl-1*H*-pyrazole-4-carboxylic acid. Fortunately, the desired amide **8** could be isolated in a reasonable yield. The corresponding phenolate was then synthesized upon treatment with sodium methanolate, which was finally subjected to a Williamson ether synthesis with alkyl chloride **5** in dichloromethane yielding **FM358**.

### 2.3. Biological Evaluation of First-Generation Hybrid Candidates, Assessment of the Concept Strategy, and Refinement and Approach to Second-Generation Hybrid Candidates

The determination of the inhibitory potency and corresponding subtype selectivity of the synthesized hybrid candidates was performed by Reaction Biology Cooperation (Malvern, PA, USA) using a fluorescence-based assay (Table 1).

**Figure 4:**
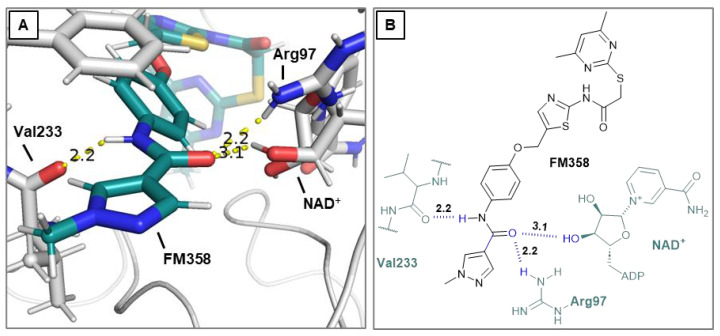
(**A**) Sirt2 docking pose of **FM358** (green) and predicted intermolecular polar contacts (distances in Ångström). Calculation based on PDB ID: 5YQO. The position of cofactor NAD^+^ (grey) was copied from PDB ID: 4RMG for visualization, as not included in the co-crystal structure of **Yang_24a** (PDB ID: 5YQO). (**B**) Comprehensive structural formula representation of the calculated docking pose of **FM358** and Sirt2 with the relevant surrounding amino acids Val233 and Arg97, as well as the corresponding predicted intermolecular polar contacts (distances in Ångström).

Due to different assay conditions used in the original publications, corresponding lead compounds were resynthesized according to literature (**Yang_28e** [17] and **Yang_24a** [19]) or acquired from commercial suppliers **(SirReal2**, Sigma-Aldrich) and included in the screening performed in order to ensure comparability of the values. **SirReal2** was published with an IC_50_ value of 0.4 µM on Sirt2 and achieved a comparable IC_50_ value of 235 nM in the assay system used. The more potent optimizations based on **SirReal2**, **Yang_28e** (published IC_50_ = 41 nM, here IC_50_ = 87 nM) and **Yang_24a** (published IC_50_ = 0.815 µM, here 79 nM), represent a significant improvement of **SirReal2**, as reported. Of the four first target compounds synthesized, **FM358** (IC_50_ = 66 nM) shows the strongest inhibitory activity on Sirt2 and thus outperforms the corresponding lead compounds **SirReal2** (IC_50_ = 235 nM) and **Yang_24a** (IC_50_ = 79 nM). **FM352** (IC_50_ = 94 nM) is more potent than **SirReal2**, however exhibits a slightly lower inhibitory effect than lead compound **Yang_24a**. Hybrids **FM345** (IC_50_ = 5.3 µM) and **FM368** (IC_50_ = 6.5 µM) have clearly lost inhibitory activity compared to the corresponding lead compounds and the replacement of the methylene bridge with the more flexible oxomethylene bridge results in a weakened interaction within the binding pocket of Sirt2. It is possible that the elongation associated with the novel ether units leads to an unfavourable positioning of the benzene ring of **FM345** or the naphthalene ring of **FM368** and to an excessive space requirement. The superiority of the 1-methyl-*N*-phenyl-1*H*-pyrazole-4-carboxamide moiety compared to the *N*-phenylthiophene-2-carboxamide moiety of the lead compounds **Yang_24a** and **Yang_28e**, previously observed by Yang et al. [17] was confirmed, which can be directly transferred to the hybrid compounds **FM358** and **FM352**. Compound **FM358** benefits from the replacement of the benzene ring by the aminothiazole ring, thus the associated rearrangement leads to advantageous interactions, resulting in a 1.2 times stronger inhibition compared to lead compound **Yang_24a** and providing an excellent starting point for further modifications and optimization. Visualization of polar contacts of the docking pose of **FM358** within the binding pocket of Sirt2 reveals various interactions with the substrate channel residues, including hydrogen bonds of the amide-carbonyl with Arg97 and potentially with the ribose unit of cofactor NAD^+^, as well as amide-nitrogen with the backbone carbonyl of Val233 (Figure 4).

### 2.4. Second Generation Targeted Halogenation Approach as a Follow-Up Strategy for Further Improvement of the Hybrid Candidate FM358

**Figure 5:**
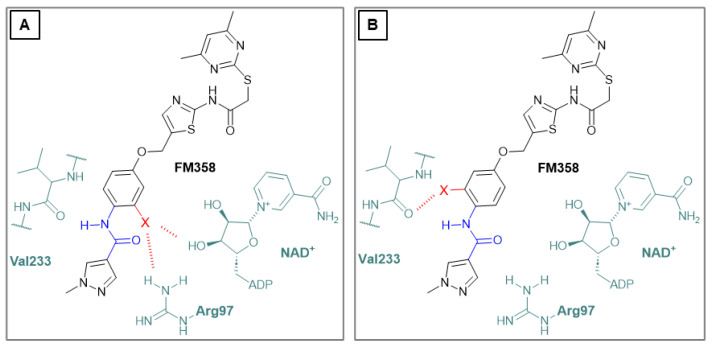
Schematic illustration of two rotameric forms of **FM358** in the Sirt2 binding pocket with the key amino acids identified in the previous docking study (Figure 4). Intended further structural optimization of **FM358** through the introduction of halogen atoms (residues X) to enable new potential electrostatic interactions with binding pocket and cofactor NAD^+^. Abbreviations: X (halogen atom, Cl, Br or I). (**A**) Possible interactions of halogen atoms with NAD^+^ and Arg97. (**B**) Possible interactions of the halogen atoms with Val233.

Concluding that the electrostatic interactions arising from the amide group (Figure 4) are significantly responsible for the superiority of **FM358** and **Yang_24a** over **FM345**, **FM368** and **SirReal2**, a further approach was applied that aimed to expand and intensify electrostatic interactions by targeted halogen substitution [21] (chlorine, bromine, iodine) of the neighboring benzene ring, close to the amide residue of the substrate channel residue with Val233 or Arg97 and NAD^+^ (Figure 5).

### 2.5. Synthesis of Second-Generation Hybrid Candidates

**Scheme 3:**
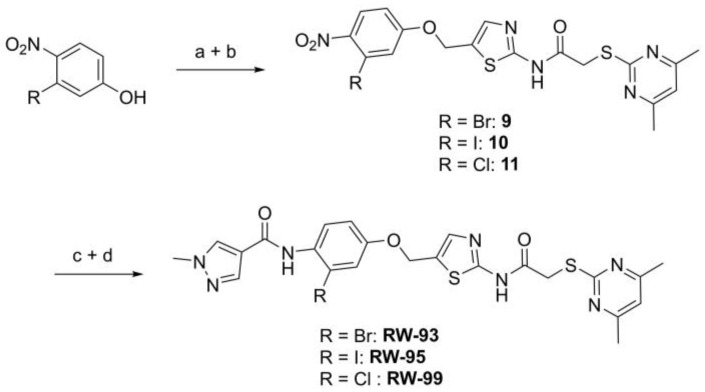
(a) NaOMe 25% (*w*/*w*), DCM, rt, 2 min, crude, (b) **5**, DCM, rt, 3 h, 37% for **9**, 39% for **10**, 48% for **11**; (c) Fe, NH_4_Cl, EtOH/H_2_O (8:1 *v*/*v*), 90 °C, 2 h, crude; (d) 1-methyl-1*H*-pyrazole-4-carbonyl chloride, pyridine, DCM, rt, 1 h, 37% for **RW-93** (over two steps), 39% for **RW-95** (over two steps), 38% for **RW-99** (over two steps).

Amide couplings of *ortho*-halogenated hydroxyanilines with 1-methyl-1*H*-pyrazole-4-carboxylic acid showed initial difficulties with formation of the undesired phenol esters being preferred. Thus, the established synthetic route was slightly modified, and Williamson ether synthesis of the appropriate halogenated phenolates with the central chloromethylthiazole intermediate **5** (Scheme 2) was performed prior to the amide coupling. This circumvents the problem without any additional use of protecting groups for the phenol in the amide coupling (Scheme 3).

The Williamson ether synthesis was performed with initially prepared 3-halogen-4-nitrophenolates (obtained from corresponding phenols using sodium methoxide) and alkyl chloride **5**, based on the previously established general synthesis (Scheme 2). After reduction of the nitro group with Fe/NH_4_Cl, the respective aniline was reacted without further purification with the 1-methyl-1*H*-pyrazole-4-carbonyl chloride, which was previously prepared from the corresponding carboxylic acid by thionyl chloride, to yield the target compounds **RW-93**, **RW-95** and **RW-99**.

### 2.6. Biological Evaluation of Second-Generation Hybrid Candidates and Identification of the Bromine-Substituted Analog RW-93 as Top Inhibitor

The fluorescence-based assays to determine the inhibitory activity on Sirt2 and subtype selectivity of the halogenated derivatives of **FM358** (IC_50_ = 66 nM) revealed satisfactory results and confirmed the universal potency-enhancing impact of the halogen substituted chlorine (**RW-99**), bromine (**RW-93**) and iodine (**RW-95**) derivatives towards Sirt2 (see Table 2). Bromo compound **RW-93** (IC_50_ = 16 nM) shows an impressive 5-fold higher inhibitory activity than the original lead compounds **Yang_24a** (IC_50_ = 79 nM, Sirt2) and is more than 4 times more potent than the top hybrid precursor **FM358**.

**RW-99** (IC_50_ = 25 nM) and **RW-95** (IC_50_ = 49 nM) also demonstrate an improved inhibitory effect compared to **FM358**. Considering the pronounced inhibitory effect of the tested inhibitors, the inhibitory effect on other sirtuin subtypes (Sirt1, Sirt3 and Sirt5) is negligible. The halogen analogues can be ranked in descending order according to their determined inhibitory effect on Sirt2 as Br > Cl > I. An obvious explanation is that the extent of the polarizability and thus the strength of the possible halogen bridge correlates with the atom size and the associated space requirement. Bromine may have a sweet spot in terms of atomic radius and bond strength.

**Figure 6:**
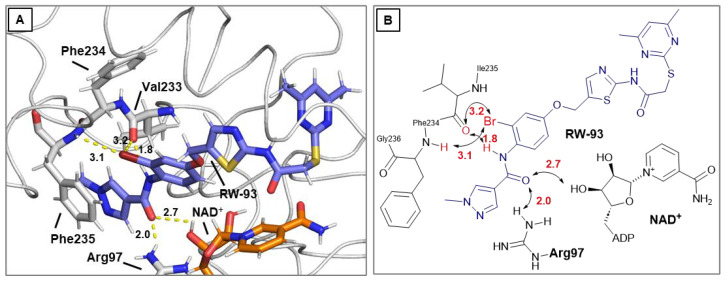
(**A**) Docking pose of **RW-93** (blue). Calculation based on PDB ID: 5YQO, with Sirt2. The position of NAD^+^ (grey) was copied from PDB ID: 4RMG for visualization as not included in the crystal structure of **Yang_24a** (PDB ID: 5YQO). Polar contacts shown as dashed lines, distances in Ångström. (**B**) Schematic 2D illustration of **RW-93** and corresponding polar interaction with the key amino acids of the Sirt2 binding pocket. Non-scaled polar contacts represented with arrows, distances in Ångström.

In the search for the exact cause of the potency-enhancing effect of the halogen substitution and to confirm a suspected halogen bridge, the bromo-substituted top compound **RW-93** was investigated in a further docking study (Figure 6). In principle, **FM358** and **RW-93** show a nearly congruent binding mode in the docking experiment, the only decisive difference being the bromine substitution. Interestingly, according to the predictions, the bromine atom does not orientate itself directly towards the position of NAD^+^ and Arg97 (Figure 5A) but, as also additionally considered, to the side of Val233 and Phe235 (Figure 5B). Originating from the bromine atom, two polar contacts with the backbone carbonyl of Val233 (3.2 Å) and backbone amide-nitrogen of Phe235 (3.1 Å) were predicted, which enable additional intermolecular interactions via halogen bonds. Interactions with Val233 were also described for the lead compounds **SirReal2** and **Yang_24a**, which, according to prediction, were further intensified by respective halogen substitution in the case of **RW-93.** Therefore, this specific binding region of Sirt2 can be considered as a potential attractive interaction target and serve as a key area for the design of further highly potent Sirt2 inhibitors. However, only a co-crystal structure of **RW-93** with Sirt2 can provide reliable information, and intensive efforts are currently being made to uncover the underlying structure-effect relationship, although limited solubility remains a significant barrier to successful co-crystallizations.

### 2.7. Cell Viability Assay

The SirReal-based Sirt2 inhibitors (**FM345**, **FM352**, **FM358**, **FM368**, **RW-93**, **RW-95** and **RW-99**) were tested for potential acute cytotoxicity to human cells in an MTT-based cell viability assay. HL-60 cell line was selected based on literature report that showed that the lead structure **SirReal2** is not toxic in this particular cell line [22]. HL-60 cells were treated with the corresponding Sirt2 inhibitors at various concentrations and then incubated for 24 h. All investigated Sirt2 inhibitors showed no significant acute cytotoxicity with IC_50_ values above 50 µM. Representative cell viability results following treatment with **RW-93** are shown in Figure S1 of the Supplementary Materials.

### 2.8. Free Binding Energy Calculations and Validation Studies

The estimated docking scores and the free binding energies of the lead structures **SirReal2**, **Yang_24a** and **Yang_28e**, and the investigated Sirt2 inhibitors (**FM345**, **FM352**, **FM358**, **FM368**, **RW-93**, **RW-95** and **RW-99**) were calculated based on the PDB ID: 5YQO and compared with their respective IC_50_ values obtained from in vitro experiments. A comparative analysis showed a good degree of correlation between the estimated docking scores as well as the calculated free binding energies and the experimentally determined IC_50_ values with a Pearson correlation coefficient of 0.68 and 0.73, respectively. The most potent Sirt2 inhibitor **RW-93** displayed an estimated docking score of −14.63 kcal/mol and a calculated free binding energy (∆G) of −110.82 kJ/mol (Table 3), which are among the most favourable values of the investigated Sirt2 inhibitors, aligning well with the in vitro determined IC_50_ data.

### 2.9. In Silico ADME Profiling of Sirt2 Hybrid Inhibitors Calculated by QikProp

Selected synthesized Sirt2 hybrid inhibitors were evaluated for their drug-likeness by assessing their physicochemical properties (Table 4). Relevant parameters for the evaluation of the absorption and distribution of the Sirt2 inhibitors such as the predicted octanol/water partition coefficient (QPlogPo/w), brain/blood partition coefficient (QPlogBB) and cell permeability (QPPCaco) showed values within the recommended range. Furthermore, all four Sirt2 hybrid inhibitors showed high predicted oral absorption (HOA) of over 80%. Drug-likeness of these inhibitors was additionally evaluated by their stars value (S) and their conformity to the Lipinski’s rule of five (RO5) with values falling in the recommended range. Notably, these Sirt2 hybrid inhibitors showed poor predicted aqueous solubility with QPlogS values ranging from −8 to −8.5.

## 3. Discussion

By successfully applying a lead structure-based hybridization strategy, highly potent and selective Sirt2 inhibitors were specifically developed, which provide a valuable extension of knowledge of structure–activity relationships of SirReal-type inhibitors. The development of a synthetic route to the previously unknown structural class of 5-(aryloxymethyl)thiazoles led to the identification of **FM358** (IC_50_ = 66 nM), an optimized hybrid compound whose inhibitory potency was further significantly enhanced through targeted ring halogenation. Docking calculations of bromo derivative **RW-93** suggest the formation of halogen bonds to Val233 or Phe235 within the Sirt2 binding pocket, which could contribute significantly to the observed increase in potency and thus could be an attractive target area for potential future design strategy of new Sirt2 inhibitors. With the bromo-substituted **RW-93** (IC_50_ = 16 nM) a highly potent and selective Sirt2 inhibitor was developed, which outperforms original lead compound **Yang_24a** (IC_50_ = 79 nM) by a factor of 5 and lead compound **SirReal2** by a factor of 15 and displays more than 3000-fold selectivity towards related subtypes Sirt1, Sirt3 and Sirt5. Furthermore, acute toxicity of **RW-93** to human cells was assessed and ruled out via MTT assay. Although the potential utility of **RW-93** at the cellular level remains an important objective, this study was still unable to demonstrate its effects in such biological contexts, primarily due to physicochemical limitations. In particular, solubility issues in various media remain a significant obstacle both in cellular assays and co-crystallization experiments. Within the framework of the in silico ADME investigation of the Sirt2 hybrid inhibitors, we showed that the predicted aqueous solubilities of these compounds were poor, aligning with the biological experimental challenges that we faced. However, the majority of relevant pharmacokinetic parameters reveal drug-likeness properties of these Sirt2 inhibitors, highlighting their potential use in further drug development. Our results showcase a novel and an effective strategy in the optimization and the development of highly potent Sirt2 inhibitors that can easily be transferred into the broader spectrum of SAR studies in other fields of medicinal chemistry. Together with computational validation, this strategy was executed with great success to deliver **RW-93**—the most potent low-molecular subtype selective Sirt2 inhibitor known to date.

## 4. Materials and Methods

### 4.1. Chemistry

Commercially available solvents and reagents were utilized as received without any additional purification. NMR spectra were recorded with an Avance III HD 400 MHz Bruker BioSpin (Bruker Corporation, Billerica, MA, USA) for ^1^H-NMR (400 MHz) and ^13^C-NMR (101 MHz) or an Avance III HD 500 MHz Bruker BioSpin (Bruker Corporation, Billerica, MA, USA) for ^1^H-NMR (500 MHz) and 13C-NMR (126 MHz) MestreNova 14.3.0 (Mestrelab Research S.L., Santiago de Compostela, Spain) was utilized for data analysis and the respective chemical shifts (δ) were referenced to the deuterated solvent peak (DMSO-*d*_6_: δH = 2.50 ppm, δC = 39.52 ppm; CD_2_Cl_2_: δH = 5.32 ppm, δC = 53.84 ppm; CDCl_3_: δH = 7.26 ppm, δC = 77.16 ppm). Coupling constants (J) were reported in Hertz (Hz), and multiplicities were given as: s (singlet), d (doublet), t (triplet), q (quartet), or m (multiplet). While the compounds are numbered according to IUPAC guidelines, the respective hierarchy of NMR signal assignments (denoted with apostrophes) were simplified by following the sequence in which the compounds were synthesized. Thin-layer chromatography (TLC) was employed for reaction monitoring, using 0.2 mm silica gel coated POLYGRAM^®^ SIL G/UV254 polyester plates (Macherey-Nagel, Düren, Germany), and visualized under UV light at 254 nm. Flash column chromatography was carried out using SiO_2_ 60 (0.040–0.063 mm, 230–400 mesh ASTM) from Merk (Darmstadt, Germany). Infrared spectroscopy (IR) was performed on a Perkin Elmer FT-IR BXII/1000 spectrometer (Waltham, USA), paired with a DuraSamp IR II Diamond ATR sensor (Smiths Detection, London, UK). The IR spectra were recorded over the range of 4000 to 650 cm^−1^, and significant absorption bands were noted in cm^−1^. High-resolution mass spectrometry (HR-MS) was conducted using either a Jeol Mstation 700 (Akishima, Japan) or a JMS GCmate II Jeol (Akishima, Japan) for electron impact ionization (EI). Electrospray ionization (ESI) HR-MS was performed using a Thermo Finnigan LTQ (Thermo Fisher Scientific, Waltham, MA, USA). Melting points were determined with a Büchi B-540 melting point meter (Fawil, Switzerland). The purity of compounds was assessed using an HP Agilent 1100 HPLC system (Agilent, Santa Clara, CA, USA) with a Zorbax Eclipse Plus^®^ C18 5 µm (4.6 × 150 mm) column (Agilent, Santa Clara, CA, USA), using acetonitrile/water (method 1), or acetonitrile/phosphate buffer pH = 5 (method 2) as mobile phases.

**General Procedure: Nitrobenzene reduction:** To a stirred solution of the appropriate nitrobenzene derivative (1.0 equivalent) in EtOH with a concentration of 0.010 M were added iron powder (5.0 equivalents) and 0.30 M aq. NH_4_Cl (5.0 equivalents) at 50 °C. The reaction mixture was refluxed at 90 °C for 2 h. Afterwards, the solids were removed by filtration and the filtrate was concentrated in vacuo. The crude product was used for the next step without further purification.

**2-((4,6-Dimethylpyrimidin-2-yl)thio)acetic acid** (**2**)**.** 4,6-Dimethylpyrimidine-2-thiol (**1**) (3.80 g, 27.1 mmol, 1.00 eq) and chloroacetic acid (3.07 g, 32.5 mmol, 1.20 eq) were suspended in acetonitrile (25 mL) at room temperature. After addition of NEt_3_ (15.1 mL, 108 mmol, 4.00 eq), the reaction mixture was stirred for 24 h. After evaporating the solvent, the crude product was purified via flash column chromatography (DCM/MeOH/AcOH 100:1:1) to yield thioether **2** as a light-beige solid (4.33 g, 21.8 mmol, 81%); m.p. 128 °C; IR (ATR) *ṽ*/cm^−1^ 3474, 2942, 2534, 1924, 1716, 1584, 1538, 1309, 1267, 1208, 1172, 984, 862, 791, 661; δ_H_ (400 MHz; (CD_3_)_2_SO) 12.73 (s, 1H, COOH), 6.97 (s, 1H, 5-H), 3.90 (s, 2H, CH_2_), 2.33 (s, 6H, 4-CH_3_, 6-CH_3_). δ_C_ (101 MHz; (CD_3_)_2_SO) 170.2 (COOH), 169.0 (C-2), 167.0 (C-4, C-6), 116.0 (C-5), 32.9 (CH_2_), 23.3 (4-CH_3_, 6-CH_3_); HRMS (ESI): *m*/*z* = [M−H]^−^ calculated for C_8_H_9_N_2_O_2_S^−^: 197.0390; found: 197.0390.

**2-((4,6-Dimethylpyrimidin-2-yl)thio)-*N*-(5-formylthiazol-2-yl)acetamide (3).** Carboxylic acid **2** (1.55 g, 7.80 mmol, 1.00 eq) was dissolved in DMF (5 mL) and added to a previously prepared solution of 2-aminothiazole-5-carbaldehyde (1.00 g, 7.80 mmol, 1.00 eq), DMAP (0.477 g, 3.90 mmol, 0.50 eq) and EDC·HCl (1.83 g, 9.36 mmol, 1.20 eq) in 5 mL DMF. After stirring for 16 h at room temperature, the reaction mixture was diluted with water (400 mL) and extracted with EtOAc (3 × 100 mL). The combined organic layers were dried over sodium sulfate. After evaporating the solvent, the crude product was purified via flash column chromatography (DCM/MeOH 100:1) to yield aldehyde **3** as a pale yellow solid (1.11 g, 3.61 mmol, 46%); m.p. 191–193 °C; IR (ATR) *ṽ*/cm^−1^ 2924, 2137, 1688, 1664, 1586, 1555, 1516, 1427, 1385, 1311, 1268, 1234, 1173, 1123, 977, 872, 819, 733; δ_H_ (400 MHz; (CD_3_)_2_SO) 13.02 (s, 1H, NHCO), 9.96 (s, 1H, CHO), 8.44 (s, 1H, 4′-H), 6.97 (s, 1H, 5-H), 4.18 (s, 2H, CH_2_), 2.28 (s, 6H, 4-CH_3_, 6-CH_3_). δ_C_ (101 MHz; (CD_3_)_2_SO) 184.1 (CHO), 168.7 (C-2), 168.4 (NHCO), 167.1 (C-4, C-6), 164.0 (C-2′), 150.7 (C-4′) 132.1 (C-5′), 116.2 (C-5), 34.4 (CH_2_), 23.2 (4-CH_3_, 6-CH_3_); HRMS (ESI): *m*/*z* = [M−H]^−^ calculated for C_12_H_11_N_4_O_2_S_2_^−^: 307.0329; found: 307.0330.

**2-((4,6-Dimethylpyrimidin-2-yl)thio)-*N*-(5-(hydroxymethyl)thiazol-2-yl)acetamide (4).** To a stirred solution of aldehyde **3** (550 mg, 1.78 mmol) in dry MeOH (20 mL) was added NaBH_4_ (101 mg, 2.68 mmol) at 0 °C. The reaction mixture was stirred at 0 °C for 3 h under N_2_ atmosphere. Water (15 mL) was then added, and the reaction mixture was stirred for another 10 min. The suspension was vacuum filtered, and the obtained solid washed with water (1 × 50 mL) and acetone (1 × 50 mL) to give alcohol **4** as a white solid (553 mg, 1.78 mmol, quantitative); m.p. 250–252 °C; IR (ATR) *ṽ*/cm^−1^ 3284, 2917, 1696, 1588, 1537, 1323, 1273, 1262, 1161, 1037, 971, 843, 834, 788, 717; δ_H_ (400 MHz; (CD_3_)_2_SO) 12.22 (s, 1H, NHCO), 7.28 (d, *J* = 1.0 Hz, 1H, 4′-H), 6.96 (s, 1H, 5-H), 5.35 (t, *J* = 5.7 Hz, 1H, OH), 4.57 (dd, *J* = 5.7, 0.9 Hz, 2H, CH_2_OH), 4.11 (s, 2H, CH_2_S), 2.30 (s, 6H, 4-CH_3_, 6-CH_3_). δ_C_ (101 MHz; (CD_3_)_2_SO) 168.9 (C-2), 167.0 (C-4, C-6), 166.9 (NHCO), 157.5 (C-2′), 134.5 (C-4′) 133.1 (C-5′), 116.1 (C-5), 55.7 (CH_2_OH), 34.1 (CH_2_S), 23.3 (4-CH_3_, 6-CH_3_); HRMS (ESI): *m*/*z* = [M−H]^−^ calculated for C_12_H_13_N_4_O_2_S_2_^−^: 309.0485; found: 309.0486.

***N*-(5-(Chloromethyl)thiazol-2-yl)-2-((4,6-dimethylpyrimidin-2-yl)thio)acetamide (5).** Primary alcohol **4** (358 mg, 1.15 mmol, 1.00 eq) was suspended in anhydrous DCM (15 mL) under nitrogen atmosphere and SOCl_2_ (101 µL, 1.38 mmol, 1.20 eq) was added dropwise, while stirring at room temperature. After 18 h, *n*-hexane (5 mL) was added to the reaction mixture and the precipitate was collected by filtration. Subsequently the product was washed with *n*-hexane/DCM (1:1, 3 × 20 mL) to yield chloromethyl compound **5** as a white solid (378 mg, 1.15 mmol, quantitative yield); m.p. 226–233 °C (decomposition); IR (ATR) *ṽ*/cm^−1^ 3270, 2421, 1720, 1624, 1602, 1582, 1540, 1417, 1370, 1333, 1307, 1268, 1158, 876, 849, 716, 703, 695; δ_H_ (400 MHz; (CD_3_)_2_SO) 12.46 (s, 1H, NHCO), 7.53 (s, 1H, 3′-H), 6.96 (s, 1H, 5-H), 5.03 (s, 2H, CH_2_S), 4.13 (s, 2H, CH_2_Cl), 2.29 (s, 6H, 4-CH_3_, 6-CH_3_). δ_C_ (101 MHz; (CD_3_)_2_SO) 168.8 (C-2), 167.4 (NHCO), 167.0 (C-4, C-6), 159.2 (C-2′), 138.2 (C-3′), 128.0 (C-4′), 116.2 (C-5), 38.7 (CH_2_Cl), 34.1 (CH_2_S), 23.2 (4-CH_3_, 6-CH_3_). HRMS (ESI): *m*/*z* = [M-Cl+H_2_O-2H]^−^ calculated for C_12_H_13_N_4_O_2_S_2_^−^: 309.0485; found: 309.0483.

**2-((4,6-Dimethylpyrimidin-2-yl)thio)-*N*-(5-(phenoxymethyl)thiazol-2-yl)acetamide (FM345).** Chloromethyl compound **5** (154 mg, 0.468 mmol, 1.00 eq) and sodium phenolate (54.4 mg, 0.468 mmol, 1.00 eq) were suspended in anhydrous DCM (20 mL) under nitrogen atmosphere and the reaction mixture was stirred for 24 h at room temperature. Following TLC monitoring, an additional equivalent of sodium phenolate (54.4 mg, 0.468 mmol, 1.00 eq) was added to complete the reaction. After 2 h, the solvent was evaporated under reduced pressure. NaOH solution (aq., 2 M, 50 mL) was added to the residue and the mixture was extracted with EtOAc (4 × 75 mL). The combined organic layers were dried over sodium sulfate. After evaporating the solvent, the crude product was purified by flash column chromatography (DCM/MeOH 100:1) to yield **FM345** as a white solid (33.0 mg, 0.0854 mmol, 18%); m.p. 160–163 °C; IR (ATR) *ṽ*/cm^−1^ 2916, 2159, 1688, 1583, 1539, 1496, 1384, 1312, 1270, 1238, 1171, 1139, 1008, 966, 883, 803, 749, 733, 687; δ_H_ (500 MHz; (CD_3_)_2_SO) 12.38 (s, 1H, NHCO), 7.54 (s, 1H, 4′-H), 7.33–7.25 (m, 2H, 3″-H, 5″-H), 7.03–6.98 (m, 2H, 2″-H, 5″-H), 6.98–6.91 (m, 2H, 4″-H, 5-H), 5.25 (s, 2H, CH_2_O), 4.11 (s, 2H, CH_2_S), 2.28 (s, 6H, 4-CH_3_, 6-CH_3_). δ_C_ (126 MHz; (CD_3_)_2_SO) 168.9 (C-2), 167.2 (NHCO), 167.0 (C-4, C-6), 158.6 (C-2′), 157.7 (C-1″), 137.7 (C-4′), 129.5 (C-3″, C-5″), 126.6 (C-5′), 121.0 (C-4′), 116.1 (C-5), 115.0 (C-2″, C-6″), 61.8 (CH_2_O), 34.1 (CH_2_S), 23.2 (4-CH_3_, 6-CH_3_). HRMS (ESI): *m*/*z* = [M+H]^+^ calculated for C_18_H_19_N_4_O_2_S_2_^+^: 387.0944; found: 387.0944; Purity (HPLC): >99% (210 nm), >99% (254 nm), (method 1).

***N*-(4-Hydroxyphenyl)thiophene-2-carboxamide (7).** At room temperature, 4-aminophenol (**6**) (9.38 g, 86.0 mmol, 1.00 eq) was dissolved in DMF (30 mL) and pyridine (7.41 mL, 91.7 mmol, 1.07 eq) was added. The stirred mixture was cooled to 0 °C, and 2-thiophenecarbonyl chloride (9.78 mL, 91.4 mmol, 1.06 eq) was added dropwise. After 1.5 h the reaction mixture was diluted with water (300 mL) and extracted with EtOAc (4 × 100 mL). The combined organic layers were dried over sodium sulfate and concentrated in vacuo to obtain a viscous oil. The addition of DCM/hexanes (5 mL, 9:1) to the viscous oil induced the crystallization process. The obtained crystals were collected by filtration and washed with DCM/hexanes (9:1, 3 × 50 mL) to yield amide **7** as a white-pink solid (13.0 g, 59.1 mmol, 69%); m.p. 192–195 °C; IR (ATR) *ṽ*/cm^−1^ 3109, 1620, 1598, 1539, 1505, 1438, 1355, 1314, 1264, 1245, 1221, 1173, 1095, 884, 8299, 765, 739, 712. δ_H_ (400 MHz; (CD_3_)_2_SO) 10.01 (s, 1H, NHCO), 9.27 (s, 1H, OH), 7.96 (dd, *J* = 3.8, 1.1 Hz, 1H, 3′-H), 7.81 (dd, *J* = 5.0, 1.2 Hz, 1H, 5′-H), 7.50–7.44 (m, 2H, 2-H, 6-H), 7.20 (dd, *J* = 5.0, 3.7 Hz, 1H, 4′-H), 6.77–6.72 (m, 2H, 3-H, 5-H). δ_C_ (101 MHz; (CD_3_)_2_SO) 159.4 (NHCO), 153.8 (C-4), 140.4 (C-2′), 131.3 (C-5′), 130.1 (C-1′), 128.5 (C-3′), 128.0 (C-4′), 122.4 (C-2, C-6), 115.0 (C-3, C-5). HRMS (ESI): *m*/*z* = [M−H]^−^ calculated for C_11_H_8_NO_2_S^−^: 218.0281; found: 218.0280.

***N*-(4-Hydroxyphenyl)-1-methyl-1*H*-pyrazole-4-carboxamide (8).** DIPEA (3.80 mL, 22.0 mmol, 3.00 eq) and HATU (2.26 g, 5.95 mmol, 1.50 eq) were added to a solution of 1-methyl-1*H*-pyrazole-4-carboxylic acid (925 mg, 7.33 mmol, 1.00 eq) in anhydrous THF (15 mL) and the reaction mixture was stirred at room temperature for 1 h. 4-Aminophenol (**6**) (800 mg, 7.33 mmol, 1.00 eq) was added and the reaction mixture was stirred for another 3 h. Then the mixture was diluted with water (150 mL) and extracted with DCM (4 × 100 mL). The combined organic layers were dried over sodium sulfate. After evaporating the solvent, the crude product was purified by flash column chromatography (hexanes/EtOAc 2:8) to yield amide **8** as a white solid (171 mg, 0.787 mmol, 11%); m.p. 227–230 °C; IR (ATR) *ṽ*/cm^−1^ 3347, 2926, 1641, 1601, 1558, 1509, 1430, 1388, 1308, 1273, 1201, 1151, 1099, 1005, 841, 812, 749, 703. δ_H_ (400 MHz; (CD_3_)_2_SO) 9.57 (s, 1H, NHCO), 9.18 (s, 1H, OH), 8.24 (s, 1H, 5′-H), 7.96 (s, 1H, 3′-H), 7.48–7.40 (m, 2H, 2-H, 6-H), 6.75–6.67 (m, 2H, 3-H, 5-H), 3.87 (s, 3H, CH_3_). δ_C_ (101 MHz; (CD_3_)_2_SO) 160.0 (NHCO), 153.4 (C-4), 138.6 (C-3′), 132.3 (C-5′), 130.6 (C-1), 121.9 (C-2, C-6), 118.7 (C-4′), 114.0 (C-3, C-5), 38.8 (CH_3_). HRMS (ESI): *m*/*z* = [M+H]^+^ calculated for C_11_H_12_N_3_O_2_^+^: 218.0924; found: 218.0928.

***N*-(4-((2-(2-((4,6-Dimethylpyrimidin-2-yl)thio)acetamido)thiazol-5-yl)methoxy)phenyl)thiophene-2-carboxamide (FM352).** Under nitrogen atmosphere, phenol **7** (268 mg, 1.22 mmol, 1.00 eq) was dissolved in anhydrous methanol (2 mL) and sodium methanolate (25% (*w*/*w*) in MeOH, 272 µL, 1.22 mmol, 1.00 eq) was added at room temperature. The reaction mixture was stirred for 2 min, subsequently the solvent was evaporated. The residue was dried under high vacuum to yield sodium 4-(thiophene-2-carboxamido)phenolate in quantitative yield, which was used without further purification for the next step. Chloromethyl compound **5** (123 mg, 0.374 mmol, 1.00 eq) and the previously prepared sodium 4-(thiophene-2-carboxamido)phenolate (90.2 mg, 0.374 mmol, 1.00 eq) were suspended in anhydrous DCM (15 mL) and the reaction mixture was stirred for 18 h at room temperature. Following TLC monitoring, an additional equivalent of sodium 4-(thiophene-2-carboxamido)phenolate (90.2 mg, 0.374 mmol, 1.00 eq) was added to complete the reaction. After 2 h, NaOH solution (aq., 2 M, 50 mL) was added, and the mixture was extracted with DCM (3 × 50 mL). The combined organic layers were dried over sodium sulfate. After evaporating the solvent, the crude product was purified by flash column chromatography (DCM/MeOH 100:1) to yield **FM352** as a white solid (31.5 mg, 0.0616 mmol, 17%); m.p. 176–179 °C; IR (ATR) *ṽ*/cm^−1^ 2923, 2164, 1700, 1635, 1578, 1512, 1422, 1323, 1265, 1229, 1159, 1010, 845, 734; δ_H_ (500 MHz; (CD_3_)_2_SO) 12.38 (s, 1H, 2′-NHCO), 10.11 (s, 1H, 4″-NHCO), 7.97 (d, *J* = 3.7 Hz, 1H, 3‴-H), 7.83 (d, *J* = 4.9 Hz, 1H, 5‴-H), 7.63–7.57 (m, 2H, 3″-H, 5″-H), 7.55 (s, 1H, 4′-H), 7.21 (t, *J* = 4.3 Hz, 1H, 4‴-H), 7.03–6.98 (m, 2H, 2″-H, 6″-H), 6.95 (s, 1H, 5-H), 5.25 (s, 2H, CH_2_O), 4.12 (s, 2H, CH_2_S), 2.28 (s, 6H, 4-CH_3_, 6-CH_3_). δ_C_ (126 MHz; (CD_3_)_2_SO) 168.9 (C-2), 167.2 (2′-NHCO), 167.0 (C-4, C-6), 159.6 (4″-NHCO), 158.6 (C-2′), 154.0 (C-1″), 140.2 (C-2‴), 137.8 (C-4′), 132.2 (C-4″), 131.5 (C-5‴), 128.8 (C-3‴), 128.0 (C-4‴), 126.6 (C-5′), 121.9 (C-3″, C-5″), 116.1 (C-5), 115.1 (C-2″, C-6″), 62.1 (CH_2_O), 34.1 (CH_2_S), 23.2 (4-CH_3_, 6-CH_3_). HRMS (ESI): *m*/*z* = [M−H]^−^ calculated for C_23_H_20_N_5_O_3_S_3_^−^: 510.0734; found: 510.0733. Purity (HPLC): >99% (210 nm), >99% (254 nm), (method 1).

***N*-(4-((2-(2-((4,6-Dimethylpyrimidin-2-yl)thio)acetamido)thiazol-5-yl)methoxy)-phenyl)-1-methyl-1*H*-pyrazole-4-carboxamide (FM358).** Under nitrogen atmosphere, phenol 8 (294 mg, 1.35 mmol, 1.00 eq) was dissolved in anhydrous methanol (2 mL) and sodium methanolate (25% (*w*/*w*) in MeOH, 302 µL, 1.35 mmol, 1.00 eq) was added at room temperature. The reaction mixture was stirred for 2 min, subsequently the solvent was evaporated. The residue was dried under high vacuum to yield sodium 4-(1-methyl-1*H*-pyrazole-4-carboxamido)phenolate in quantitative yield, which was used without further purification for the next step. Under nitrogen atmosphere, chloromethyl compound 5 (150 mg, 0.456 mmol, 1.00 eq) and the previously prepared sodium 4-(1-methyl-1*H*-pyrazole-4-carboxamido)phenolate (109 mg, 0.456 mmol, 1.00 eq) were suspended in anhydrous DCM (15 mL) and the reaction mixture was stirred for 18 h at room temperature. Following TLC monitoring, an additional equivalent of sodium 4-(1-methyl-1*H*-pyrazole-4-carboxamido)phenolate (0.456 mmol, 109 mg, 1.00 eq) was added to complete the reaction. After 2 h, MeOH (1 mL) was added, and the reaction mixture was concentrated in vacuo. The crude product was purified by flash column chromatography (DCM/MeOH/AcOH 100:3:0.5) to yield FM358 as a white solid (87.1 mg, 0.171 mmol, 38%); m.p. 188–191 °C; IR (ATR) *ṽ*/cm^−1^ 3285, 2923, 1692, 1640, 1578, 1559, 1512, 1489, 1438, 1413, 1378, 1325, 1268, 1226, 1172, 1158, 1007, 977, 870, 845, 820, 779, 757, 717; δ_H_ (500 MHz; (CD_3_)_2_SO) 12.37 (s, 1H, 2′-NHCO), 9.68 (s, 1H, 4″-NHCO), 8.26 (s, 1H, 4‴-H), 7.97 (s, 1H, 2‴-H), 7.60–7.58 (m, 2H, 3″-H, 5″-H), 7.53 (s, 1H, 4′-H), 6.98 (m, 2H, 2″-H), 6.95 (s, 1H, 5-H) 5.23 (s, 2H, CH_2_O), 4.11 (s, 2H, CH_2_S), 3.88 (s, 3H, NCH_3_), 2.28 (s, 6H, 4-CH_3_, 6-CH_3_). δ_C_ (126 MHz; (CD_3_)_2_SO) 168.9 (C-2), 167.2 (2’-NHCO), 167.0 (C-4, C-6), 160.1 (4″-NHCO), 158.8 (C-2’), 153.6 (C-1″), 138.7 (C-2‴), 137.7 (C-4’), 132.7 (C-4″), 132.5 (C-4‴), 126.6 (C-5’), 121.4 (C-3″, C-5″), 118.6 (C-3‴), 116.1 (C-5‴), 115.1 (C-2‴, C-6‴), 62.2 (CH_2_O), 38.8 (NCH_3_), 34.2 (CH_2_S), 23.2 (4-CH_3_, 6-CH_3_); HRMS (ESI): *m*/*z* = [M−H]^−^ calculated for C_23_H_22_N_7_O_3_S_2_^−^: 508.1231; found: 508.1231; Purity (HPLC): >99% (210 nm), >99% (254 nm), (method 1).

**2-((4,6-Dimethylpyrimidin-2-yl)thio)-*N*-(5-((naphthalen-1-yloxy)methyl)thiazol-2-yl)acetamide (FM368).** Under nitrogen atmosphere, naphthalen-1-ol (377 mg, 2.61 mmol, 1.00 eq) was dissolved in anhydrous methanol (2 mL) and sodium methanolate (25% (*w*/*w*) in MeOH, 583 µL, 2.61 mmol, 1.00 eq) was added at room temperature. The reaction mixture was stirred for 2 min, subsequently the solvent was evaporated. The residue was dried under high vacuum to yield sodium 1-naphtholate in quantitative yield, which was used without further purification for the next step. Under nitrogen atmosphere chloromethyl compound **5** (150 mg, 0.456 mmol, 1.00 eq) and the previously prepared sodium 1-naphtholate (75.8 mg, 0.456 mmol, 1.00 eq) were suspended in anhydrous acetone (5 mL) and the reaction mixture was stirred for 3 h at room temperature. Following TLC monitoring, an additional equivalent of sodium 1-naptholate (75.8 mg, 0.456 mmol, 1.00 eq) was added to complete the reaction. After 2 h, water (50 mL) was added to the reaction mixture and the mixture was extracted with EtOAc (3 × 100 mL). The combined organic layers were dried over sodium sulfate. After evaporating the solvent in vacuo, the crude product was purified by flash column chromatography (DCM/MeOH 100:0.5) to yield **FM368** as a white solid (28.0 mg, 0.0641 mmol, 14%); m.p. 181–183 °C; IR (ATR) *ṽ*/cm^−1^ 2907, 1697, 1580, 1552, 1506, 1437, 1396, 1367, 1320, 1264, 1241, 1163, 1099, 1065, 1018, 975, 891, 874, 859, 834, 778, 768, 717; δ_H_ (500 MHz; (CD_3_)_2_SO) 12.42 (s, 1H; NHCO), 8.09 (d, *J* = 8.1 Hz, 1H, 8″-H), 7.87 (d, *J* = 8.0 Hz, 1H, 5″-H), 7.63 (s, 1H, 4’-H), 7.55–7.40 (m, 4H, 3″-H, 4″-H, 6″-H, 7″-H), 7.14 (d, *J* = 7.6 Hz, 1H, 2″-H), 6.95 (s, 1H, 5-H), 5.47 (s, 2H, CH_2_O), 4.13 (s, 2H, CH_2_S), 2.29 (s, 6H, 4-CH_3_, 6-CH_3_). δ_C_ (126 MHz; (CD_3_)_2_SO) 168.9 (C-2), 167.2 (NHCO), 167.0 (C-4, C-6), 158.7 (C-2’), 153.1 (C-1″), 137.6 (C-4’), 134.1 (C-4a″), 127.5 (C-5″), 126.7 (C-5’), 126.5 (C-3″), 126.1 (C-6″), 125.5 (C-7″), 125.0 (C-8a″), 121.4 (C-8″), 120.5 (C-4″), 116.1 (C-5), 106.1 (C-2″), 62.6 (CH_2_O), 34.1 (CH_2_S), 23.2 (4-CH_3,_ 6-CH_3_); HRMS (EI): *m*/*z* = [M]^•+^ calculated for C_22_H_20_N_4_O_2_S_2_^•+^: 436.1028; found: 436.1032; Purity (HPLC): >99% (210 nm), >99% (254 nm), (method 2).

***N*-(5-((3-Bromo-4-nitrophenoxy)methyl)thiazol-2-yl)-2-((4,6-dimethylpyrimidin-2-yl)thio)acetamide (9).** To a stirred solution of 3-bromo-4-nitrophenol (150 mg, 0.667 mmol) in dry MeOH (3 mL) was added sodium methanolate 25% (*w*/*w*) in MeOH (0.153 mL, 0.667 mmol). The solution was stirred at room temperature for 5 min, then concentrated in vacuo to give sodium 3-bromo-4-nitrophenolate as an orange solid, which was used for the next step without further purification. Chloromethyl compound **5** (80.0 mg, 0.243 mmol) and sodium 3-bromo-4-nitrophenolate (158 mg, 0.730 mmol) were dissolved in dry DCM (7 mL) and stirred at room temperature for 3 h under N_2_ atmosphere. The reaction mixture was then diluted with DCM (50 mL) and washed with 2M NaOH (3 × 50 mL). The organic phase was then dried using a phase separation paper, concentrated in vacuo and purified by flash column chromatography (DCM/MeOH 98:2) to give ether **9** as a pale-yellow solid (45.9 mg, 0.0899 mmol, 37%); m.p. 175 °C; IR (ATR) *ṽ*/cm^−1^ 2923, 1742, 1666, 1578, 1555, 1518, 1477, 1432, 1372, 1340, 1318, 1267, 1217, 1170, 1153, 1137, 1000, 979, 965, 809, 746, 721; δ_H_ (400 MHz; (CD_3_)_2_SO) 12.44 (s, 1H, CONH), 8.06 (d, *J* = 9.1 Hz, 1H, 5″-H), 7.61 (s, 1H, 4’-H), 7.55 (d, *J* = 2.6 Hz, 1H, 2″-H), 7.23 (dd, *J* = 9.1, 2.7 Hz, 1H, 6″-H), 6.95 (d, *J* = 0.6 Hz, 1H, 5-H), 5.46–5.43 (m, 2H, OCH_2_), 4.12 (s, 2H, SCH_2_), 2.28 (s, 6H, 4-CH_3_ and 6-CH_3_). δ_C_ (126 MHz; (CD_3_)_2_SO) 168.9 (C-2), 167.3 (CONH), 167.0 (C-4 and C-6), 161.0 (C-1″), 159.1 (C-2’), 142.6 (C-4″), 138.8 (C-4’), 127.9 (C-5″), 125.1 (C-5’), 120.6 (C-2″), 116.1 (C-5), 115.5 (C-3″), 115.2 (C-6″), 63.1 (OCH_2_), 34.1 (SCH_2_), 23.2 (4-CH_3_ and 6-CH_3_). HRMS (ESI): *m*/*z* = [M+H]^+^ calculated for C_18_H_17_BrN_5_O_4_S_2_^+^: 509.9905; found: 509.9895.

**2-((4,6-Dimethylpyrimidin-2-yl)thio)-*N*-(5-((3-iodo-4-nitrophenoxy)methyl)thiazol-2-yl)acetamide (10).** To a stirred solution of 3-iodo-4-nitrophenol (220 mg, 0.805 mmol) in dry MeOH (3 mL) was added sodium methanolate 25% (*w*/*w*) in MeOH (0.179 mL, 0.805 mmol). The solution was stirred at room temperature for 5 min, then concentrated in vacuo to give sodium 3-iodo-4-nitrophenolate as an orange solid, which was used for the next step without further purification. Chloromethyl compound **5** (143 mg, 0.434 mmol) and sodium 3-iodo-4-nitrophenolate (177 mg, 0.650 mmol) were dissolved in dry DCM (7 mL) and stirred at room temperature for 3 h under N_2_ atmosphere. The reaction mixture was then diluted with DCM (50 mL) and washed with 2M NaOH (3 × 50 mL). The organic phase was then dried using a phase separation paper, concentrated in vacuo and purified by flash column chromatography (DCM/MeOH 98:2) to give ether **10** as a pale-yellowsolid (93.3 mg, 0.167 mmol, 39%); m.p. 81 °C; IR (ATR) *ṽ*/cm^−1^ 2920, 1688, 1574, 1515, 1337, 1315, 1265, 1222, 1165, 988, 868, 815, 747; δ_H_ (400 MHz; (CD_3_)_2_SO) 12.43 (s, 1H, CONH), 7.99 (d, *J* = 9.0 Hz, 1H, 5′-H), 7.72 (d, *J* = 2.7 Hz, 1H, 2′-H), 7.60 (s, 1H, 4-H), 7.23 (dd, *J* = 9.1, 2.7 Hz, 1H, 6′-H), 6.95 (s, 1H, 5″-H), 5.43–5.42 (m, 2H, OCH_2_), 4.12 (s, 2H, SCH_2_), 2.28 (s, 6H, 4″-CH_3_ and 6″-CH_3_). δ_C_ (101 MHz; (CD_3_)_2_SO) 168.9 (C-2″), 167.3 (CONH), 167.0 (C-4″ and C-6″), 160.6 (C-1′), 159.1 (C-2), 146.0 (C-4′), 138.7 (C-4), 127.3 (C-2′ or C-5′), 127.1 (C-2′ or C-5′), 125.2 (C-5), 116.1 (C-5″), 115.5 (C-6′), 90.2 (C-3′), 62.9 (OCH_2_), 34.1 (SCH_2_), 23.2 (4″-CH_3_ and 6″-CH_3_); HRMS (ESI): *m*/*z* = [M+H]^+^ calculated for C_18_H_17_IN_5_O_4_S_2_^+^: 557.9761; found: 557.9755.

***N*-(5-((3-Chloro-4-nitrophenoxy)methyl)thiazol-2-yl)-2-((4,6-dimethylpyrimidin-2-yl)thio)acetamide (11).** To a stirred solution of 3-chloro-4-nitrophenol (141 mg, 0.805 mmol) in dry MeOH (3 mL) was added sodium methanolate 25% (*w*/*w*) in MeOH (0.179 mL, 0.805 mmol). The solution was stirred at room temperature for 5 min, then concentrated in vacuo to give sodium 3-chloro-4-nitrophenolate as an orange solid, which was used for the next step without further purification. Chloromethyl compound **5** (143 mg, 0.434 mmol) and sodium 3-chloro-4-nitrophenolate (116 mg, 0.652 mmol) were dissolved in dry DCM (7 mL) and stirred at room temperature for 3 h under N_2_ atmosphere. The reaction mixture was then diluted with DCM (50 mL) and washed with 2M NaOH (3 × 50 mL). The organic phase was then dried using a phase separation paper, concentrated in vacuo and purified by flash column chromatography (DCM/MeOH 98:2) to give ether **11** as a pale-yellow solid (96.6 mg, 0.207 mmol, 48%); m.p. 225 °C; IR (ATR) *ṽ*/cm^−1^ 2924, 1666, 1583, 1556, 1516, 1481, 1433, 1373, 1640, 1321, 1267, 1219, 1171, 1155, 1138, 1037, 985, 964, 891, 825, 808, 747, 721; δ_H_ (400 MHz; (CD_3_)_2_SO) 12.44 (s, 1H, CONH), 8.10 (d, *J* = 9.1 Hz, 1H, 5″-H), 7.62 (s, 1H, 4‘-H), 7.43 (d, *J* = 2.7 Hz, 1H, 2″-H), 7.20 (dd, *J* = 9.2, 2.7 Hz, 1H, 6″-H), 6.95 (s, 1H, 5-H), 5.45 (d, *J* = 0.7 Hz, 2H, OCH_2_), 4.12 (s, 2H, SCH_2_), 2.28 (s, 6H, 4-CH_3_ and 6-CH_3_). δ_C_ (101 MHz; (CD_3_)_2_SO) 168.9 (C-2), 167.3 (CONH), 167.0 (C-4 and C-6), 161.3 (C-1″), 159.1 (C-2′), 140.6 (C-4″), 138.8 (C-4’), 128.1 (C-5″), 127.7 (C-3″), 125.0 (C-5′), 117.5 (C-2″), 116.1 (C-5), 114.9 (C-6″), 63.2 (OCH_2_), 34.1 (SCH_2_), 23.2 (4-CH_3_ and 6-CH_3_). HRMS (ESI): *m*/*z* = [M+Na]^+^ calculated for C_18_H_16_ClN_5_O_4_S_2_Na^+^: 488.0230; found: 488.0218.

***N*-(2-Bromo-4-((2-(2-((4,6-dimethylpyrimidin-2-yl)thio)acetamido)thiazol-5-yl)-methoxy)phenyl)-1-methyl-1*H*-pyrazole-4-carboxamide (RW-93).** 1-Methyl-1*H*-pyrazole-4-carboxylic acid (3.00 g, 23.8 mmol) was treated with thionyl chloride (10 mL, 136 mmol) and the mixture stirred at 80 °C for 2 h. The excess thionyl chloride was then removed in vacuo to give 1-methyl-1*H*-pyrazole-4-carbonyl chloride as a thick white suspension which solidified to a white solid upon standing at room temperature. Reduction of nitrobenzene 9 (46.0 mg, 90.1 µmol) was performed according to General Procedure: Nitrobenzene reduction. The obtained crude amine was dissolved in dry DCM (1 mL). 1-Methyl-1*H*-pyrazole-4-carbonyl chloride (26.2 mg, 0.181 mmol) and pyridine (3.66 µL, 45.3 µmol) were added into the reaction mixture and the solution was stirred at room temperature for 1 h. The reaction mixture was then concentrated in vacuo and the crude product was purified by flash column chromatography (DCM/MeOH 98:2) to give RW-93 as a white solid (19.7 mg, 33.5 µmol, 37% over two steps); m.p. 185 °C; elemental analysis found: C, 46.9; H, 3.9; N, 16.1; S, 10.9%; calc. for C_23_H_22_BrN_7_O_3_S_2_: C, 46.9; H, 3.8; N, 16.65; S, 10.9%; IR (ATR) *ṽ*/cm^−1^ 3418, 2918, 1689, 1675, 1581, 1532, 1411, 1392, 1314, 1293, 1278, 1265, 1223, 1165, 1124, 1047, 1003, 982, 870, 845, 807, 743; δ_H_ (400 MHz; (CD_3_)_2_SO) 12.39 (s, 1H, 2″-NHCO), 9.49 (s, 1H, 4-CONH), 8.25 (s, 1H, 5-H), 7.96 (s, 1H, 3-H), 7.56 (s, 1H, 4″-H), 7.37–7.33 (m, 2H, 3’-H and 6’-H), 7.05 (dd, *J* = 8.8, 2.8 Hz, 1H, 5’-H), 6.96 (s, 1H, 5‴-H), 5.31 (s, 2H, OCH_2_), 4.12 (s, 2H, SCH_2_), 3.88 (s, 3H, NCH_3_), 2.29 (s, 6H, 4‴-CH_3_ and 6‴-CH_3_). δ_C_ (101 MHz; (CD_3_)_2_SO) 168.9 (C-2‴), 167.0 (2″-NHCO, C-4‴ and C-6‴), 160.7 (4-CONH), 158.9 (C-2″) 156.2 (C-4’), 138.8 (C-3), 138.1 (C-4″), 132.6 (C-5), 129.8 (C-3’), 129.6 (C-1’), 126.0 (C-5″), 118.6 (C-6’), 117.9 (C-4), 116.1 (C-5‴), 115.0 (C-5’), 62.5 (OCH_2_), 38.8 (NCH_3_), 34.2 (SCH_2_), 23.2 (4‴-CH_3_ and 6‴-CH_3_). HRMS (ESI): *m*/*z* = [M+H]^+^ calculated for C_23_H_23_BrN_7_O_3_S_2_^+^: 588.0482; found: 588.0480.

***N*-(4-((2-(2-((4,6-Dimethylpyrimidin-2-yl)thio)acetamido)thiazol-5-yl)methoxy)-2-iodophenyl)-1-methyl-1*H*-pyrazole-4-carboxamide (RW-95).** 1-Methyl-1*H*-pyrazole-4-carboxylic acid (3.00 g, 23.8 mmol) was treated with thionyl chloride (10 mL, 136 mmol) and the mixture stirred at 80 °C for 2 h. The excess thionyl chloride was then removed in vacuo to give 1-methyl-1*H*-pyrazole-4-carbonyl chloride as a thick white suspension which solidified to a white solid upon standing at room temperature. Reduction of nitrobenzene 10 (83.0 mg, 0.149 mmol) was performed according to General Procedure: Nitrobenzene reduction. The obtained crude primary amine was dissolved in dry DCM (1.5 mL). 1-Methyl-1*H*-pyrazole-4-carbonyl chloride (43.0 mg, 0.298 mmol) and pyridine (6.02 µL, 74.4 µmol) were added into the reaction mixture and the solution was stirred at room temperature for 1 h. The reaction mixture was then concentrated in vacuo and the crude product was purified via flash column chromatography (DCM/MeOH 98:2) to give RW-95 as a white solid (37.0 mg, 58.2 µmol, 39% over two steps); m.p. 202 °C; elemental analysis: found: C, 43.3; H, 3.5; N, 15.2; S, 10.1%; calc. for C_23_H_22_IN_7_O_3_S_2_: C, 43.5; H, 3.5; N, 15.4; S, 10.1%; IR (ATR) *ṽ*/cm^−1^ 3396, 3175, 3139, 2918, 1697, 1671, 1580, 1553, 1525, 1433, 1386, 1327, 1309, 1276, 1268, 1217, 1206, 1168, 1151, 991, 984, 855, 815, 777, 745, 732; δ_H_ (500 MHz; (CD_3_)_2_SO) 12.40 (s, 1H, 2″-NHCO), 9.48 (s, 1H, 4-CONH), 8.25 (s, 1H, 5-H), 7.96 (s, 1H, 3-H), 7.56 (s, 1H, 4″-H), 7.53 (d, *J* = 2.8 Hz, 1H, 3’-H), 7.25 (d, *J* = 8.8 Hz, 1H, 6’-H), 7.07 (dd, *J* = 8.7, 2.8 Hz, 1H, 5’-H), 6.96 (s, 1H, 5‴-H), 5.30 (s, 2H, OCH_2_), 4.12 (s, 2H, SCH_2_), 3.88 (s, 3H, NCH_3_), 2.29 (s, 6H, 4‴-CH_3_ and 6‴-CH_3_). δ_C_ (126 MHz; (CD_3_)_2_SO) 168.9 (C-2‴), 167.2 (2″-NHCO), 167.0 (C-4‴ and C-6‴), 160.7 (4-CONH), 158.8 (C-2″), 156.2 (C-4’), 138.8 (C-3), 138.1 (C-4″), 133.0 (C-1’), 132.5 (C-5), 129.1 (C-6’), 126.1 (C-5″), 124.5 (C-3’), 118.1 (C-4), 116.1 (C-5‴), 115.6 (C-5’), 99.6 (C-2’), 62.4 (OCH_2_), 38.8 (NCH_3_), 34.1 (SCH_2_), 23.2 (4‴-CH_3_ and 6‴-CH_3_); HRMS (ESI): *m*/*z* = [M+H]^+^ calculated for C_23_H_23_IN_7_O_3_S_2_^+^: 636.0343; found: 636.0336.

***N*-(2-Chloro-4-((2-(2-((4,6-dimethylpyrimidin-2-yl)thio)acetamido)thiazol-5-yl)methoxy)phenyl)-1-methyl-1*H*-pyrazole-4-carboxamide (RW-99).** 1-Methyl-1*H*-pyrazole-4-carboxylic acid (3.00 g, 23.8 mmol) was treated with thionyl chloride (10 mL, 136 mmol) and the mixture stirred at 80 °C for 2 h. The excess thionyl chloride was then removed in vacuo to give 1-methyl-1*H*-pyrazole-4-carbonyl chloride as a thick white suspension which solidified to a white solid upon standing at room temperature. Reduction of nitrobenzene 9 (80.0 mg, 0.172 mmol) was performed according to General Procedure: Nitrobenzene reduction. The obtained crude amine was dissolved in dry DCM (1.5 mL). 1-methyl-1*H*-pyrazole-4-carbonyl chloride (49.7 mg, 0.344 mmol) and pyridine (6.96 µL, 86.0 µmol) were added into the reaction mixture and the solution was stirred at room temperature for 1 h. The reaction mixture was then concentrated in vacuo and the crude product was purified via flash column chromatography (DCM/MeOH 98:2) to give RW-99 as a white solid (35.8 mg, 65.8 µmol, 38% over two steps); m.p. 195 °C; elemental analysis: found: C, 50.5; H, 4.1; N, 17.6; S, 11.7; Cl, 6.9%; calc: for C_23_H_22_ClN_7_O_3_S_2_: C, 50.8; H, 4.1; N, 18.0; S, 11.8; Cl, 6.5%; IR (ATR) *ṽ*/cm^−1^ 2918, 1679, 1582, 1555, 1535, 1394, 1315, 1295, 1281, 1267, 1224, 1167, 1050, 1003, 900, 869, 845, 809, 743; δ_H_ (400 MHz; (CD_3_)_2_SO) 12.40 (s, 1H, 2″-NHCO), 9.51 (s, 1H, 4-CONH), 8.26 (s, 1H, 5-H), 7.96 (s, 1H, 3-H), 7.57 (s, 1H, 4″-H), 7.39 (d, *J* = 8.8 Hz, 1H, 6’-H), 7.22 (d, *J* = 2.8 Hz, 1H, 3’-H), 7.01 (dd, *J* = 8.8, 2.8 Hz, 1H, 5’-H), 6.96 (s, 1H, 5‴-H), 5.31 (s, 2H, OCH_2_), 4.12 (s, 2H, SCH_2_), 3.88 (s, 3H, NCH_3_), 2.29 (s, 6H, 4‴-CH_3_ and 6‴-CH_3_). δ_C_ (101 MHz; (CD_3_)_2_SO) 168.9 (C-2‴), 167.2 (2″-NHCO), 167.0 (C-4‴ and C-6‴), 160.7 (4-CONH), 158.8 (C-2″), 156.0 (C-4’), 138.8 (C-3), 138.1 (C-4″), 132.6 (C-5), 130.4 (C-2’), 129.6 (C-6’), 128.1 (C-1’), 126.1 (C-5″), 117.8 (C-4), 116.1 (C-5‴), 115.6 (C-3’), 114.4 (C-5’), 62.5 (OCH_2_), 38.8 (NCH_3_), 34.1 (SCH_2_), 23.2 (4‴-CH_3_ and 6‴-CH_3_). HRMS (ESI): *m*/*z* = [M+Na]^+^ calculated for C_23_H_22_ClN_7_O_3_S_2_Na^+^: 566.0812; found: 566.0798.

### 4.2. Biological Investigations

**Sirtuin assays**. The biological activity of the test substances against the respective sirtuin enzymes was evaluated by means of a fluorescence-based assay performed by Reaction Biology Corporation (Malvern, PA, USA). The sirtuin enzymes used in the assays were all obtained from in-house sources at Reaction Biology Corporation (Malvern, PA, USA). Sirt1, with accession number NM_012238, includes amino acids 1–747 (C-terminal) and is tagged with an N-terminal His-tag. Sirt1 was expressed in *E. coli* and purified to greater than 85% as confirmed by SDS-PAGE and is supplied as a solution in 50 mM Tris/HCl (pH 7.5), 100 mM NaCl, and 10% glycerol (*v*/*v*). Sirt2 (accession number NM_012237), comprising amino acids 50–389 (C-terminal), features an N-terminal His-tag and was expressed in *E. coli* and finally purified to over 90% by SDS-PAGE. Sirt2 is provided in a buffer containing 50 mM Tris/HCl (pH 7.5), 500 mM NaCl, 1 mM TCEP, and 10% glycerol (*v*/*v*). Sirt3, with accession number NM_012239.3, spans amino acids 101–399 and is tagged with an N-terminal GST and expressed in *E. coli* and purified to greater than 85% purity by SDS-PAGE. Sirt3 is supplied in 50 mM Tris/HCl (pH 7.5), 500 mM NaCl, and 10% glycerol (*v*/*v*). Sirt5 (accession number NM_012241), consisting of amino acids 37–310 (C-terminal) and tagged with an N-terminal His-tag, was expressed in *E. coli*, purified to greater than 95% purity by SDS-PAGE and delivered as a solution in 50 mM Tris/HCl (pH 7.5), 500 mM NaCl, 1 mM TCEP, and 10% glycerol (*v*/*v*). All proteins were provided in purified forms for use in the assays. The respective internal assay protocol is outlined in the following: After the test substances (dissolved in DMSO) were incubated with the desired sirtuin enzyme in assay buffer (Tris-HCl, pH = 8) for 10 min at 30 °C, a substrate mixture consisting of a 7-amino-4-methylcoumarin-linked fluorogenic peptide substrate (p53 residues 379–382) and the cofactor NAD^+^ was added. After 2 h of incubation at 30 °C, the universal inhibitor nicotinamide was added in excess to completely stop the deacetylation reaction, followed by the addition of a protease-based developer to release 7-amino-4-methylcoumarin, resulting in characteristic fluorescence, which was measured after 1 h at 30 °C (excitation/emission = 360/460). A no-inhibitor control was implemented, serving as a reference for 100% enzyme activity. IC_50_ values for Sirt2 were determined in triplicate by performing a 10-point, 3-fold serial dilution starting at a final reaction concentration of 50 µM or 100 µM when needed). Three individual IC_50_ values per test compound were calculated from the corresponding three dilution series fluorescence data using sigmoidal curve fitting in Prism 8.0.2 (GraphPad Software, Boston, MA, USA), and these were averaged to determine the mean IC_50_ value along with the standard deviation. To assess the selectivity against Sirt1, Sirt3, and Sirt5, a 50 µM concentration of the test compound was tested in duplicate, and the inhibitory activity was expressed as the mean of the duplicates in percentage of enzyme activity, including standard deviation, relative to the no-inhibitor control (representative 100% enzyme activity).

**Cell viability assay**. The cytotoxicity of the Sirt2 inhibitors was determined using a colorimetric MTT assay following Mosmann’s protocol [23]. HL-60 cells (purchased from DSMZ (German Collection of Microorganisms and Cell Cultures, Deutsche Sammlung von Mikroorganismen und Zellkulturen GmbH, Braunschweig, Germany) were cultivated in RPMI medium with 10% fetal bovine serum at 37 °C with 5% CO_2_ atmosphere. The cells were then seeded in a 96-well plate with a concentration of 9 x 10^4^ cells/well and a well volume of 99 µL. The cells were further incubated at 37 °C with 5% CO_2_ atmosphere for 24 h. The cells were then treated with 1 µL solution of Sirt2 inhibitors in various concentrations, which were prepared from 10 mM or 20 mM DMSO stock solutions. Negative controls were treated with 1 µL DMSO and positive controls with 1 µL of 1% Triton-X solution. Three technical replicates were performed. After incubation for 24 h, 10 µL MTT (5.0 mg/mL in PBS) were added to each well and the cells were incubated at 37 °C with 5% CO_2_ atmosphere for 2 h. Then 190 µL DMSO were added to each well and the plate shaken at room temperature at 600 rpm for 1 h with light exclusion. The absorbance of the MTT metabolite formazan was measured at 570 nm using the MRX Microplate Reader (Dynex Technologies, Chantilly, VA, USA). The means of the corresponding triplicates were calculated and set in relation to the 1% DMSO negative controls. The data was processed using Prism 8.0.2 (GraphPad Software, Boston, MA, USA) software and a logarithmic sigmoidal curve fitting for the calculation of the respective IC_50_ values was performed. IC_50_ values above 50 µM were considered as non-cytotoxic.

### 4.3. Computational Methods

Docking simulations were performed utilizing Schrödinger software suite (Schrödinger Inc., New York, NY, USA, version 2020-3) [24] using crystal structures of Sirt2 and respective lead structures, imported from the Protein Data Bank (PDB) [25] (**SirReal2**: PDB ID: 4RMG [14]) (**Yang_24a:** PDB ID: 5YQO [19]) and prepared with the Protein Preparation Wizard (Schrödinger Inc. New York City, USA). Protonation and charge calculation were done using Epik and the respective ligands were prepared with Ligand Preparation Wizard (Schrödinger Inc., New York, NY, USA) [26]. The docking calculations were carried out using Glide in standard precision mode SP and all docking parameters left to their default values. Pymol 2.5.8 (Schrödinger Inc., New York, NY, USA) was used for visualization purposes. The top ranked poses were analysed, considering the favourable spatial orientation in relation to the crystal structures of the corresponding lead compounds. The docking grid center was located at coordinates x = −13.495516, y = −10.113182, and z = −18.406236. The dimensions for the INNERBOX were set to 10, while the OUTERBOX had a size of 30.6493 for all x, y, and z axes. A total of five poses per ligand were saved, with ligands being treated as flexible and subjected to 10 post-docking minimizations. All other parameters were left at their default settings. For redocking, the ligand L5C from the X-ray structure 5YQO was used, and the docking procedure yielded an exceptionally low RMSD value of 0.89, which suggests that this method is highly effective for calculating Sirt2 ligand interactions. Visualization of the results was performed using PyMOL 2.5.8 (Schrödinger Inc.). The poses with the most favourable docking scores were analysed, with particular attention paid to the orientation of the pyrimidine ring respectively the selectivity pocket binder moiety in general, ensuring it closely resembled the positioning found in the crystal structures of related lead compounds. The ligand binding energies were calculated using PRIME MM-GBSA [27]. The docking poses were taken as starting points, solvation model = VSDB, force field = OPLS4 and sampling method = minimize. All protein atoms surrounding the ligands were treated as flexible during the MM-GBSA calculations. ADMET calculations were performed utilizing QikProp, version 4.3 (Schrödinger Inc., New York, NY, USA).

## Figures and Tables

**Table 1:** Inhibitory activity on Sirt2 and corresponding subtype selectivity towards Sirt1, Sirt3 and Sirt5 of respective inhibitors was determined by Reaction Biology Corporation (Malvern, PA, USA) using fluorescence-based assays. IC_50_ values were obtained for each serial dilution within the triplicate set through sigmoidal curve fitting, and the mean with standard deviation (SD) was calculated from these three values. Residual enzyme activity of Sirt1, Sirt3, and Sirt5 was measured as a percentage at a final inhibitor concentration of 50 µM, based on duplicate samples, and the mean with standard deviation (SD) was calculated thereof. Abbreviation: n.d.: not determined.

ID	Chemical Structure	IC_50_ [µM] ± SD Sirt2	Residual Enzyme Activity [%] ± SD		
			Sirt1	Sirt3	Sirt5
**SirReal2**	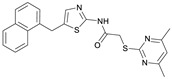	0.235 ± 0.008	n.d.	n.d.	n.d.
**Yang_24a**	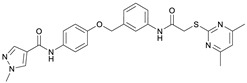	0.0790 ± 0.0030	n.d.	n.d.	n.d.
**Yang_28e**	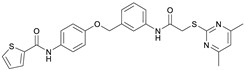	0.0867 ± 0.0085	n.d.	n.d.	n.d.
**FM358**	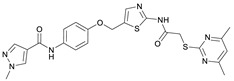	0.0656 ± 0.0044	71.9 ± 0.6	62.5 ± 0.1	79.8 ± 0.6
**FM352**	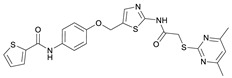	0.0935 ± 0.0064	101 ± 1	75.8 ± 5.3	75.6 ± 1.1
**FM345**	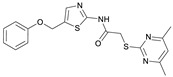	5.29 ± 0.73	95.6 ± 2.3	78.8 ± 0.8	94.6 ± 2.3
**FM368**	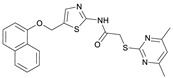	6.51 ± 0.84	88.5 ± 2.7	85.0 ± 0.5	89.1 ± 1.4

**Table 2:** Inhibitory activity on Sirt2 and corresponding subtype selectivity towards Sirt1, Sirt3 and Sirt5 of respective inhibitors was determined by Reaction Biology Corporation (Malvern, PA, USA) using fluorescence-based assays. IC_50_ values were obtained for each serial dilution within the triplicate set through sigmoidal curve fitting, and the mean with standard deviation (SD) was calculated from these three values. Residual enzyme activity of Sirt1, Sirt3, and Sirt5 was measured as a percentage at a final inhibitor concentration of 50 µM, based on duplicate samples, and the mean with standard deviation (SD) was calculated thereof.

ID	Chemical Structure	IC_50_ [µM] ± SD Sirt2	Residual Enzyme Activity [%] ± SD		
			Sirt1	Sirt3	Sirt5
**RW-99**	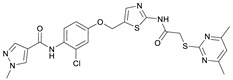	0.0250 ± 0.0026	53.9 ± 0.2	56.1 ± 0.1	55.1 ± 2.0
**RW-93**	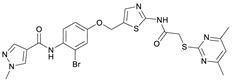	0.0155 ± 0.0007	58.2 ± 0.2	62.3 ± 1.2	53.0 ± 0.2
**RW-95**	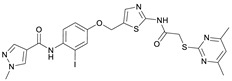	0.0485 ± 0.0056	60.9 ± 0.7	64.2 ± 1.2	48.2 ± 1.8

**Table 3:** Validation studies involving a comparative analysis of the estimated docking scores as well as the calculated free binding energies of the investigated Sirt2 inhibitors with their respective experimentally determined in vitro IC_50_ values. The correlation factor between the estimated docking scores and the IC_50_ values is 0.68, and the correlation factor between the calculated free binding energies (∆G) and the IC_50_ values is 0.73.

ID	Chemical Structure	IC_50_ [µM] ± SD Sirt2	Docking Score [kcal/mol]	∆G [kJ/mol]
**SirReal2**	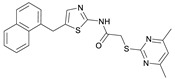	0.235 ± 0.008	−9.39	−81.59
**Yang_24a**	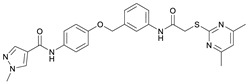	0.0790 ± 0.0030	−13.16	−130.59
**Yang_28e**	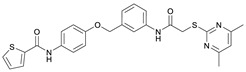	0.0867 ± 0.0085	−12.58	−126.99
**FM345**	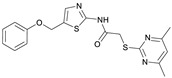	5.29 ± 0.73	−9.07	−77.87
**FM352**	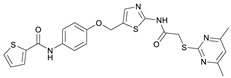	0.0935 ± 0.0064	−11.97	−111.29
**FM358**	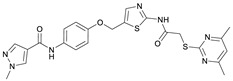	0.0656 ± 0.0044	−12.51	−110.30
**FM368**	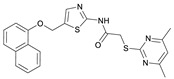	6.51 ± 0.84	−9.90	−79.76
**RW-93**	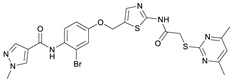	0.0155 ± 0.0007	−14.63	−110.82
**RW-95**	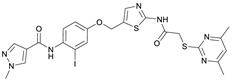	0.0485 ± 0.0056	−14.65	−104.97
**RW-99**	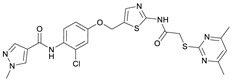	0.0250 ± 0.0026	−14.30	−115.51

**Table 4:** ADME properties calculated by QikProp. S (Stars) = Number of property/descriptor values falling outside the 95% range of similar values for known drugs. Recommended values 0 to 5. MW = Molecular weight. QPlogPo/w = Predicted octanol/water partition coefficient. Recommended values −2.0 to 6.5. QPlogS = Predicted aqueous solubility. Recommended values −6.5 to 0.5. QPPCaco = Predicted apparent Caco-2 cell permeability in nm/sec. Recommended values < 25 poor, >500 great. QPlogBB = Predicted brain/blood partition coefficient. Recommended values −3.0 to 1.2. HOA = Predicted human oral absorption on 0 to 100% scale. Recommended values < 25% poor, >80% high. RO5 = Number of violations of Lipinski’s rule of five. The rules are: mol. MW < 500, QPlogPo/w < 5, donorHB ≤ 5, accptHB ≤ 10. Recommended value < 4.

ID	S	MW	QPlogPo/w	QPlogS	QPP Caco	QPlogBB	HOA (%)	RO5
**FM358**	3	509.6	4.117	−7.991	232.950	−2.063	80.460	1
**RW-93**	3	588.5	4.557	−8.371	265.051	−1.808	84.039	1
**RW-95**	4	635.5	4.659	−8.529	264.457	−1.805	84.623	1
**RW-99**	3	544.0	4.506	−8.359	244.444	−1.872	83.112	1

## Data Availability

Experimental data and protocols are stored in the University’s electronic lab journal at LMU Munich. The original contributions presented in this study are included in the article/supplementary material. Further inquiries can be directed to the corresponding author.

## Supplementary Materials

The following supporting information can be downloaded at: https://www.mdpi.com/article/10.3390/ijms26209855/s1.

## Abbreviations

The following abbreviations are used in this manuscript:

AcOH	Acetic acid
ADP	Adenosine diphosphate
DCM	Dichloromethane
DMF	Dimethylformamide
DMSO	Dimethylsulfoxide
EtOAc	Ethyl acetate
MeOH	Methanol
NAD^+^	Nicotinamide adenine dinucleotide
SAR	Structure–activity relationship
Sirt	Sirtuin
*t*-BuOK	Potassium tert-butoxide
Val	Valine
