# Supplementary material for: Lead Structure-Based Hybridization Strategy Reveals Major Potency Enhancement of SirReal-Type Sirt2 Inhibitors
Source: Int J Mol Sci. 2025 Oct 10;26(20):9855. doi: 10.3390/ijms26209855 (PMC12563821; doi:10.3390/ijms26209855)
Supplement: Supplementary file 1 [file ijms-26-09855-s001.zip › ijms-3882958-supplementary.pdf]

Supplementary Information

Lead structure-based hybridization strategy reveals major potency enhancement of SirReal-type Sirt2 inhibitors

Matthias Frei*, Ricky Wirawan*, Thomas Wein and Franz Bracher **

Department of Pharmacy—Center for Drug Research, Ludwig-Maximilians University, Butenandtstr. 5–13, 81377 Munich, Germany;

matthias.frei@cup.uni-muenchen.de (M.F.); ricky.wirawan@cup.uni-muenchen.de (R.W.); thomas.wein@cup.uni-muenchen.de (T.W.).

* Equal contribution to this paper
** Correspondence: franz.bracher@cup.uni-muenchen.de; Tel.: +49-89-218077301

Table of Contents:

^{1}H and ^{13}C NMR spectra of compounds 2

HPLC chromatograms of tested compounds 18

IR spectra of tested compounds 23

Figure S1: Cell viability data of **RW-93** 28

^{1}H (400 MHz; $(CD_3)_2SO$) and ^{13}C (101 MHz; $(CD_3)_2SO$) NMR spectra of compound **2**

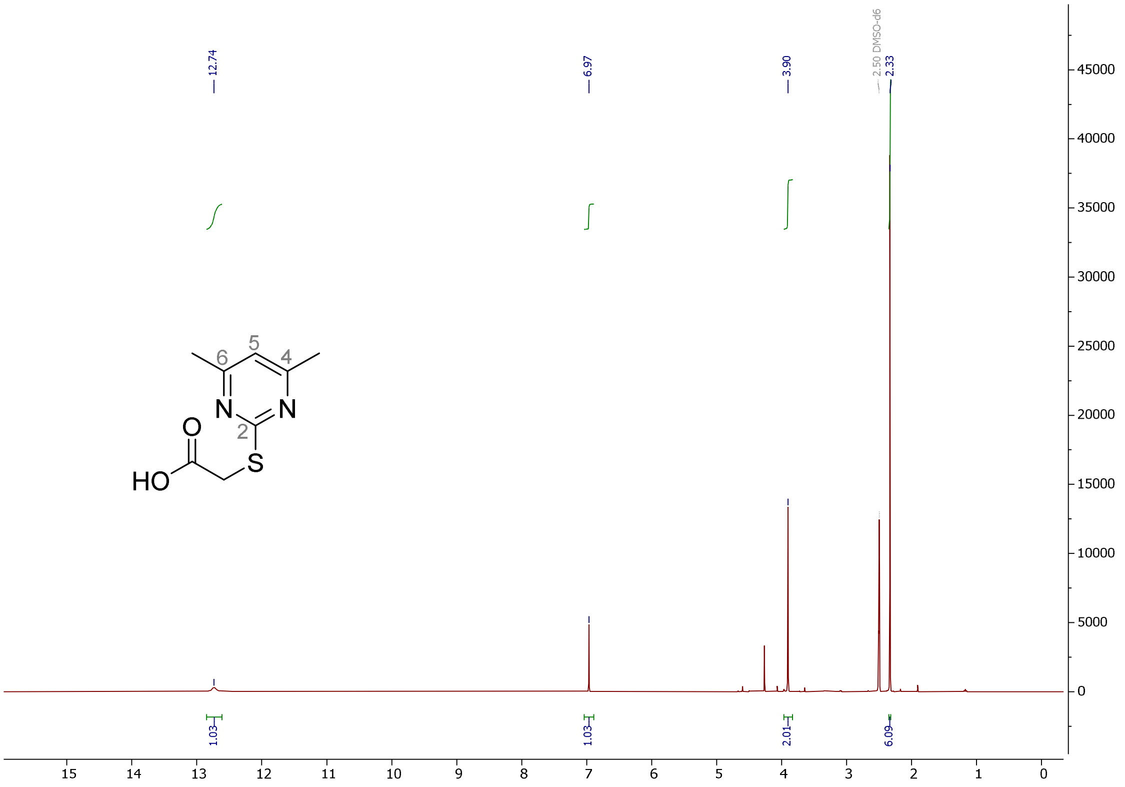

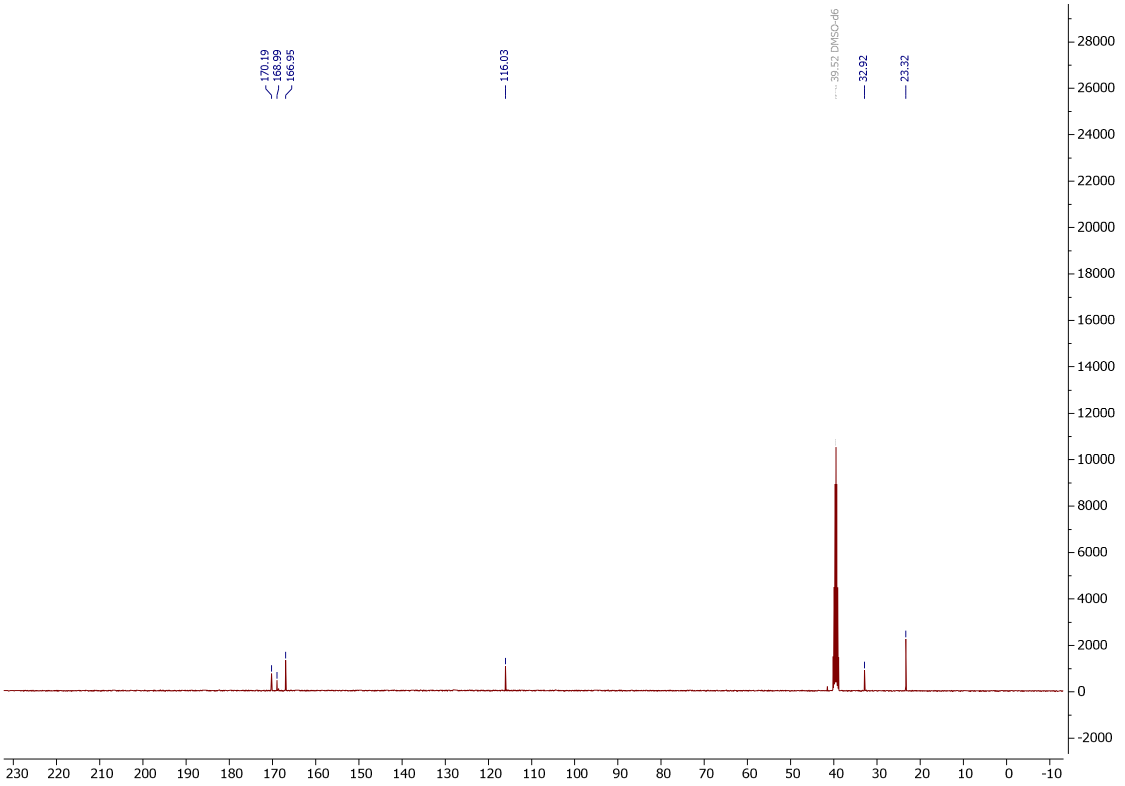

^{1}H (400 MHz; $(CD_3)_2SO$) and ^{13}C (101 MHz; $(CD_3)_2SO$) NMR spectra of compound **3**

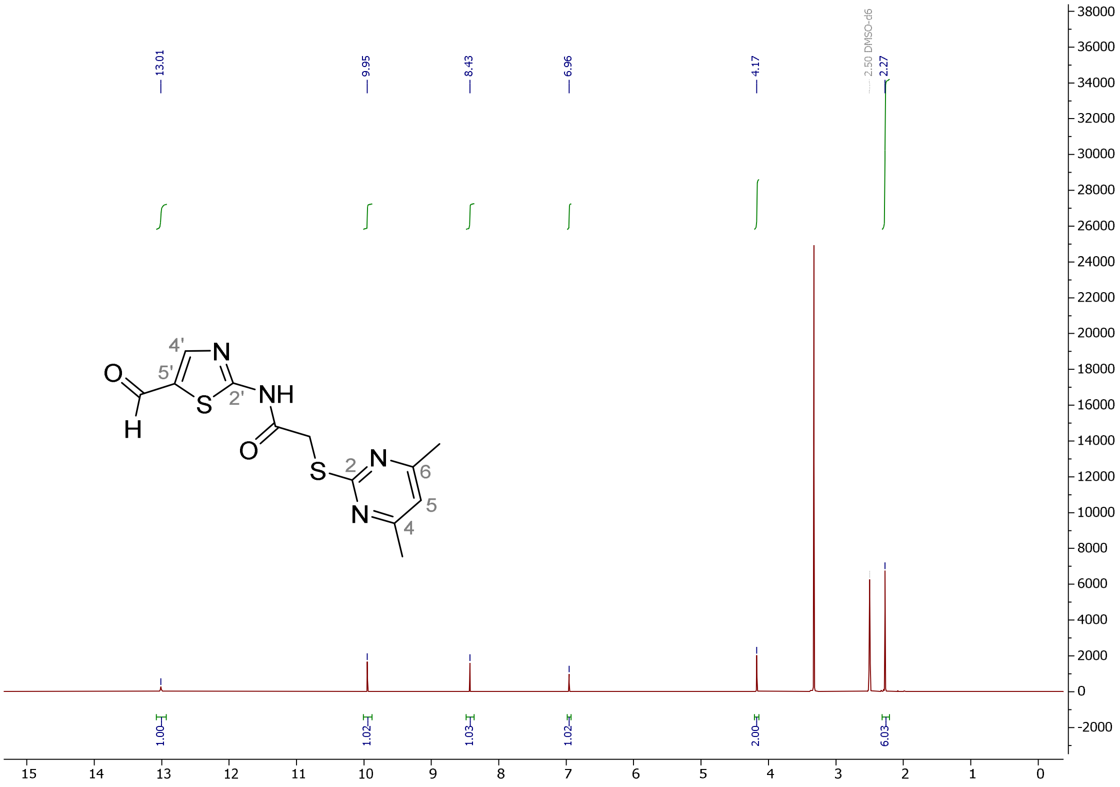

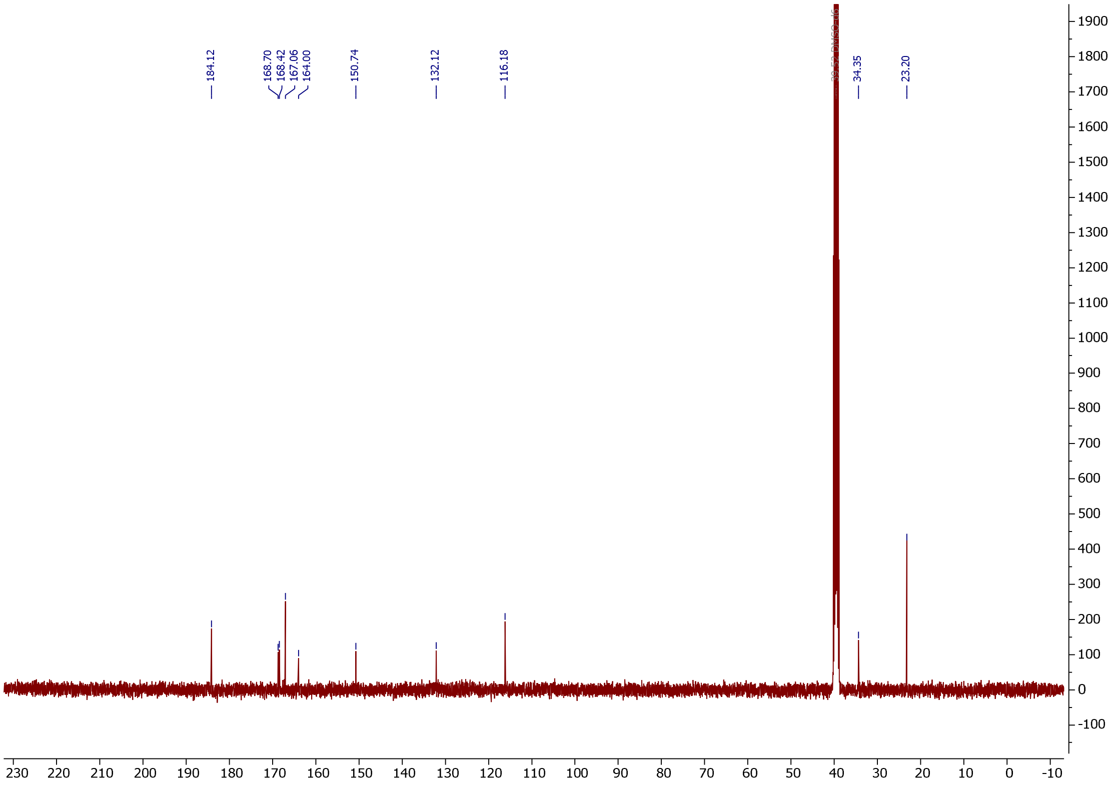

^{1}H (400 MHz; $(CD_3)_2SO$) and ^{13}C (101 MHz; $(CD_3)_2SO$) NMR spectra of compound **4**

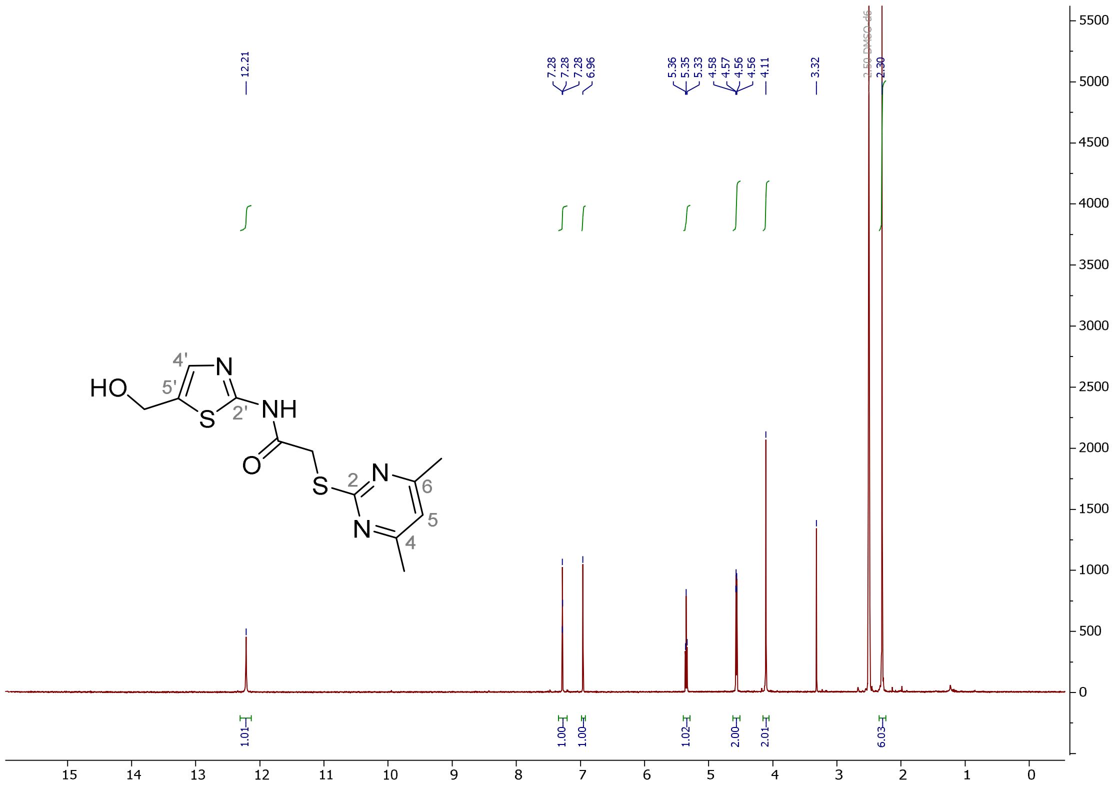

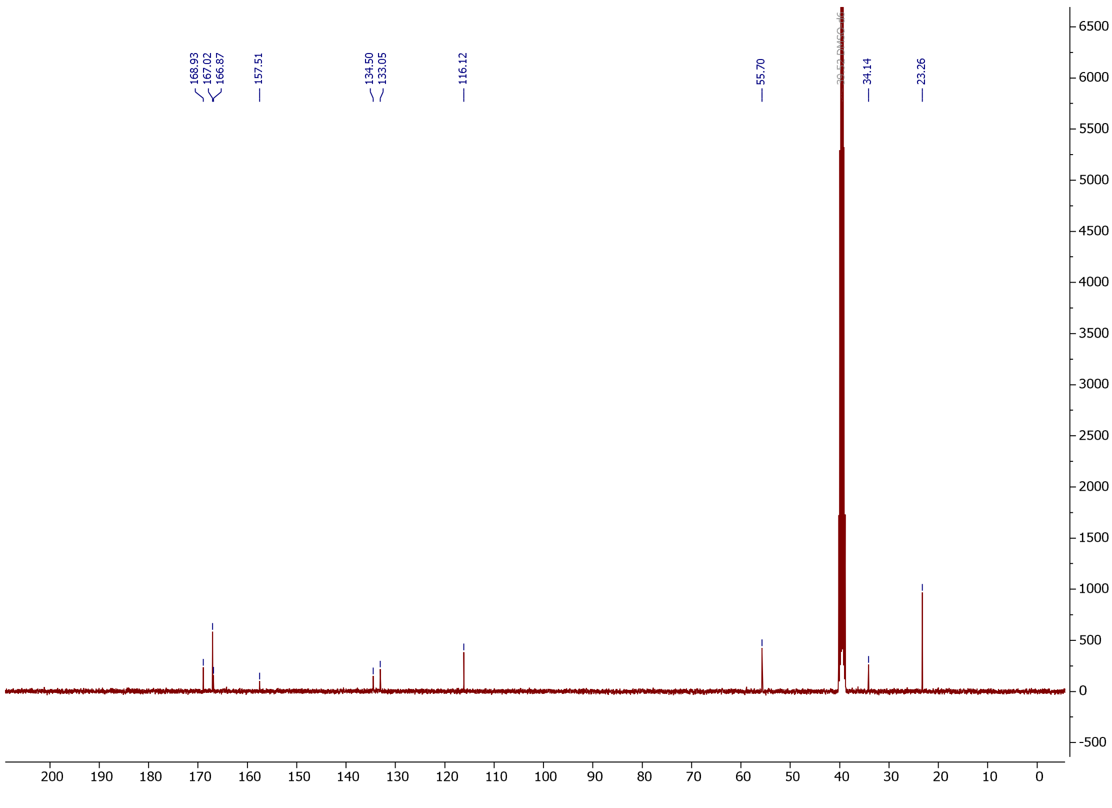

^{1}H (400 MHz; $(CD_3)_2SO$) and ^{13}C (101 MHz; $(CD_3)_2SO$) NMR spectra of compound **5**

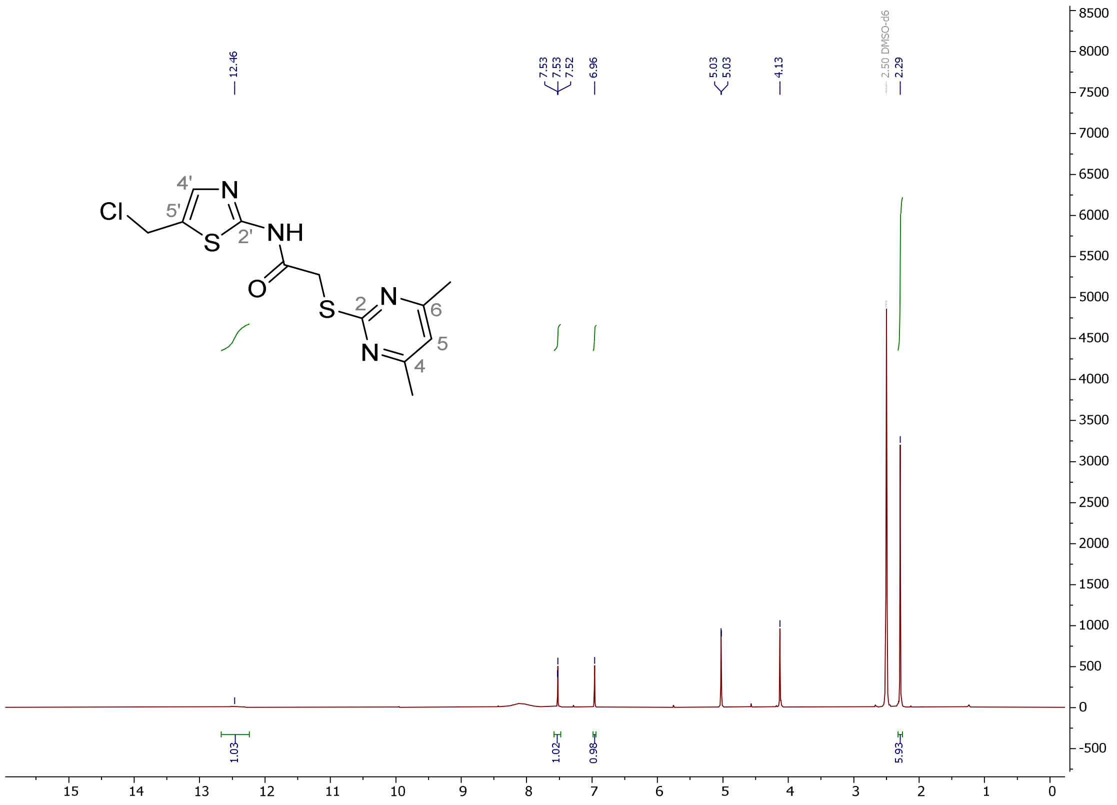

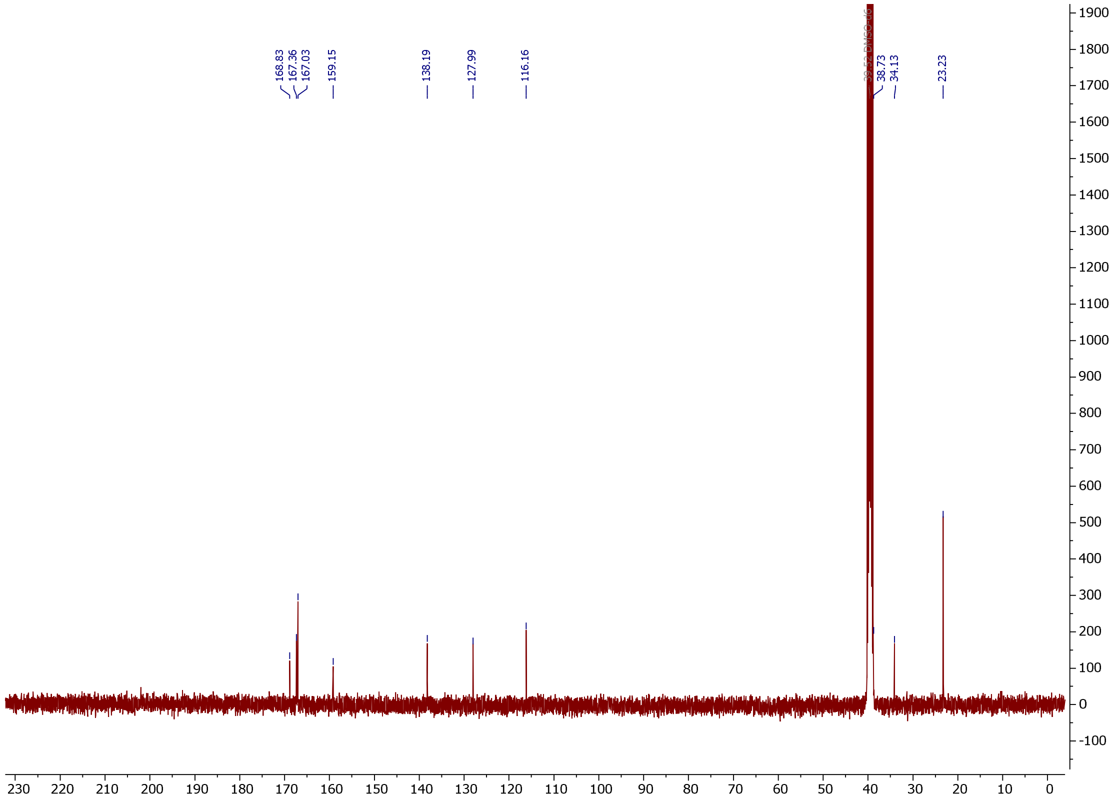

^1H (500 MHz; $(CD_3)_2SO$) and ^{13}C (126 MHz; $(CD_3)_2SO$) NMR spectra of **FM345**

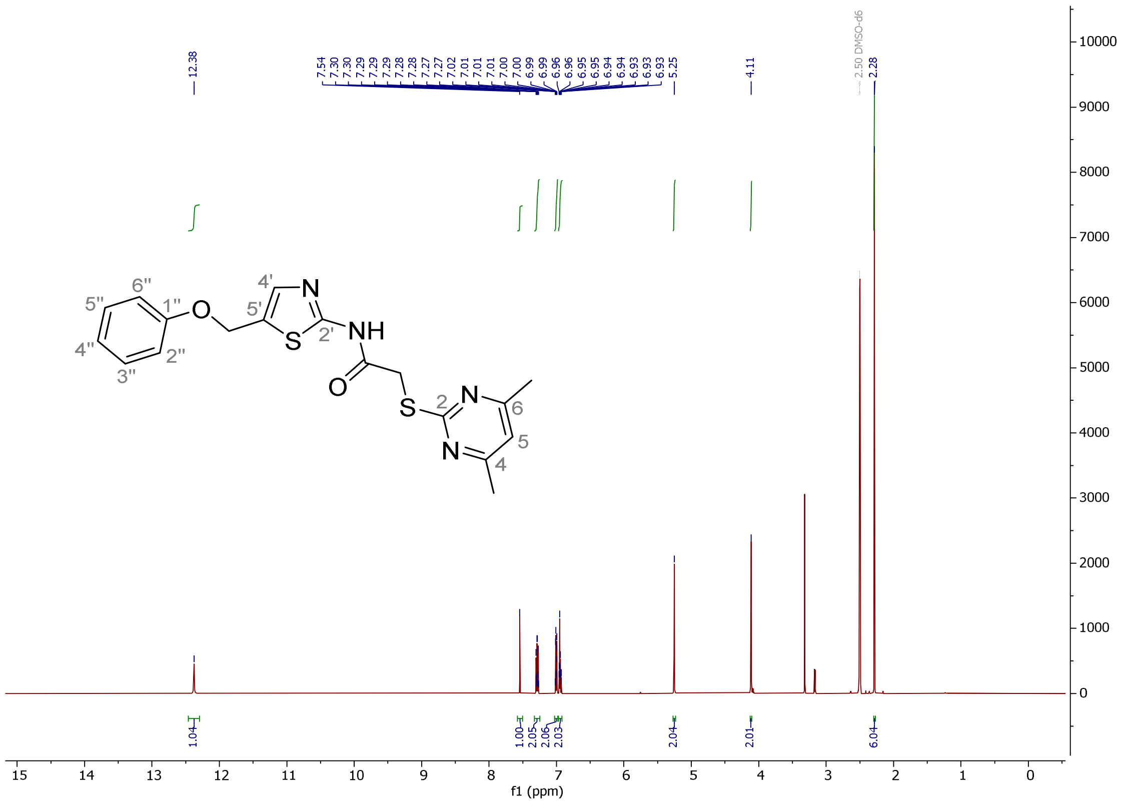

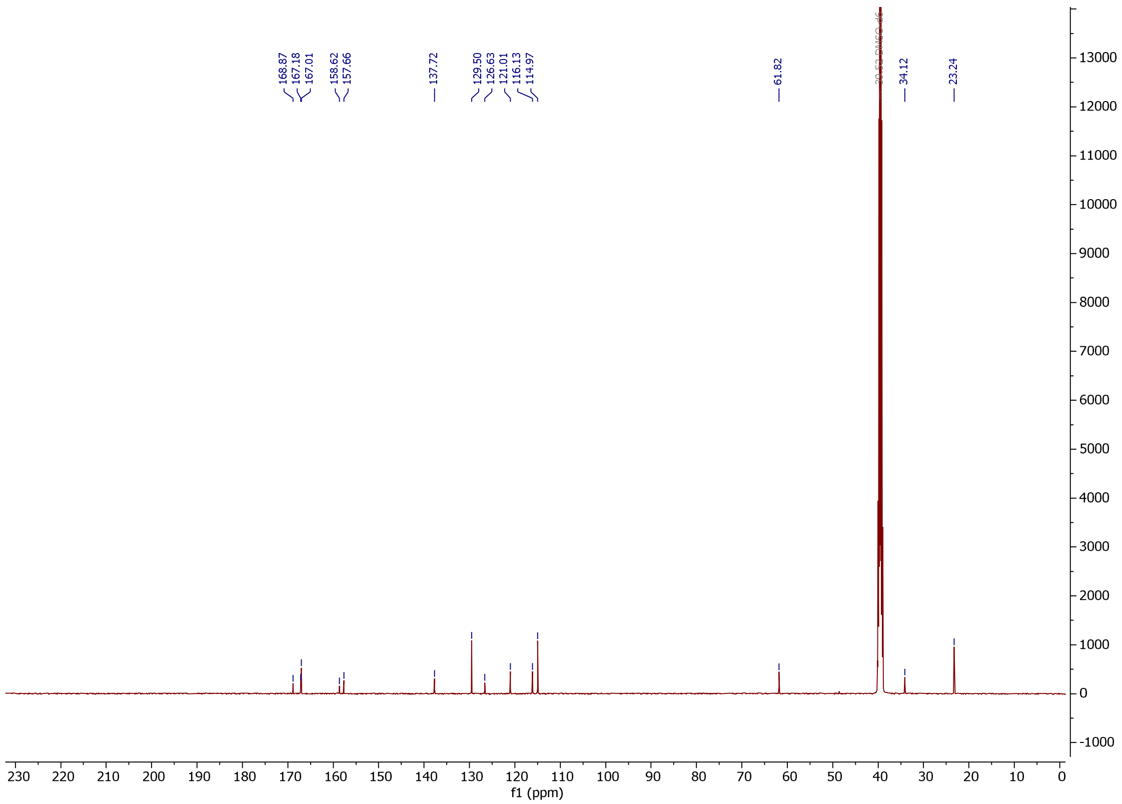

^{1}H (400 MHz; $(CD_3)_2SO$) and ^{13}C (101 MHz; $(CD_3)_2SO$) NMR spectra of compound **7**

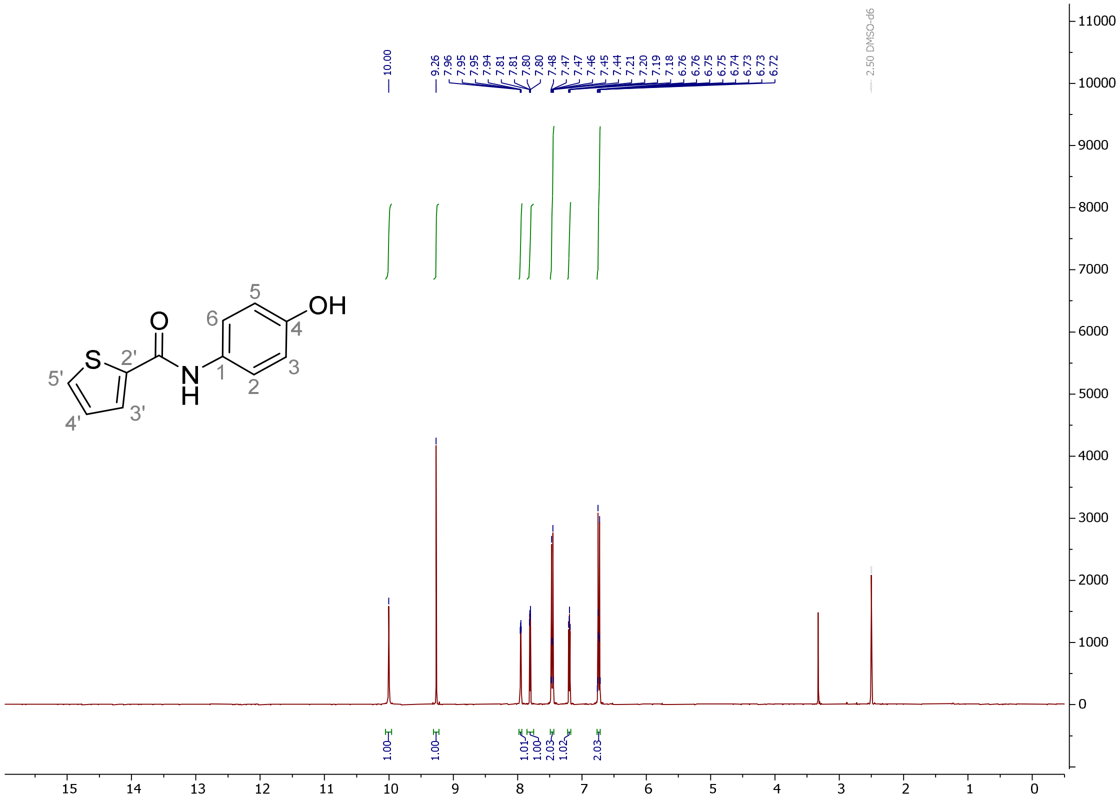

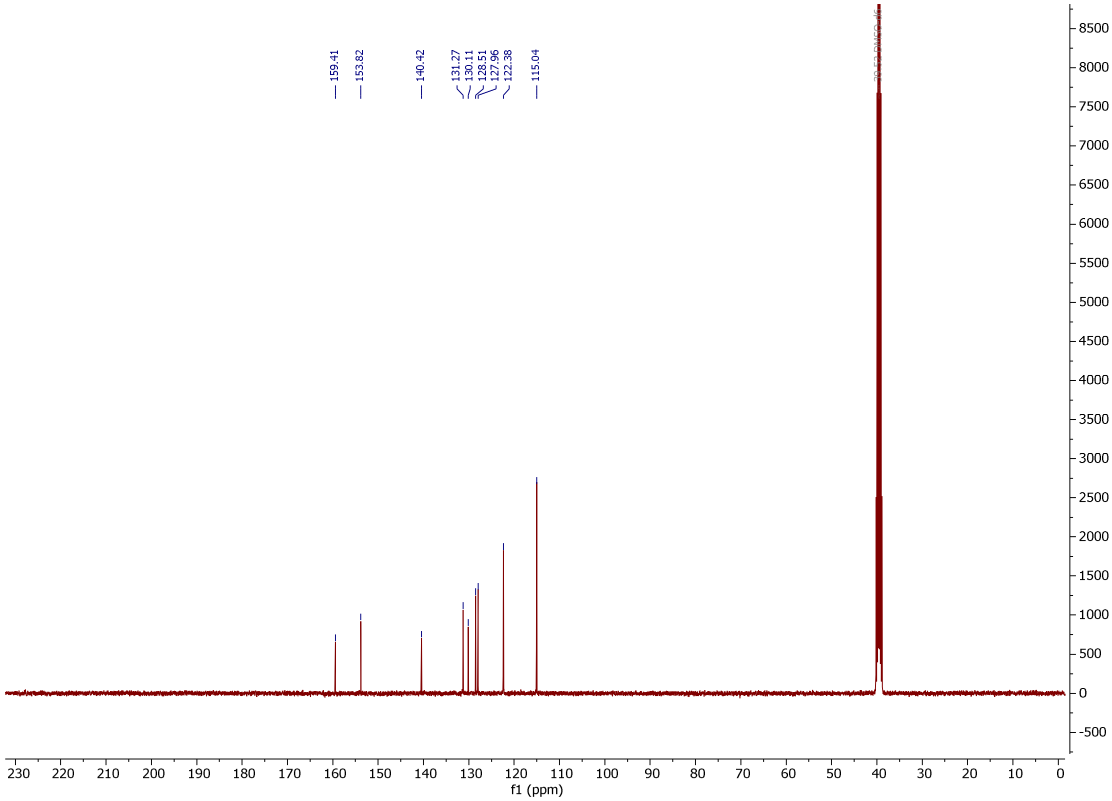

^{1}H (400 MHz; $(CD_3)_2SO$) and ^{13}C (101 MHz; $(CD_3)_2SO$) NMR spectra of compound **8**

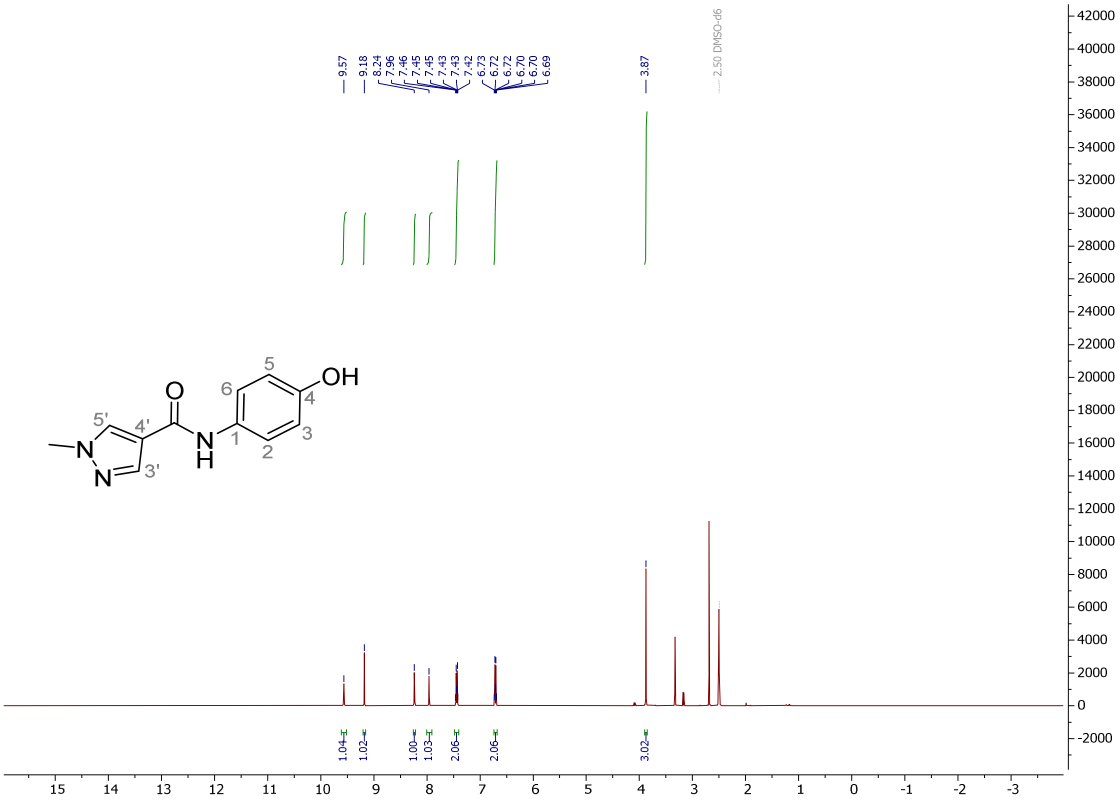

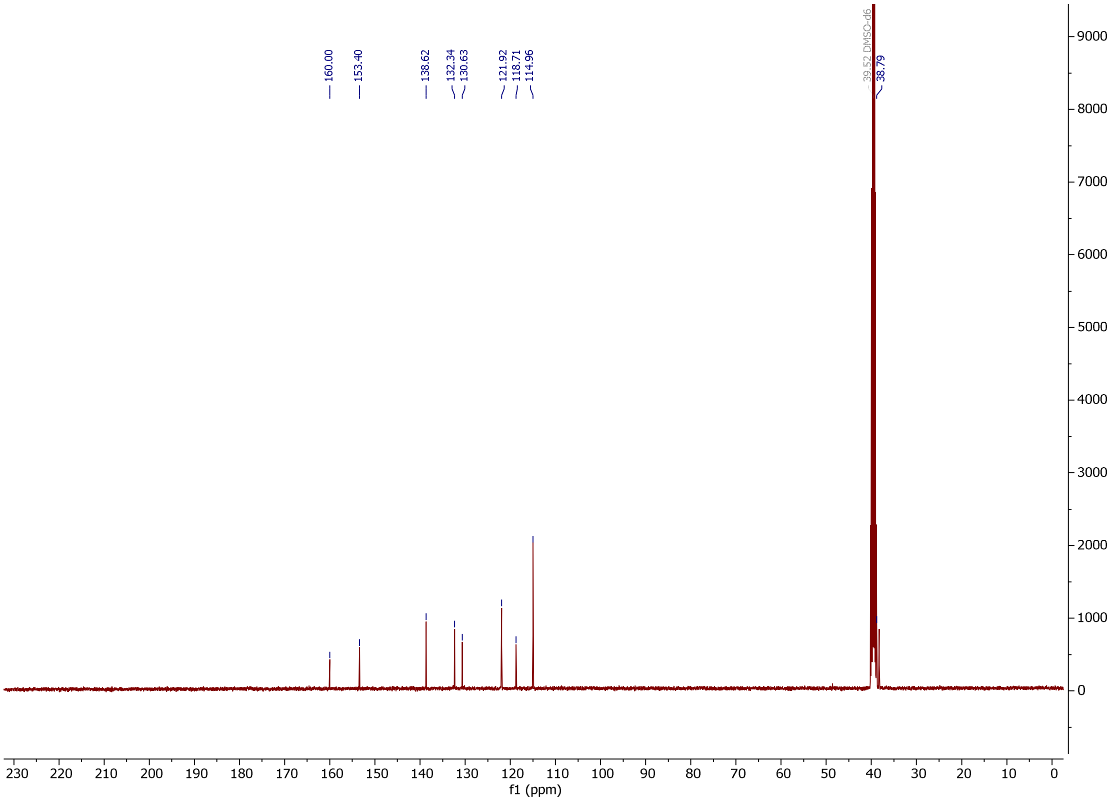

^{1}H (500 MHz; $(CD_3)_2SO$) and ^{13}C (126 MHz; $(CD_3)_2SO$) NMR spectra of **FM352**

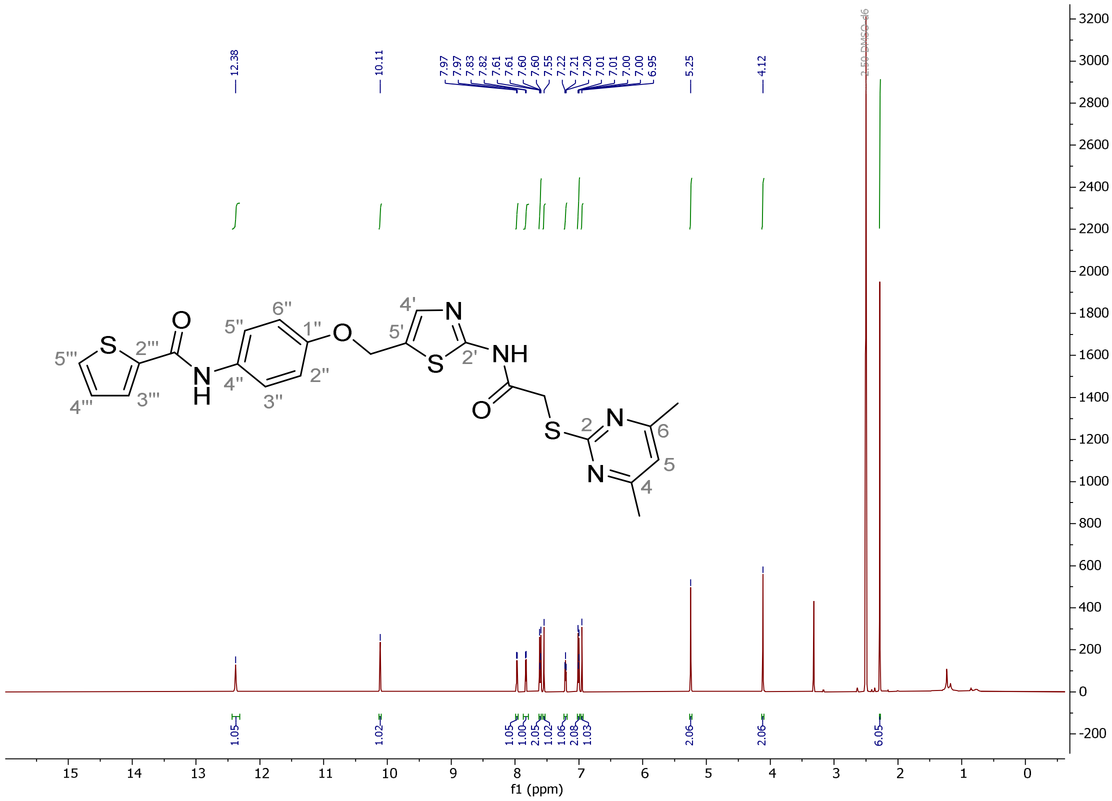

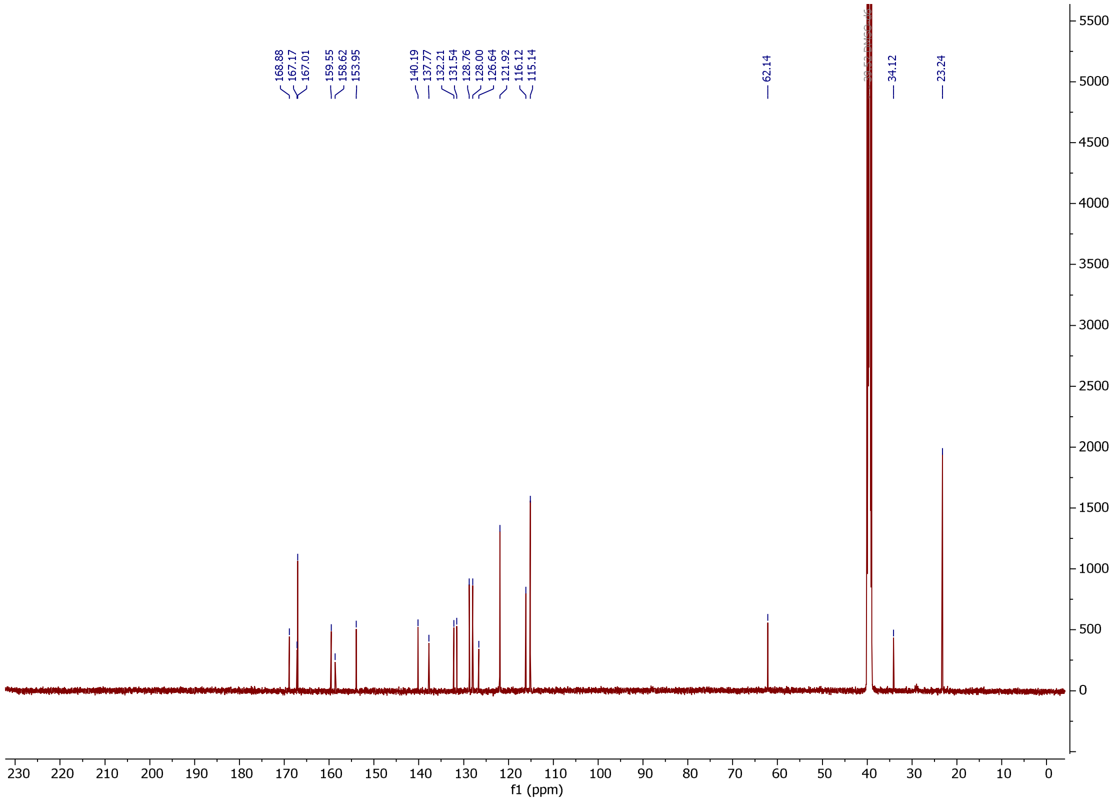

^{1}H (500 MHz; $(CD_3)_2SO$) and ^{13}C (126 MHz; $(CD_3)_2SO$) NMR spectra of **FM358**

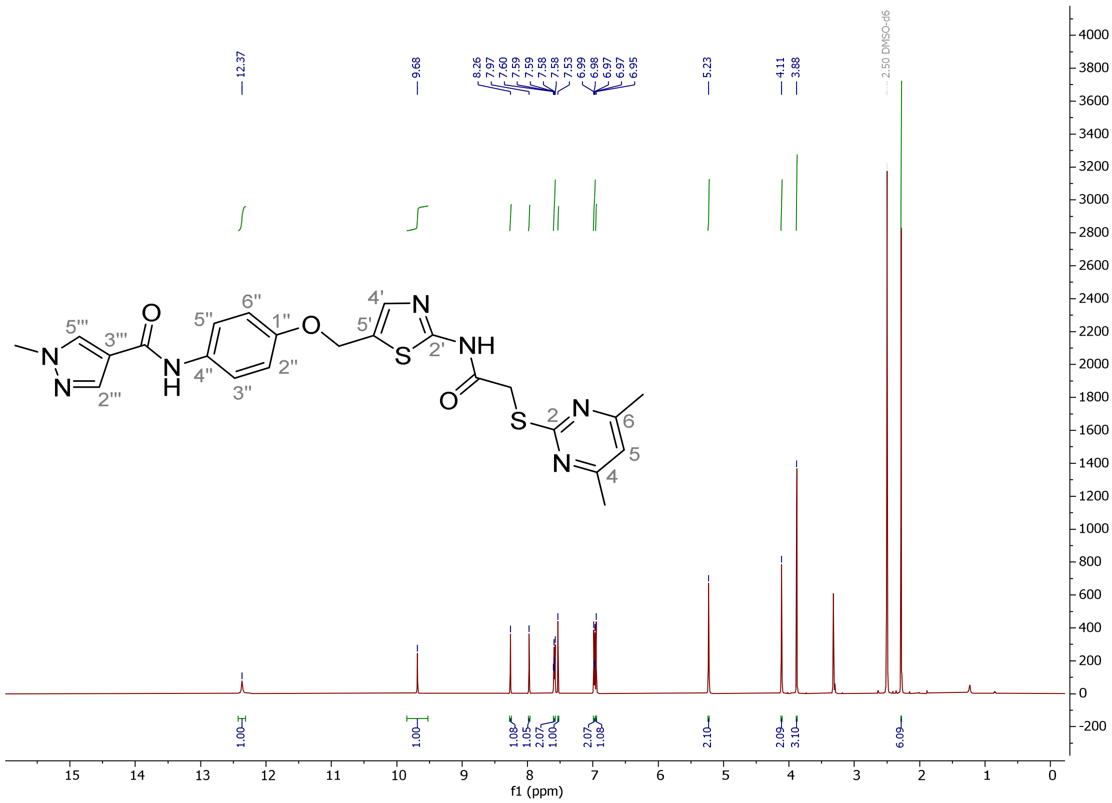

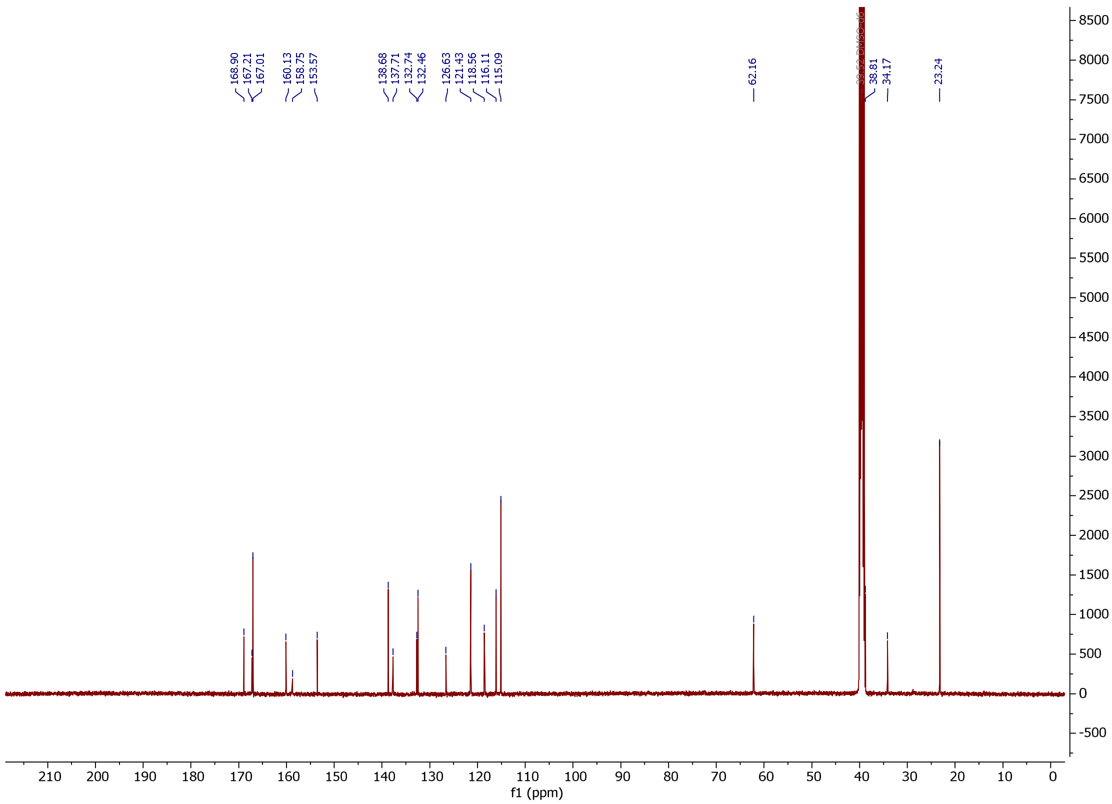

^{1}H (500 MHz; $(CD_3)_2SO$) and ^{13}C (126 MHz; $(CD_3)_2SO$) NMR spectra of **FM368**

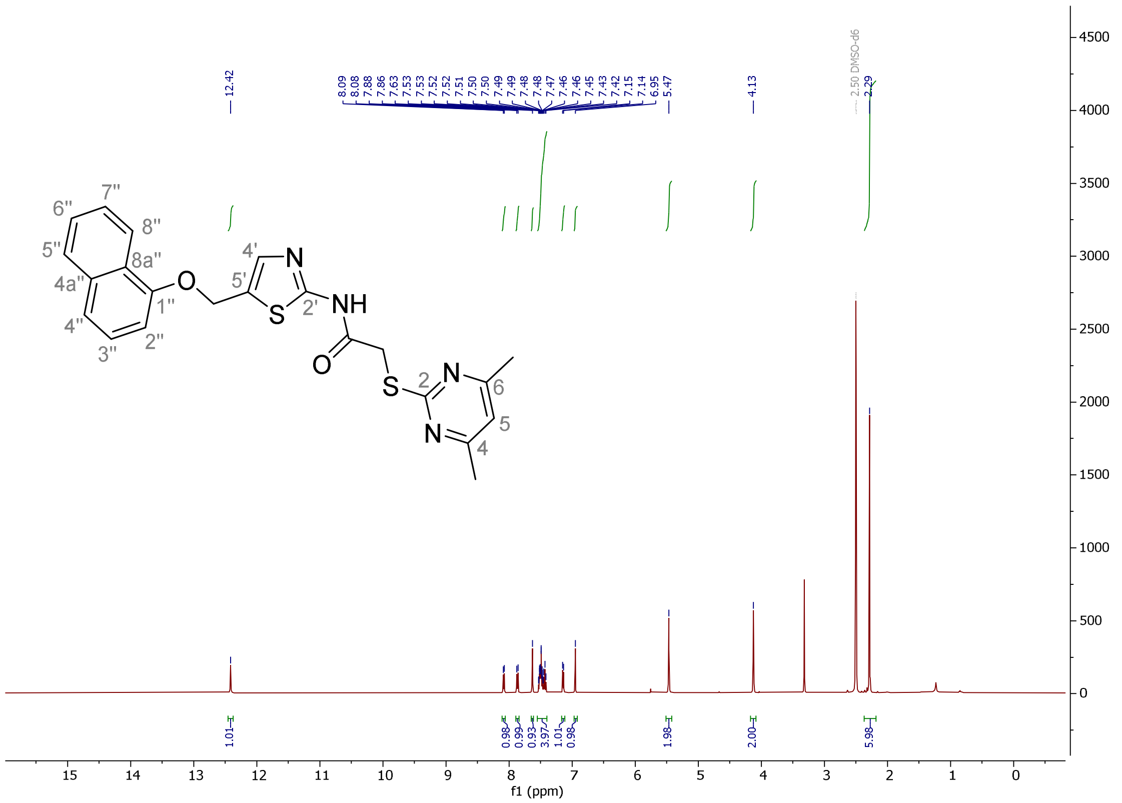

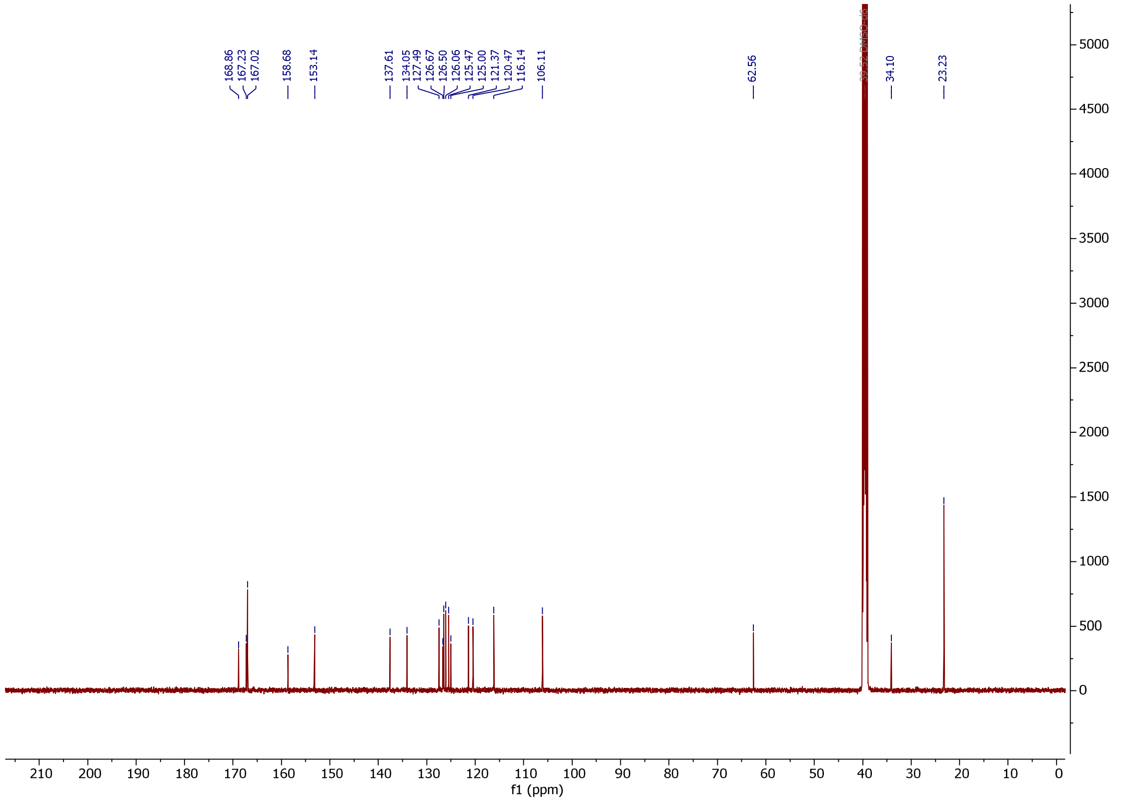

^{1}H (400 MHz; $(CD_3)_2SO$) and ^{13}C (126 MHz; $(CD_3)_2SO$) NMR spectra of compound **9**

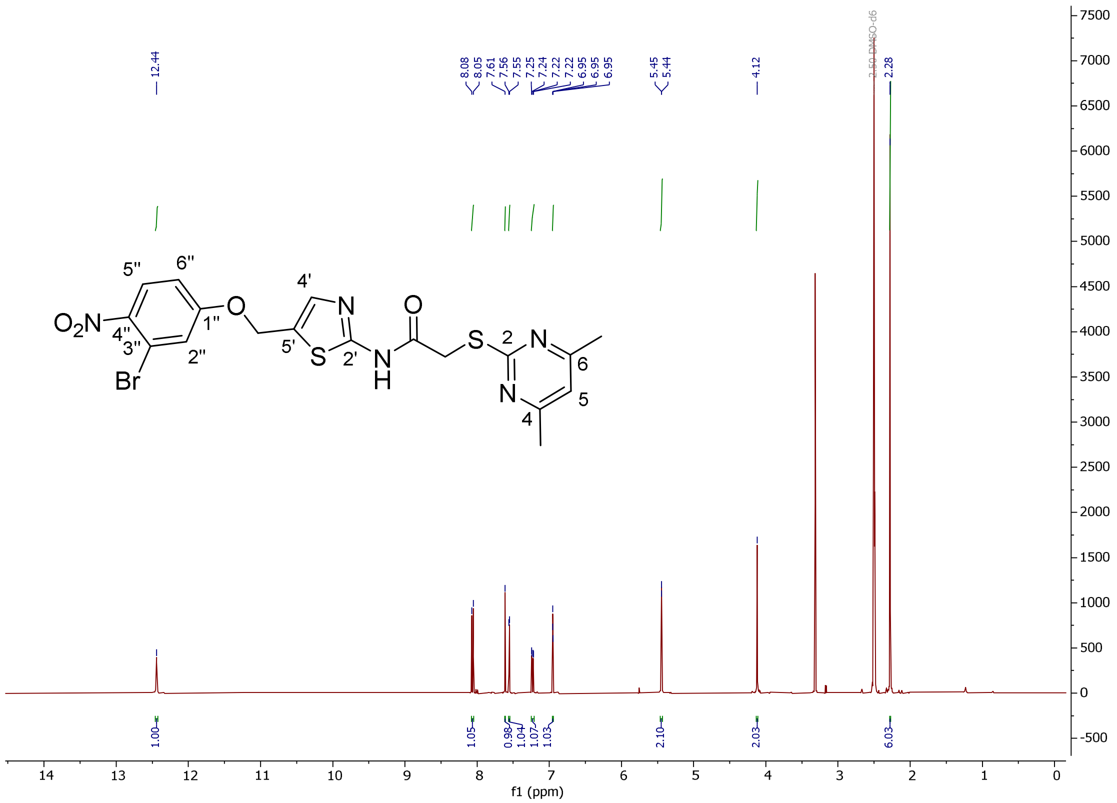

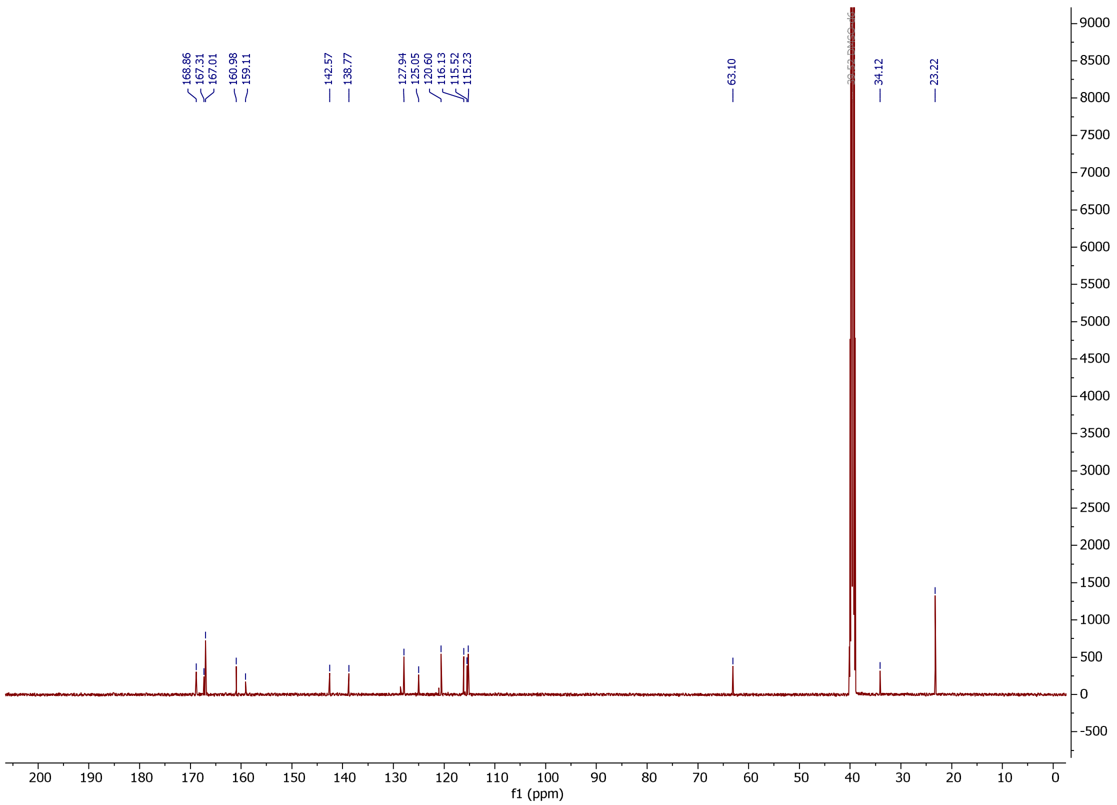

^{1}H (400 MHz; $(CD_3)_2SO$) and ^{13}C (101 MHz; $(CD_3)_2SO$) NMR spectra of compound **10**

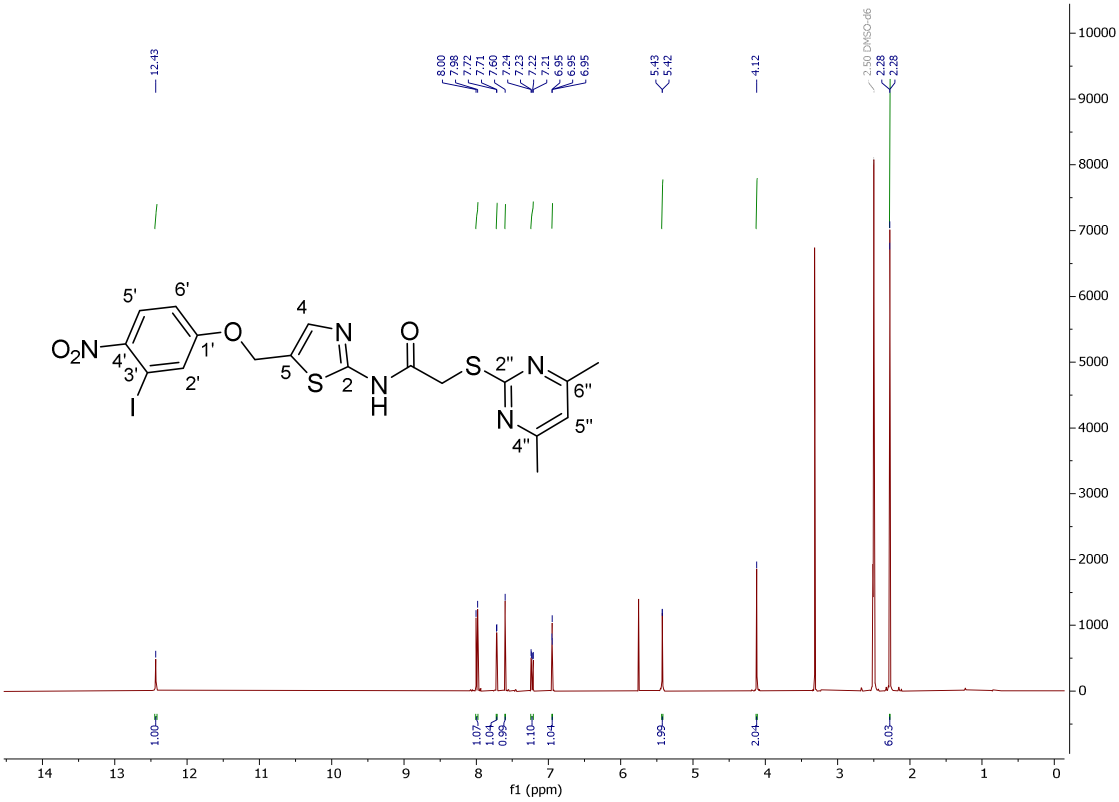

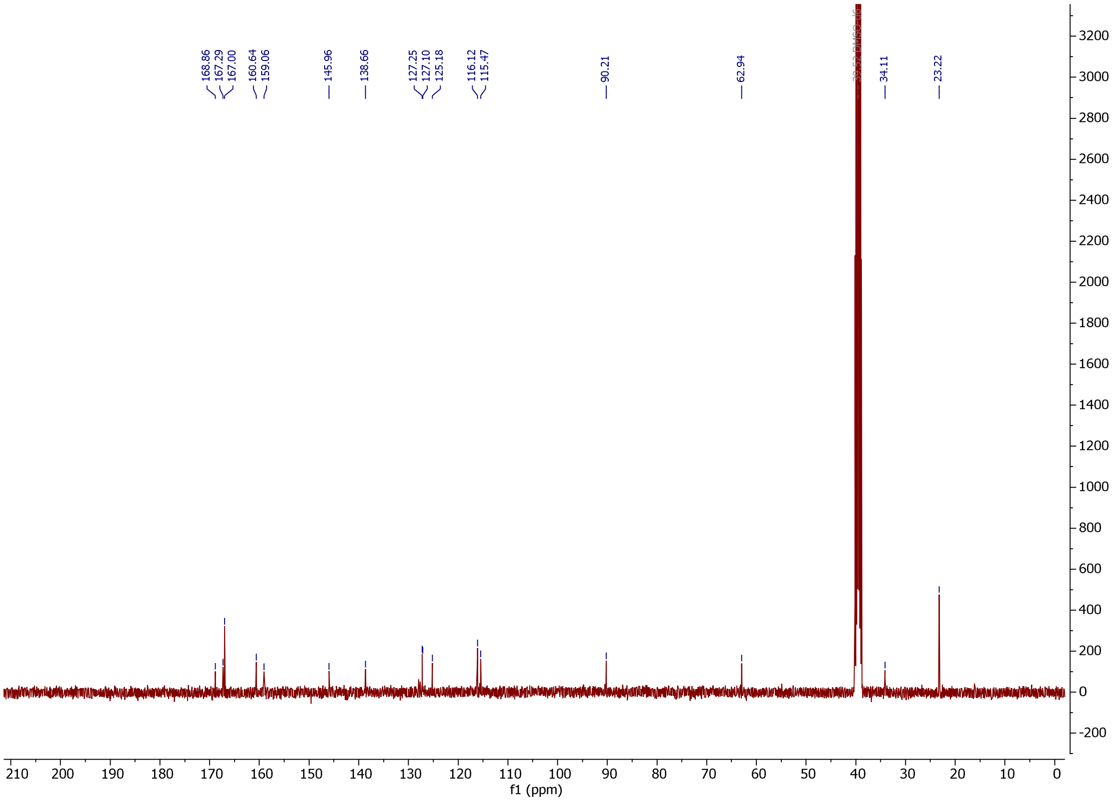

^{1}H (400 MHz; $(CD_3)_2SO$) and ^{13}C (101 MHz; $(CD_3)_2SO$) NMR spectra of compound **11**

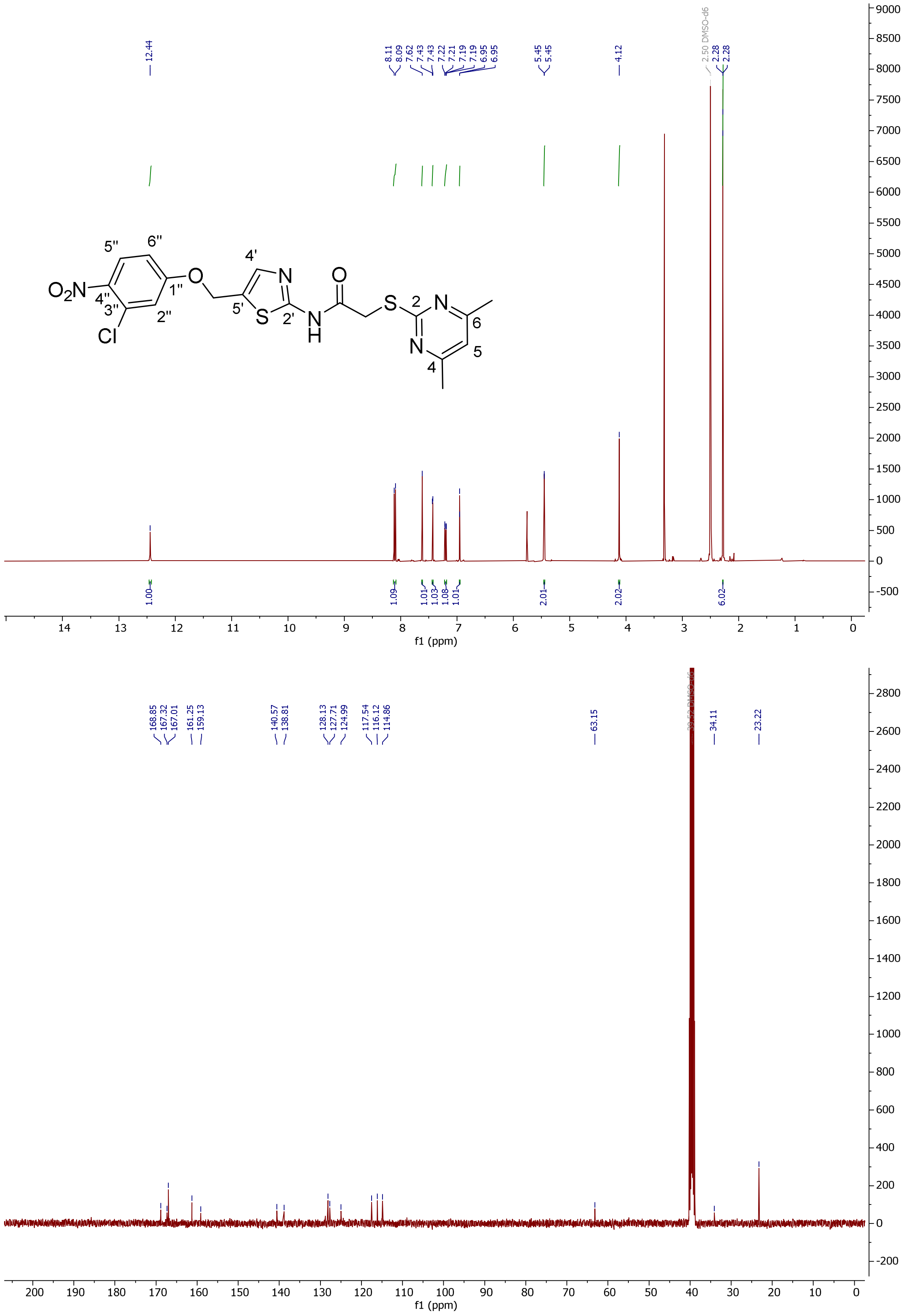

^{1}H (400 MHz; $(CD_3)_2SO$) and ^{13}C (101 MHz; $(CD_3)_2SO$) NMR spectra of **RW-93**

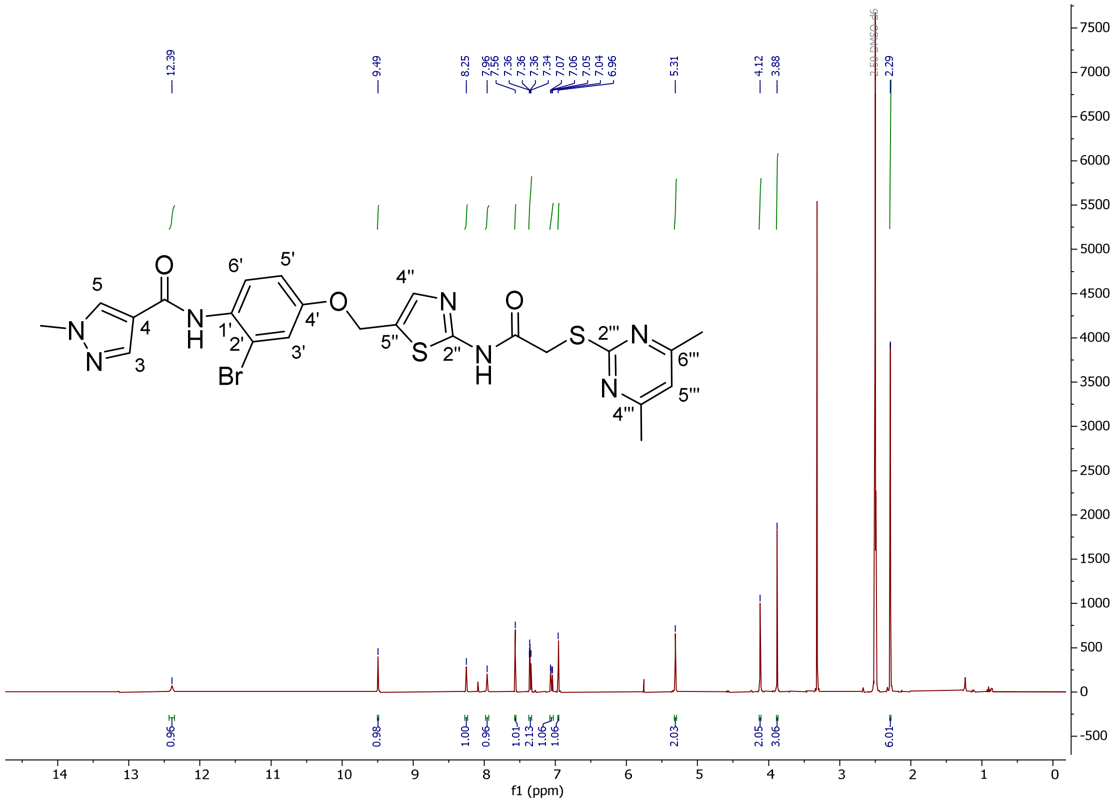

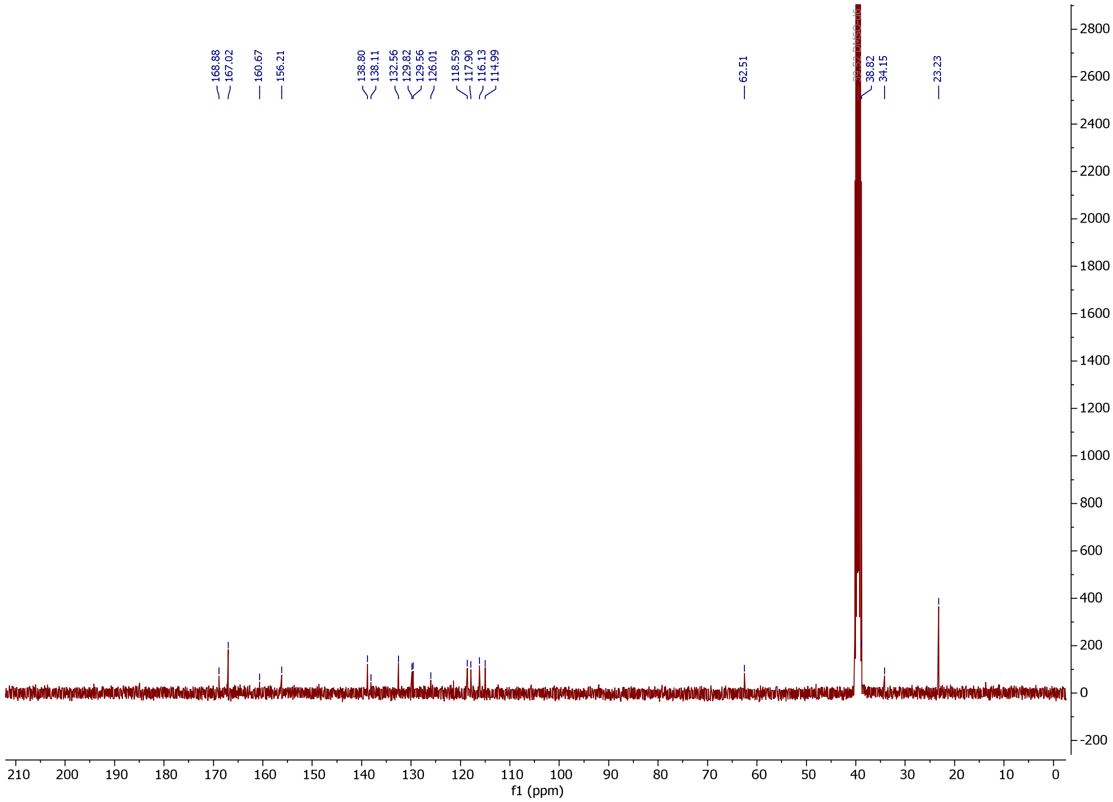

^{1}H (500 MHz; $(CD_3)_2SO$) and ^{13}C (126 MHz; $(CD_3)_2SO$) NMR spectra of **RW-95**

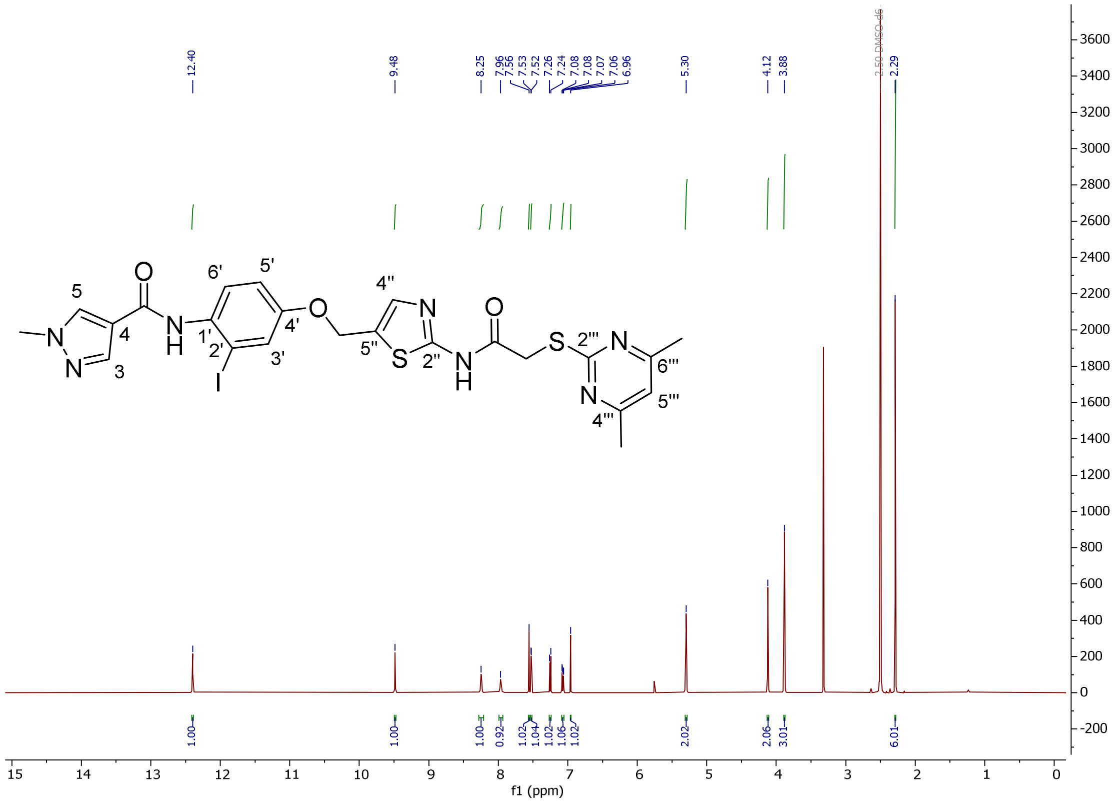

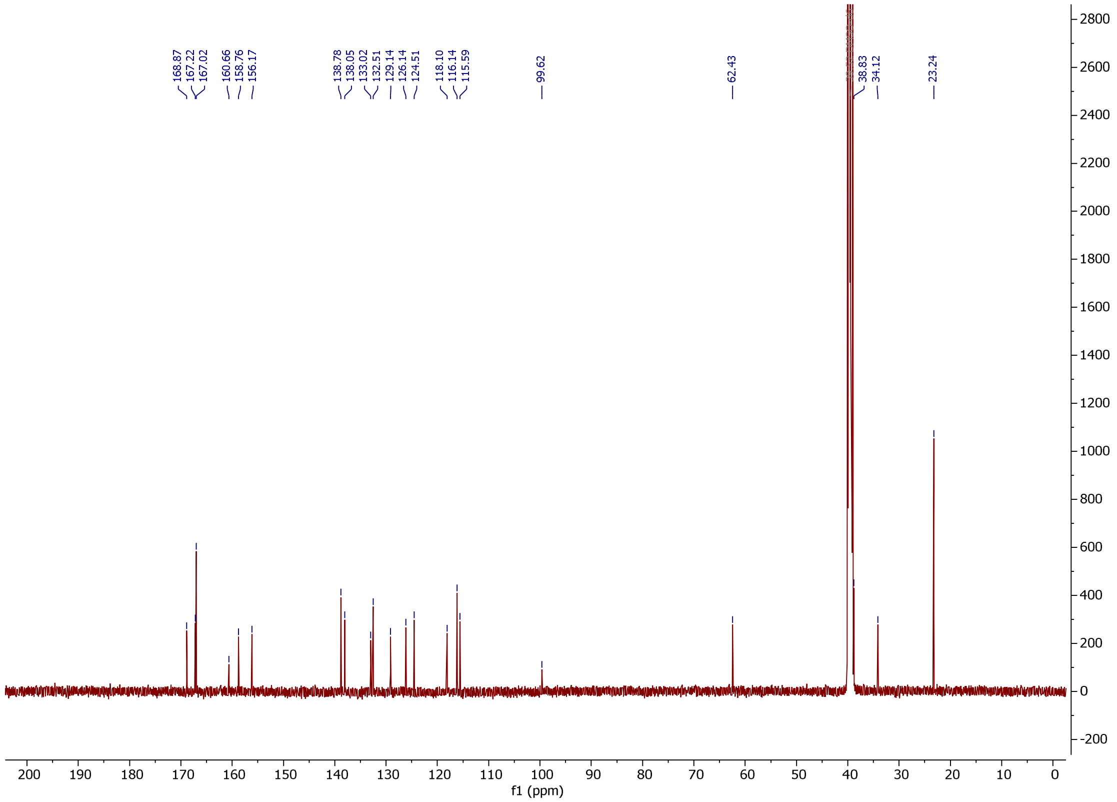

^{1}H (400 MHz; $(CD_3)_2SO$) and ^{13}C (101 MHz; $(CD_3)_2SO$) NMR spectra of **RW-99**

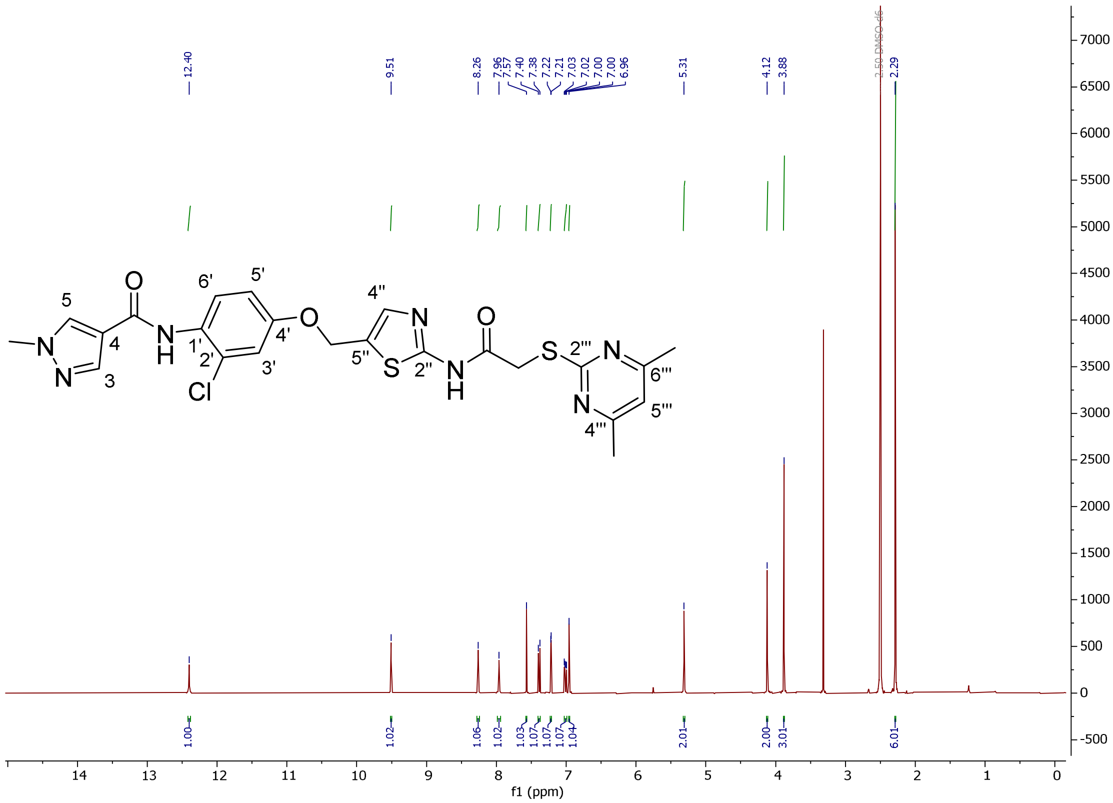

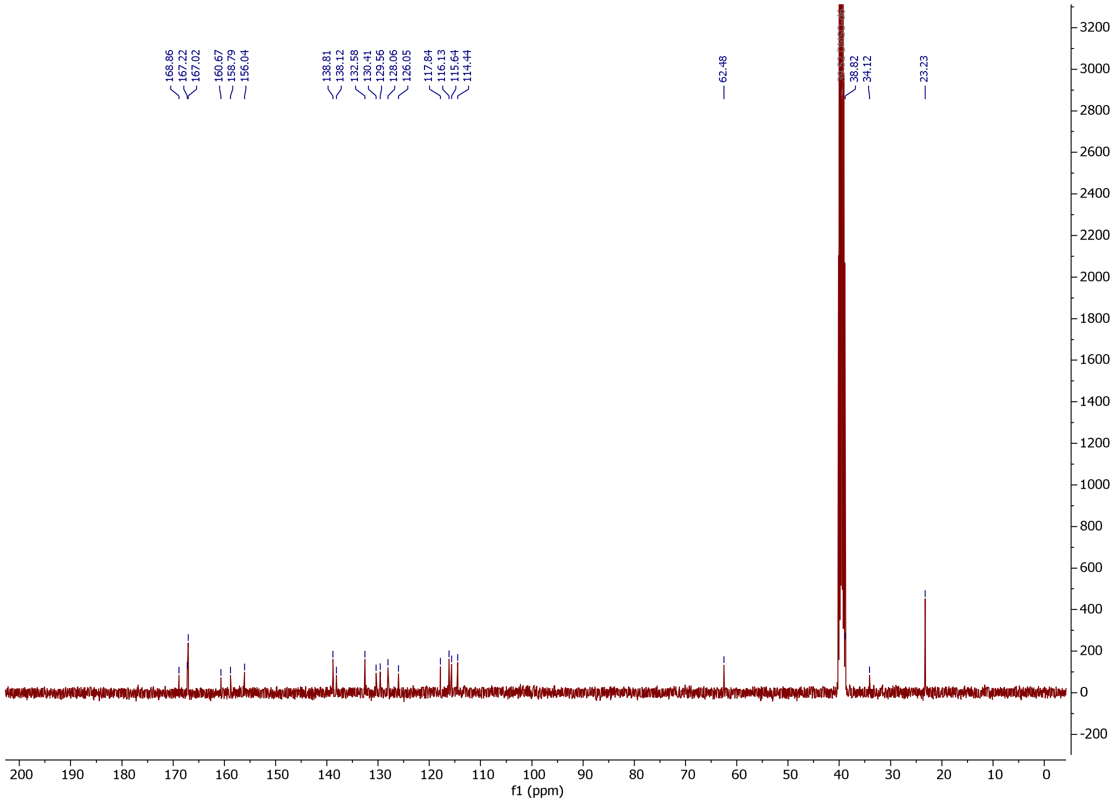

HPLC chromatogram of compound **Yang_28e**

Chromatogram and Results

Injection Details

Injection Name:	**fm76**	*Run Time (min):*	**9,99**
Vial Number:	**Vial:98**	*Injection Volume:*	**10,00 µL**
Instrument Method:	**50 ACN 50 Wasser 210nm**	*Wavelength:*	**210 nm**
Column:	**Zorbax Eclipse Plus C18 4,6x 150mm, 5µm**	*Flow rate:*	**1,0 ml/min**
Injection Date/Time:	**06.Okt.20 19:24**	*Column Temperatur:*	**35,0 °C**
		Pump Channel A: 50,0	**ACN**
		Pump Channel D: 50,0	**ACN**

Chromatogram

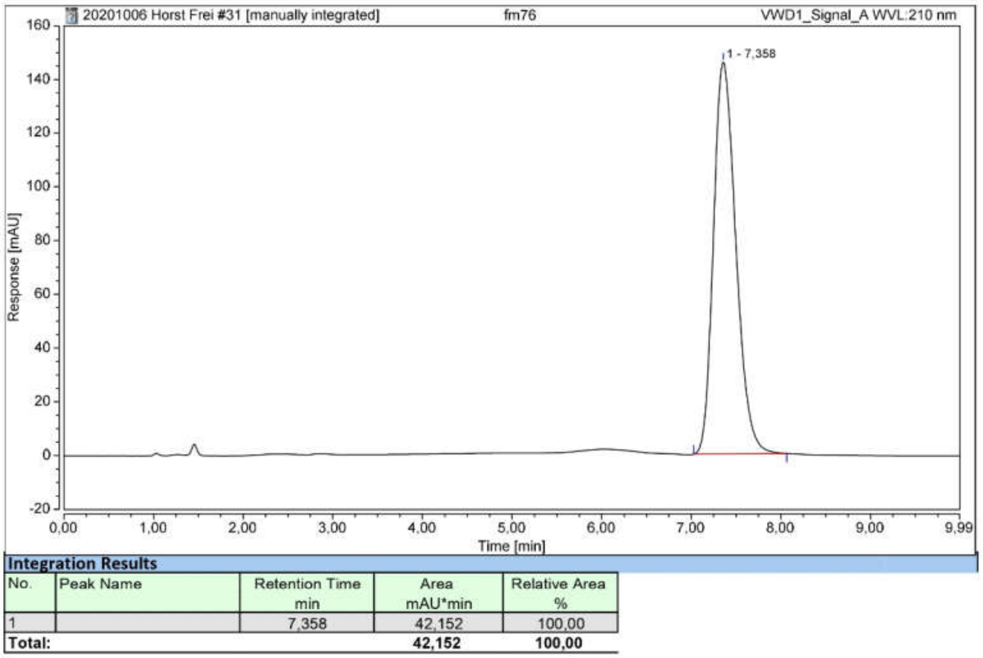

Integration Results

No.	Peak Name	Retention Time min	Area mAU*min	Relative Area %
1		7,358	42,152	100,00
**Total:**			**42,152**	**100,00**

HPLC chromatogram of **FM345**

Chromatogram and Results

Injection Details

Injection Name:	MF-345	*Run Time (min):*	9,99
Vial Number:	Vial:14	*Injection Volume:*	5,00
Injection Type:	Unknown	*Wavelength A:*	210
Column:	Zorbax SB C18 3,5µm 4,6x100mm 861953-902	*Wavelength B:*	254
Instrument Method:	50 AcN 50 Wasser		
Processing Method:	SG-094	*Flow rate:*	1,200 mL/min
Injection Date/Time:	26.Jul.23 13:43	*Column Temperatu*	35,0 °C
Pump Channel A:	50,00 AcN		
Pump Channel B:			
Pump ChannelC:			
Pump Channel D:	50 Wasser		

Chromatogram

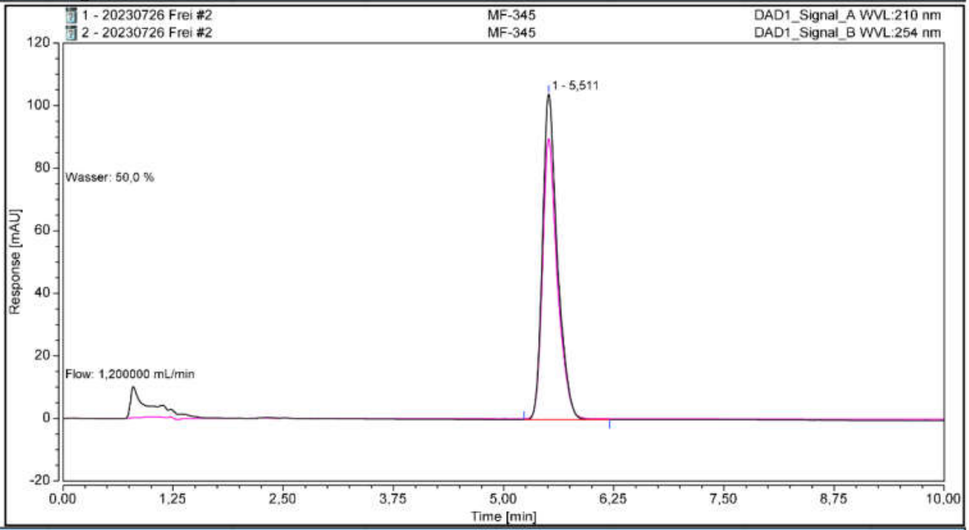

Integration Results

210nm

No.	Peak Name	Retention Time min	Area mAU*min	Relative Area %
1		5,511	20,367	100,00
**Total:**			**20,367**	**100,00**

254mn

No.	Peak Name	Retention Time min	Area mAU*min	Relative Area %
1		5,511	17,514	100,00
**Total:**			**17,514**	**100,00**

Chromeleon (c) Dionex
Version 7.2.9.11323

HPLC chromatogram of **FM352**

Chromatogram and Results

Injection Details

Injection Name:	**MF-352**	*Run Time (min):*	**9,99**
Vial Number:	**Vial:15**	*Injection Volume:*	**5,00**
Injection Type:	**Unknown**	*Wavelength A:*	**210**
Column:	**Zorbax SB C18 3,5µm 4,6x100mm 861953-902**	*Wavelength B:*	**254**
Instrument Method:	**50 AcN 50 Wasser**		
Processing Method:	**SG-094**	*Flow rate:*	**1,200 mL/min**
Injection Date/Time:	**26.Jul.23 13:53**	*Column Temperatu*	**35,0 °C**
Pump Channel A:	**50,00** AcN		
Pump Channel B:			
Pump ChannelC:			
Pump Channel D:	**50** Wasser		

Chromatogram

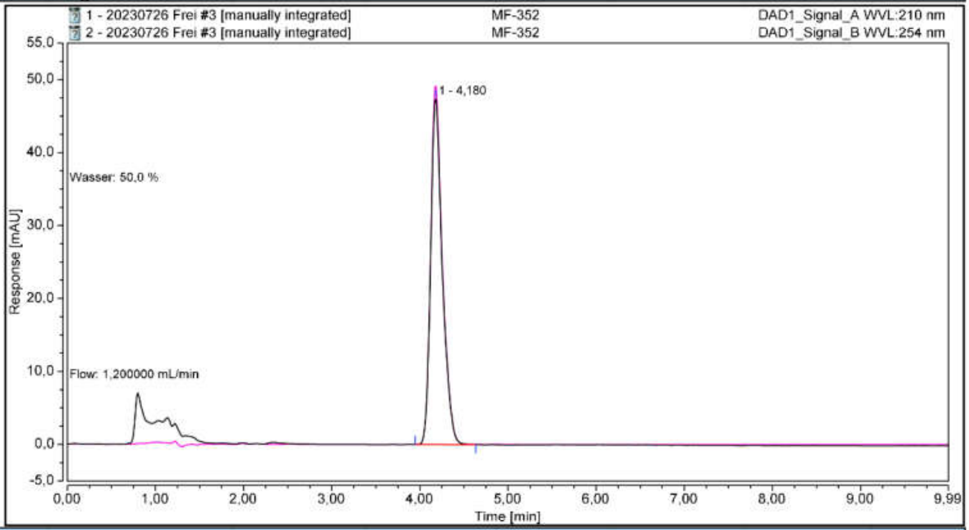

Integration Results

210nm

No.	Peak Name	Retention Time min	Area mAU*min	Relative Area %
1		4,180	7,290	100,00
**Total:**			**7,290**	**100,00**

254mn

No.	Peak Name	Retention Time min	Area mAU*min	Relative Area %
1		4,180	7,559	100,00
**Total:**			**7,559**	**100,00**

Reinheit Honey/Integration

Chromeleon (c) Dionex
Version 7.2.9.11323

HPLC chromatogram of **FM358**

Chromatogram and Results

Injection Details

Injection Name:	**MF-358**	*Run Time (min):*	**9,99**
Vial Number:	**Vial:62**	*Injection Volume:*	**5,00**
Injection Type:	**Unknown**	*Wavelength A:*	**210**
Column:	**Zorbax SB C18 3,5µm 4,6x100mm 861953-902**	*Wavelength B:*	**254**
Instrument Method:	**50 AcN 50 Wasser**		
Processing Method:	**SG-094**	*Flow rate:*	**1,200 mL/min**
Injection Date/Time:	**26.Jul.23 14:25**	*Column Temperatu*	**35,0 °C**
Pump Channel A:	**50,00** AcN		
Pump Channel B:			
Pump ChannelC:			
Pump Channel D:	**50** Wasser		

Chromatogram

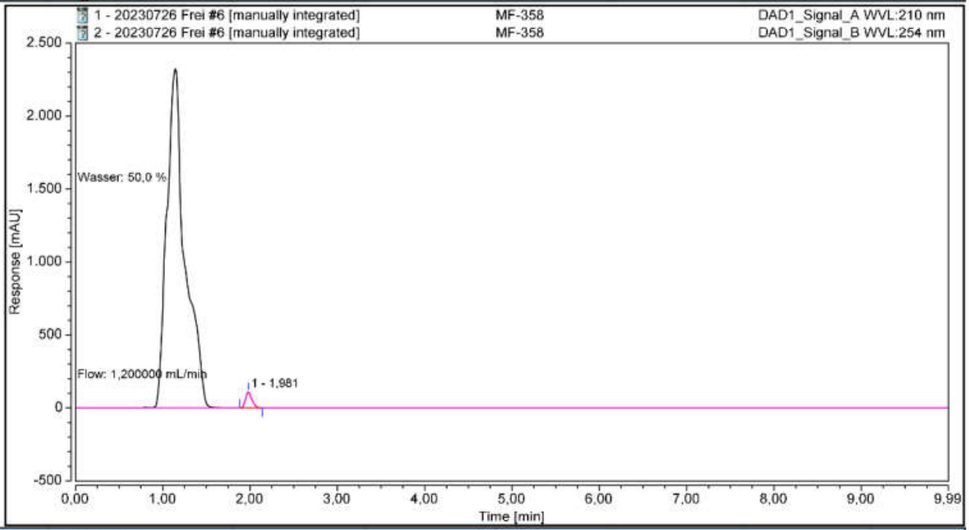

Integration Results

210nm

No.	Peak Name	Retention Time min	Area mAU*min	Relative Area %
1		1,981	8,851	100,00
**Total:**			**8,851**	**100,00**

254mn

No.	Peak Name	Retention Time min	Area mAU*min	Relative Area %
1		1,981	9,350	100,00
**Total:**			**9,350**	**100,00**

HPLC chromatogram of **FM368**

Chromatogram and Results

Injection Details

Injection Name:	**fm 368**	*Run Time (min):*	**14,99**
Vial Number:	**Vial:7**	*Injection Volume:*	**10,00**
Injection Type:	**Unknown**	*Wavelength A:*	**210**
Column:	**Eclipse Plus C18 5µm 4,6x 150mm USUXB17231**	*Wavelength B:*	**254**
Instrument Method:	**70 AcN 30 Wassser**		
Processing Method:	**SG-094**	*Flow rate:*	**1,200 mL/min**
Injection Date/Time:	**23.Feb.24 09:02**	*Column Temperatu*	**50,0 °C**
Pump Channel A:	**70,00** Acetonitril		
Pump Channel B:			
Pump ChannelC:	Phosphatpuffer pH 5		
Pump Channel D:	**30** Wasser		

Chromatogram

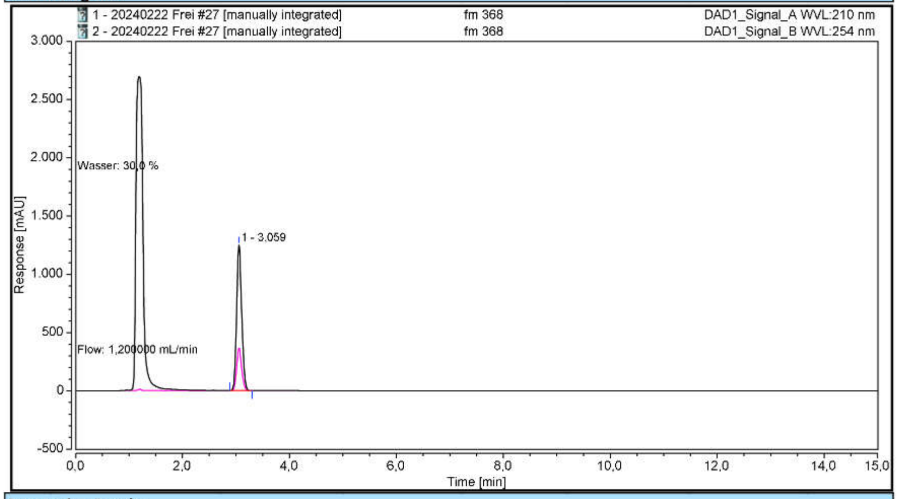

Integration Results

210nm

No.	Peak Name	Retention Time min	Area mAU*min	Relative Area %
1		3,059	136,406	100,00
**Total:**			**136,406**	**100,00**

254mn

No.	Peak Name	Retention Time min	Area mAU*min	Relative Area %
1		3,059	40,051	100,00
**Total:**			**40,051**	**100,00**

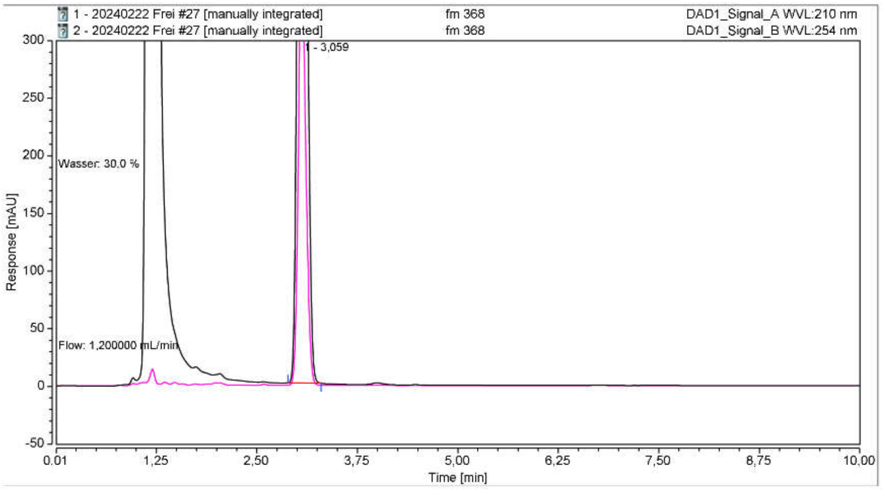

IR spectrum of lead compound **Yang_28e**

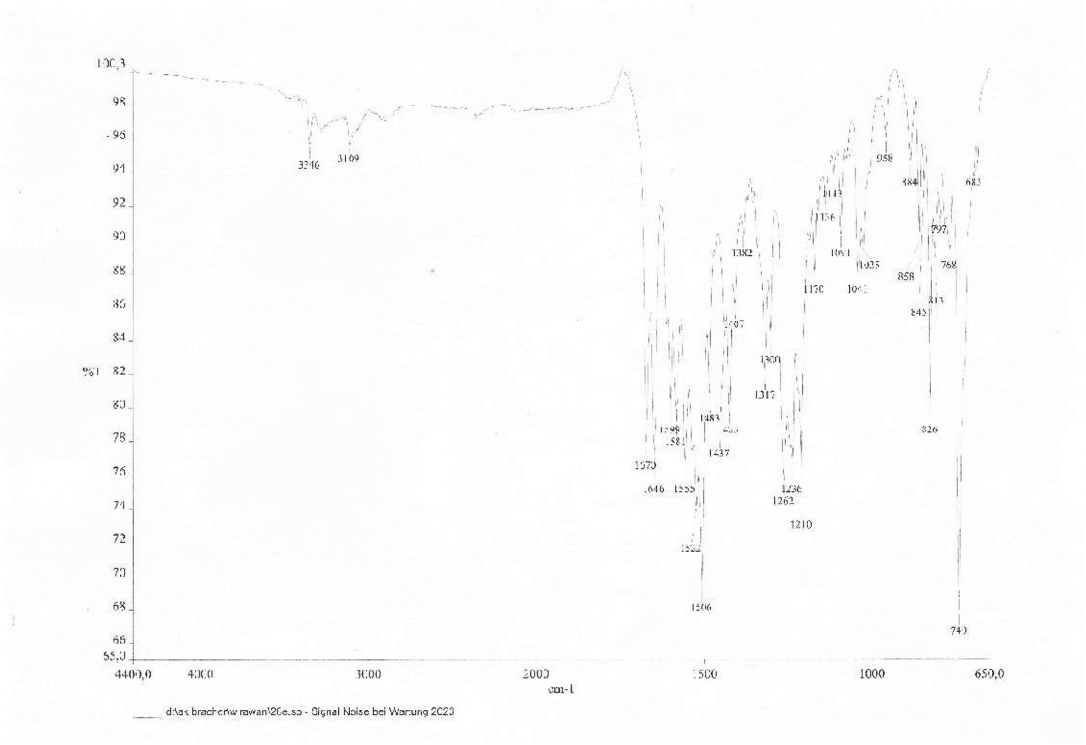

IR spectrum of lead compound **Yang_24a**

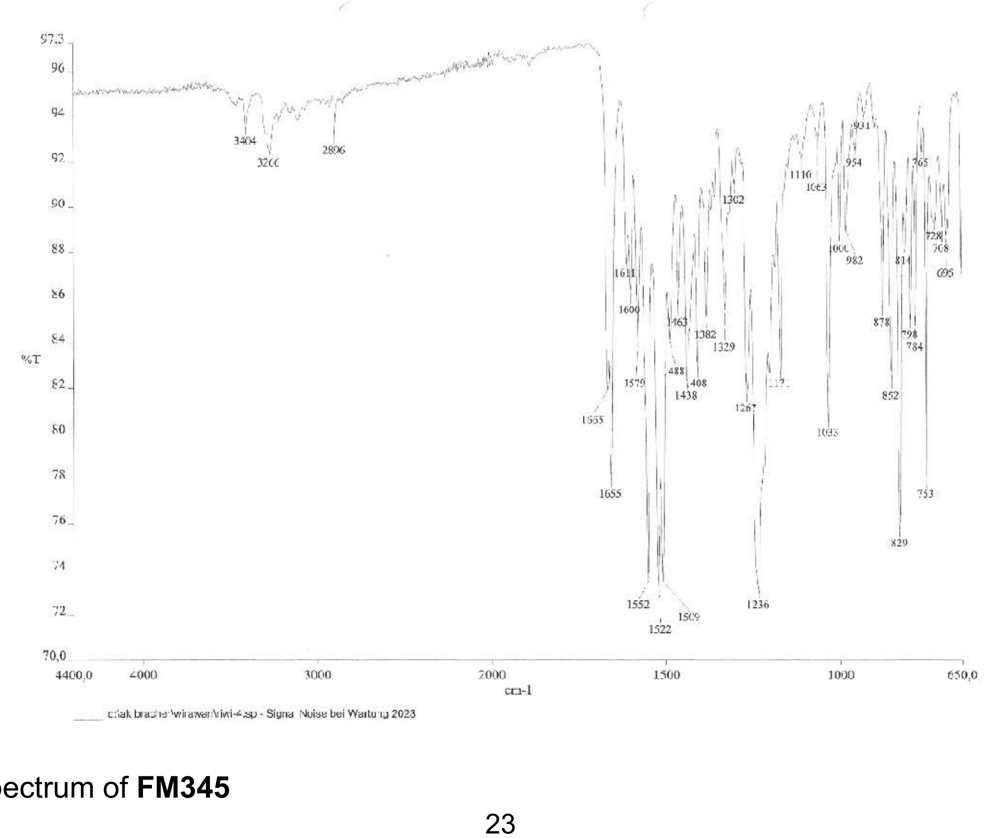

IR spectrum of **FM345**

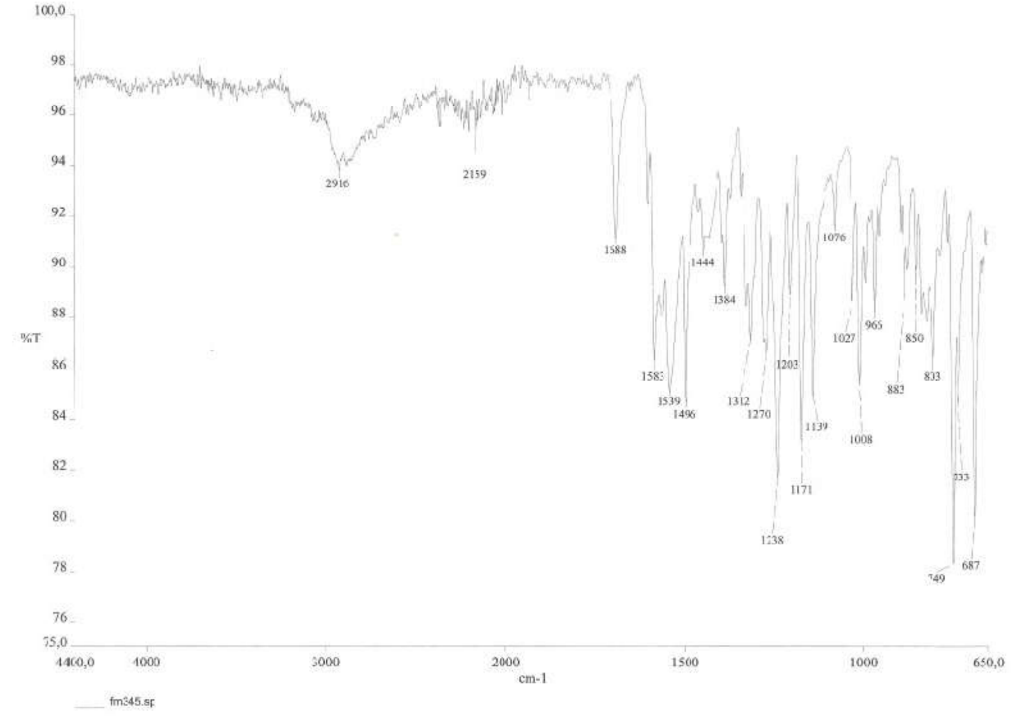

IR spectrum of **FM352**

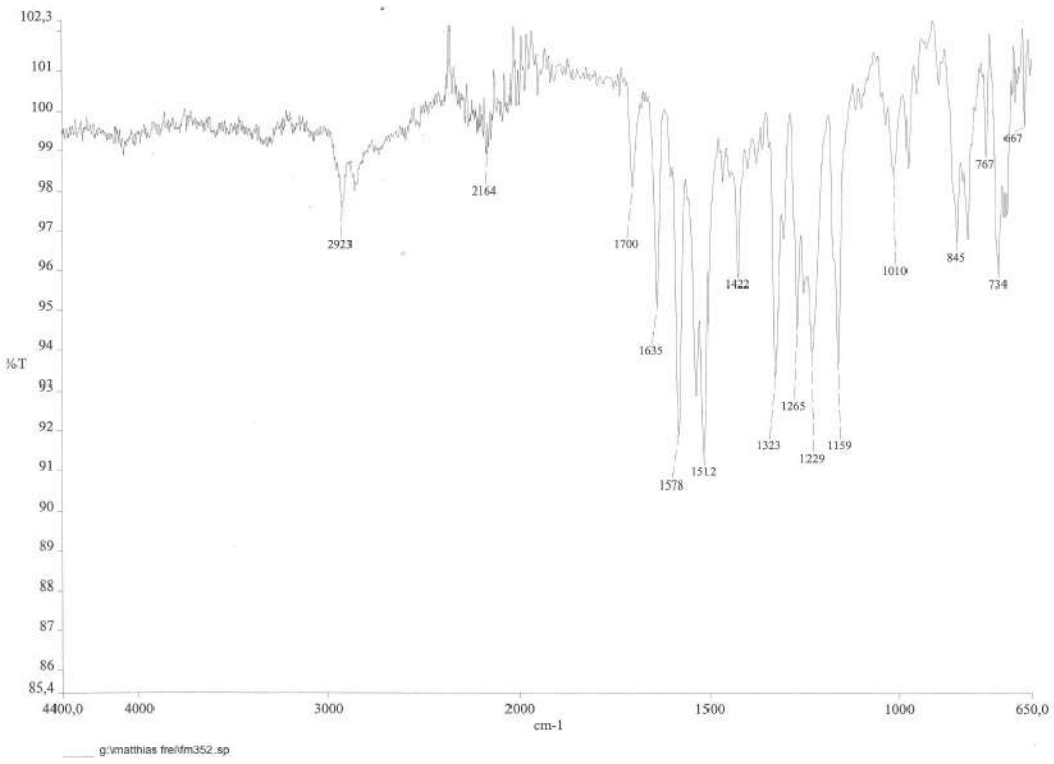

IR spectrum of **FM358**

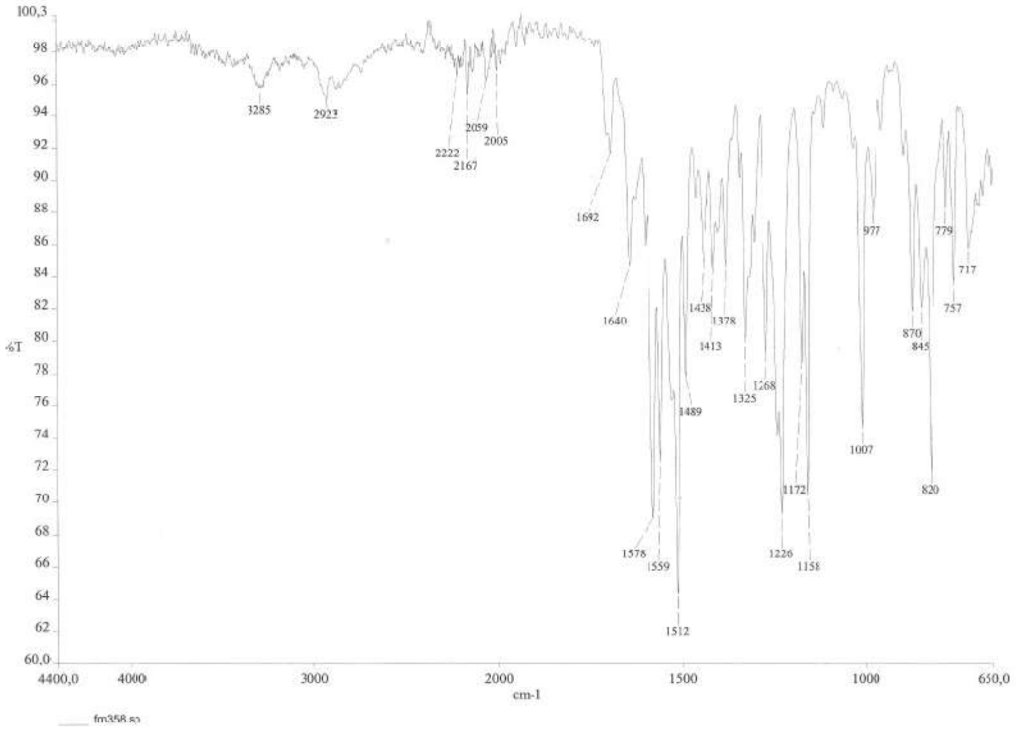

IR spectrum of **FM368**

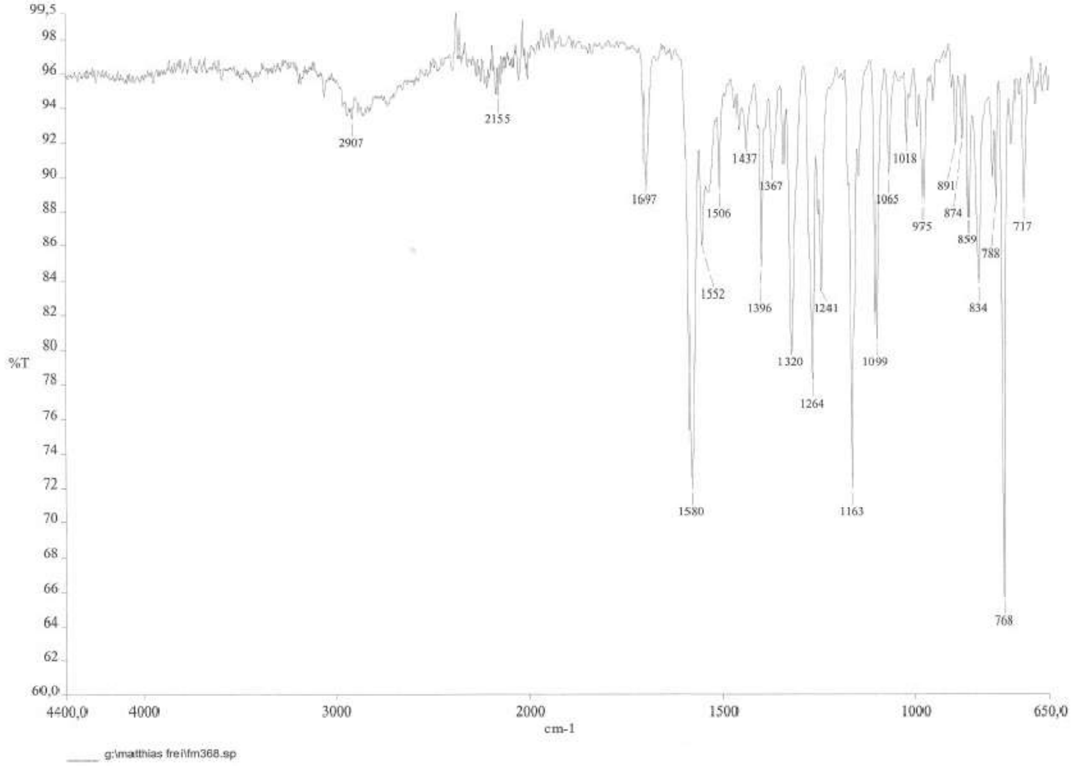

IR spectrum of **RW-93**

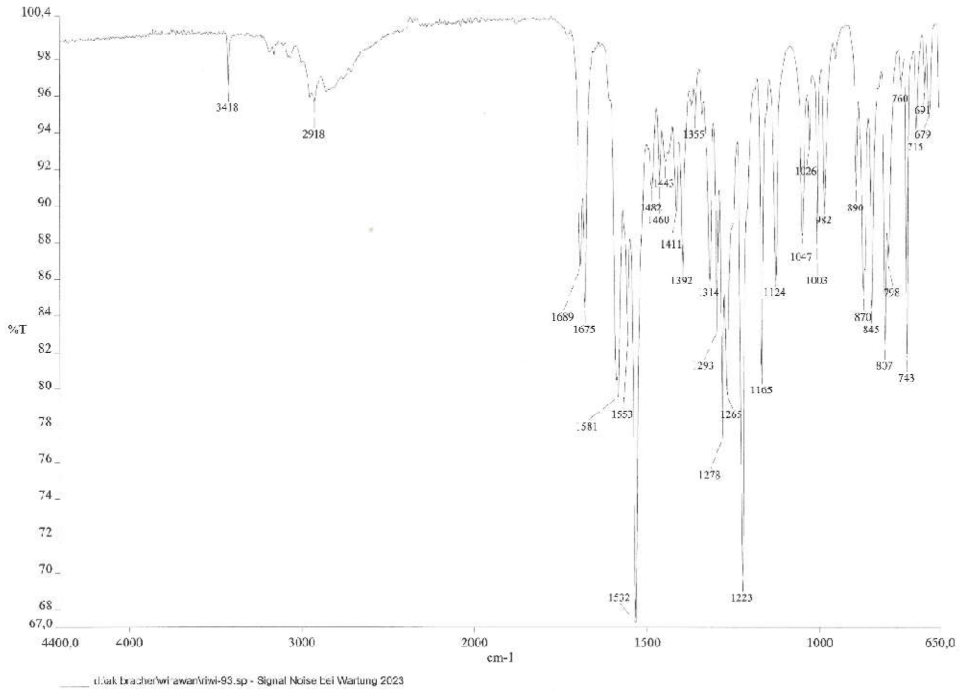

IR spectrum of **RW-95**

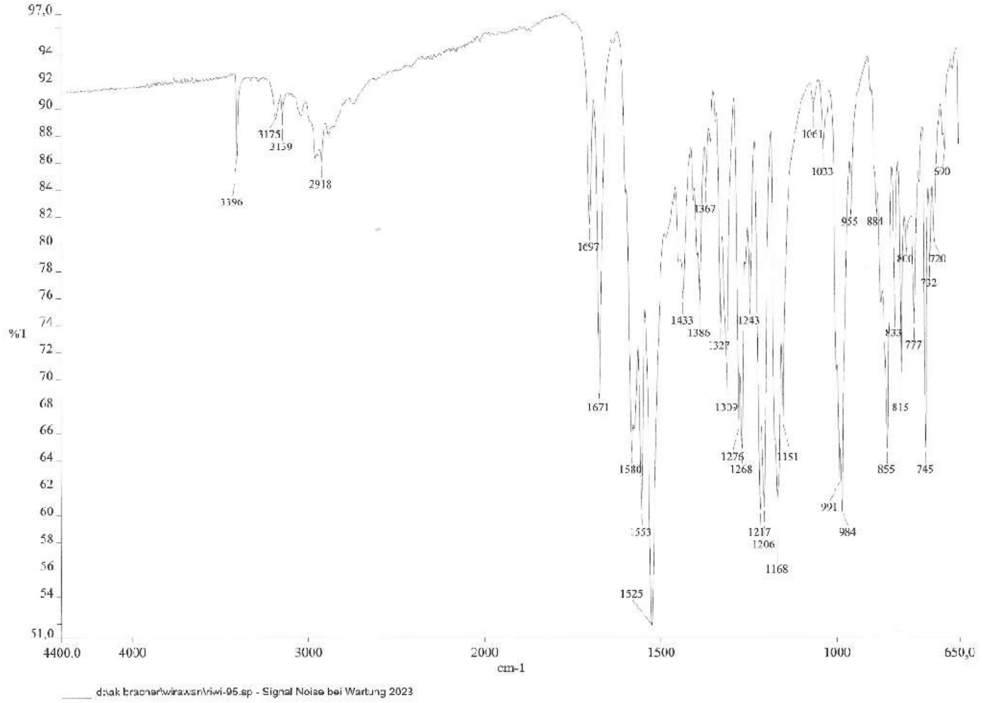

IR spectrum of **RW-99**

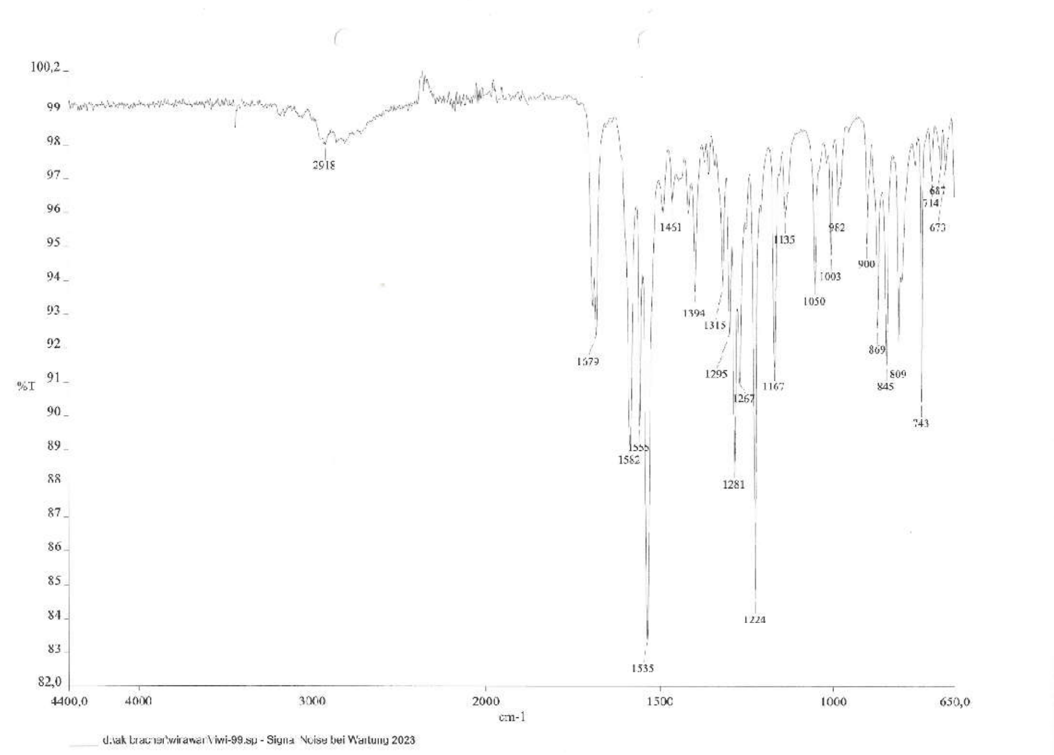

Cell viability data of **RW-93**

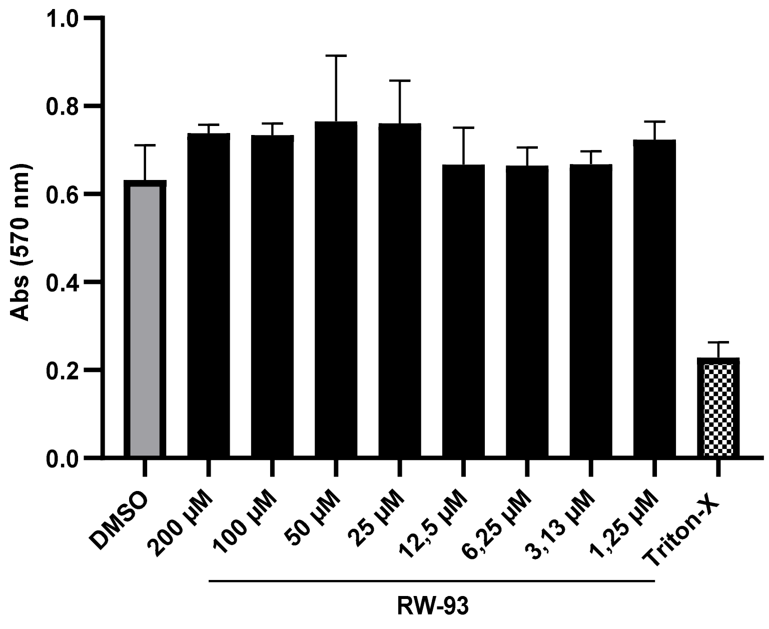

Figure S1: Cell viability data of RW-93. The acute cytotoxicity of **RW-93** was determined using a colorimetric MTT assay. HL-60 cells were treated with **RW-93** at various concentrations with three technical replicates. Triton-X was used as a positive control. No significant changes in the cell viability relative to DMSO control was determined following treatment with up to 200 µM of **RW-93**.
